# Synaptic weight dynamics underlying memory consolidation: Implications for learning rules, circuit organization, and circuit function

**DOI:** 10.1073/pnas.2406010121

**Published:** 2024-10-04

**Authors:** Brandon J. Bhasin, Jennifer L. Raymond, Mark S. Goldman

**Affiliations:** ^a^Department of Bioengineering, Stanford University, Stanford, CA 94305; ^b^Center for Neuroscience, University of California, Davis, CA 95616; ^c^Department of Neurobiology, Stanford University School of Medicine, Stanford, CA 94305; ^d^Department of Neurobiology, Physiology, and Behavior, University of California, Davis, CA 95616; ^e^Department of Ophthalmology and Vision Science, University of California, Davis, CA 95616

**Keywords:** computational model, cerebellum, heterosynaptic plasticity, memory consolidation

## Abstract

Systems consolidation is a common feature of learning and memory systems, in which a long-term memory initially stored in one brain region becomes persistently stored in another region. We studied the dynamics of systems consolidation in simple circuit architectures with two sites of plasticity, one in an early-learning and one in a late-learning brain area. We show that the synaptic dynamics of the circuit during consolidation of an analog memory can be understood as a temporal integration process, by which transient changes in activity driven by plasticity in the early-learning area are accumulated into persistent synaptic changes at the late-learning site. This simple principle naturally leads to a speed-accuracy tradeoff in systems consolidation and provides insight into how the circuit mitigates the stability-plasticity dilemma of storing new memories while preserving core features of older ones. Furthermore, it imposes two constraints on the circuit. First, the plasticity rule at the late-learning site must stably support a continuum of possible outputs for a given input. We show that this is readily achieved by heterosynaptic but not standard Hebbian rules. Second, to turn off the consolidation process and prevent erroneous changes at the late-learning site, neural activity in the early-learning area must be reset to its baseline activity. We provide two biologically plausible implementations for this reset that propose functional roles in stabilizing consolidation for core elements of the cerebellar circuit.

## Significance

How are memories transformed over time? We propose a simple organizing principle for how long-term memories are moved from an initial to a final site of storage. We show that successful transfer occurs when the late site of memory storage is endowed with synaptic plasticity rules that stably accumulate changes in activity occurring at the early site of memory storage. We instantiate this principle in a simple computational model that is representative of brain circuits underlying a variety of behaviors. The model suggests how a neural circuit can store new memories while preserving core features of older ones and proposes functional roles for core elements of the cerebellar circuit.

Memory systems transform transiently present information into a more persistent form. In short-term (working) memory, transient spiking activity of input neurons is transformed into persistent activity in downstream short-term memory-storing circuits (1, 2). In long-term memory, memories stored transiently through neural plasticity at one site may become stored persistently at another site, through a process known as systems consolidation (3, 4). Systems consolidation is a common feature of learning and memory systems, including declarative memory (5), fear conditioning (6), and motor skill learning (7). It is thought to help memory systems navigate the “stability-plasticity” dilemma (8, 9)—balancing the need to have capacity for new memories and the tendency of new memories to overwrite old ones, which could lead to catastrophic forgetting (10, 11).

In working memory, the transformation of transient representations into a more persistent form can be characterized by well-established computational principles (1, 12–17). For the storage of analog (graded or continuous-valued) memories, this transformation can be accomplished through temporal integration of transient activity in the input to a circuit into persistent changes in circuit output, with the set of possible stored activity patterns forming a continuous set of stable patterns.

**Fig. 1.**
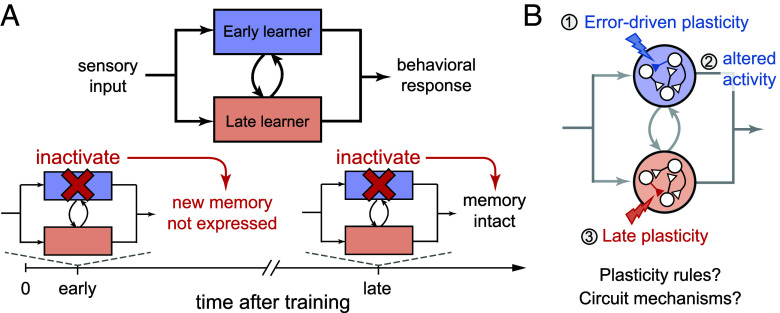
Schematic of systems consolidation. (*A*) Learned changes in the response to a sensory input occur first in an early-learning area, but are transferred over time to a late-learning area that becomes the sole site required for expression of learning, as revealed by inactivation experiments. (*B*) Feedback about behavioral errors drives synaptic plasticity in the early-learning area 1), leading to changes in activity 2) that in turn drive secondary plasticity in the late-learning area 3). Here, we investigate the dynamical mechanisms underlying this consolidation process and their implications for circuit organization and function.

For systems consolidation, overarching computational principles describing the transformation of transient into persistent representations are less well established. Qualitatively, the standard view of systems consolidation suggests that transient plasticity in an early-learning brain area results in altered neural activity that then triggers the induction of persistent changes in the late-learning area following training (4, 6, 7, 18–20). Through this process, the expression of learning becomes robust to inactivation of the early-learning area (Fig. 1 *A* and *B*).

We investigated the dynamics of systems consolidation in a model of a simple circuit that captures essential features of the systems consolidation of error-driven learning in brain areas such as the cerebellum (21–23), striatum (24–29), and amygdala (6, 30). Building upon previous models of systems consolidation of oculomotor learning (31–37), we show that systems consolidation can be framed as a process of temporal integration, in which transient changes at the initial site of plasticity are integrated into changes at the final site. We show that this simple principle places strong constraints on learning that are met only by certain classes of plasticity rules, and provides insight into how a circuit may mitigate the stability-plasticity dilemma. Further, within the context of cerebellar systems consolidation, our results extend previous proposals for how molecular layer interneurons and nucleo-olivary pathways, core circuit elements not included in traditional models, may serve to regulate cerebellar cortical plasticity (33, 34, 38). Specifically, we show how these pathways may be necessary for stabilizing the dynamics of systems consolidation.

## Results

### A Simple Circuit Model of Systems Consolidation.

We modeled a simple circuit that learns the analog-valued gain of an input-to-output transformation. This gain is determined jointly by a direct and an indirect pathway through the circuit. Errors in behavioral performance drive plasticity at an early-learning site in the indirect pathway, which is then consolidated at a late-learning site in the direct pathway.

**Fig. 2.**
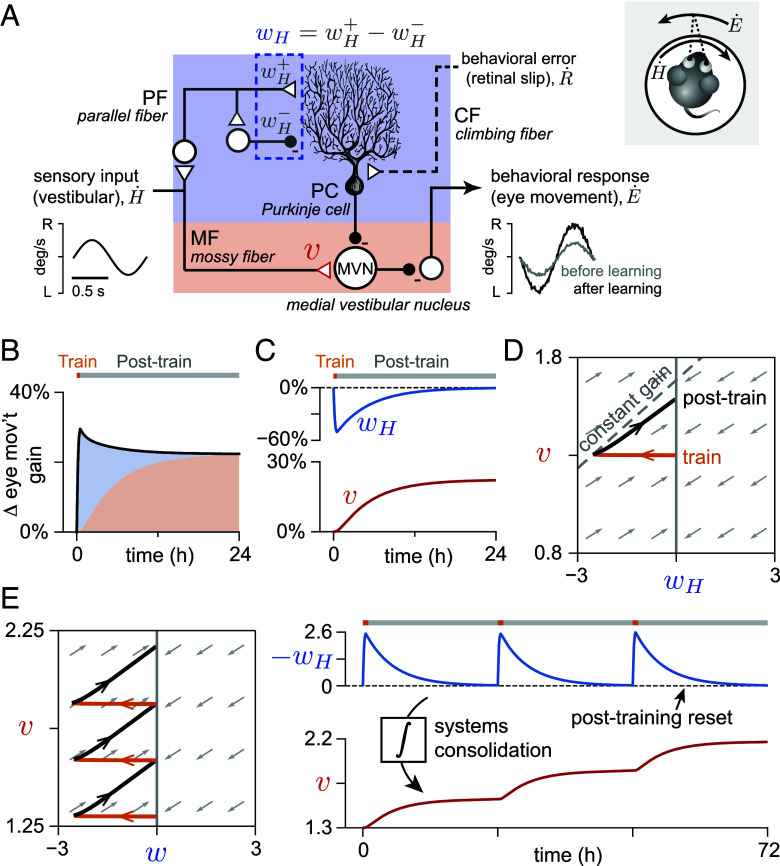
Synaptic weight dynamics in a representative feedforward-architecture circuit model of systems consolidation correspond to a temporal integration process. (*A*) The microcircuit underlying oculomotor learning alters the amplitude (“gain”) of the reflexive eye movement response to sensory (vestibular) input. Incorrect eye movement amplitude causes image motion on the retina (“retinal slip”). Instructive signals carrying information about behavioral errors, i.e., retinal slip, drive changes in weight, $w_{H}$, at an early-learning site in the cerebellar cortex (blue shaded area), with $w_{H}$ representing the difference between excitatory ($w_{H}^{+}$) and inhibitory ($w_{H}^{-}$) synaptic strengths. Over time, expression of learning becomes dependent only on the weight v at a late-learning site in the brainstem (red shaded area). Excitatory synapses are represented by open triangles, inhibitory synapses by filled circles. (*B* and *C*) Simulation of model for 30 min of training (orange block) to increase the input-to-output gain of the eye movement response, followed by a 23.5 h post-training period in the dark (gray block). During the post-training period, the model received no information about errors. (*B*) Change in the gain of the eye movement response (black line), with shading showing contributions from the early- (blue) and late-learning (red) sites. (*C*) Change in weights $w_{H}$ (blue) and v (red). (*D*) Trajectory of synaptic weights during the training (orange) and post-training (black) periods. Gray arrows show the analytically calculated, approximate instantaneous direction in which a weight configuration at a given point will evolve without information about errors. All trajectories tend toward some marginally stable point along the line $w_{H}=0$ (thick gray line). A trajectory resulting in perfect consolidation would follow the “constant gain” line (gray dashed line). (*E*) Consolidation corresponds to a temporal integration process in which transient changes in activity at the early-learning site, driven by plasticity at $w_{H}$, are accumulated into persistent changes in v. Panels show trajectory and dynamics in synaptic weight space (*Left*) and over time (*Right*) in a simulation of three consecutive days of the training protocol in (*B*–*D*).

To make the analysis more concrete, we consider the specific example of oculomotor learning. Our focus is not on the intricacies of oculomotor learning specifically but the principles of consolidation that recur in circuit architectures throughout the brain. Nevertheless, our model captures many important features of oculomotor learning. The gain of eye movement responses to vestibular or visual stimuli can be adaptively modified by learning so as to attain any value within an analog range (Fig. 2*A*; see *Feedforward Sensorimotor Circuit Model*; 39). Expression of this learned change in the sensory-to-motor transformation initially depends on the cerebellar cortex, but becomes cerebellum-independent within 24 h following post-training (40–44). Learning and consolidation occur over timescales that are long compared to the eye movement responses to sensory stimuli, hence we modeled the latter as instantaneous.

We first studied a model of the circuit in which the input-to-output transformation was purely feedforward. In the context of cerebellum-dependent learning, this is consistent with the classical Marr-Albus-Ito model (45–47) and previous computational studies of the consolidation of oculomotor learning (31–33, 36, 37). We simulated neural activity and changes in synaptic weights in the feedforward circuit model during a training and post-training period. During the training period, the model receives instructive signals about behavioral performance, which control the induction of plasticity at the early-learning site. For the case of oculomotor learning, these instructive signals reflect behavioral errors resulting from failures of eye movements to stabilize images on the retina, carried by the climbing fiber input to the cerebellar cortex. Such signals induce a decrease in the weight $w_{H}$ at the early-learning site, reducing the inhibitory output from the indirect (cerebellar cortical) pathway and thereby increasing the overall gain of the sensory-to-motor transformation (Fig. 2 *B* and *C*; 41, 48, 49, 50).

During the subsequent post-training period, information about behavioral errors is not available (the experimental subject is placed in the dark to eliminate visual feedback about the stabilization of images on the retina), and we assume $w_{H}$ decays back to a baseline value of zero (Fig. 2*C*). This baseline value of zero for $w_{H}$ can be interpreted as a balance of excitation and inhibition at the early-learning site. The decay of plasticity at $w_{H}$ is consistent with electrophysiological measurements (41), the experimental observation that memory expression becomes cerebellum-independent over time (40, 42, 43, 51), and previous models (31–33, 37).

Because the synaptic changes induced at the early-learning site during the training period are transient, successful consolidation requires the induction of persistent changes at the late-learning site (31–33, 36, 37). Consolidation is known to depend on neural activity during the post-training period (52), which presumably induces the plasticity at the late-learning site (41). To achieve this, we implemented a heterosynaptic plasticity rule for the late-learning weight v of the form

$$\begin{array}{c} \Delta v\propto -\;\text{MF}(\;\text{PC}-\;{\text{PC}}_{0}), \end{array}$$

in which weight changes are driven by the correlation of direct pathway input (in the cerebellar context: mossy fiber, MF) with early-learning area output (Purkinje cell, PC) relative to baseline (PC_0_) (see *Learning rules* for the full equation), consistent with previous models of oculomotor learning (31–36). As a result, altered post-training activity at the early-learning site induced an increase in v to a new steady-state value (Fig. 2*C*). This change persisted even as $w_{H}$ returned to its baseline, supporting a persistent increase in the input-to-output gain of the circuit (Fig. 2 *B* and *C*).

### Dynamical Principles of Systems Consolidation.

To understand the features of the synaptic weight dynamics that support successful systems consolidation, we plotted the trajectory describing the joint evolution of the early- and late-learning weights. Initially, training induces a change at the early-learning site, decreasing the weight $w_{H}$ from its baseline value and moving the weights $\left(w_{H},v\right)$ (Fig. 2*D*, orange trajectory) to a point corresponding to an increased input-to-output gain of the eye movement response (Fig. 2*D*, dashed “constant gain” line). During the post-training period, the early-learning weight decays to baseline, but the late-learning weight is driven to a new steady-state value that preserves some of the increase in the input-to-output gain accrued during training (Fig. 2*D*, black trajectory). A nearly parallel trajectory will be followed by the evolution of the weights during the post-training period for any weight configuration reached during training (Fig. 2*D*, gray arrows), with all trajectories approaching some steady-state value along a line in synaptic weight space (Fig. 2*D*, dark vertical line). Because the weight dynamics are at steady state whenever the activity of the early-learning area is at baseline, which occurs when the early-learning weight $w_{H}$ decays to zero, there is a continuum of values that can be stably maintained by the late-learning weight v (*SI Appendix*, section S1). Therefore, for a given initial value of v before training, the final value reached after consolidation varies in a graded manner with the magnitude of the change in the early-learning weight $w_{H}$ during training. This enables the circuit to learn and maintain any graded amplitude input-to-output gain across multiple learning events (Fig. 2*E* and *SI Appendix*, Fig. S1 and section S1).

These dynamics suggest an intuitive computational principle: Systems consolidation of analog memories corresponds to a temporal integration process in which the late-learning weight stably accumulates changes induced during training at the early-learning site. For the simple feedforward model we have been examining, this can be visualized directly in the space of synaptic weights as an integration of transient changes in $w_{H}$ into persistent changes in v (Fig. 2*E*). More generally, changes in the output of the early-learning area, driven by plasticity at $w_{H}$, are accumulated into weight changes at the late-learning site (see below for more complex circuit architectures).

To obey this principle, two conditions must be satisfied. First, the rule governing plasticity at the late-learning site must support the stable accumulation of persistent weight changes and corresponding continuum of input-to-output gains. Second, the circuit must reset the output of the early-learning site during the post-training period so that the accumulation in the late-learning weight stops. Here, we lay out the implications of the principle of consolidation as integration, starting with circuit function. Then, we show how the two simple conditions stated above place strong constraints on the plasticity rules and circuit features needed to support systems consolidation.

### Consolidation Exhibits Diffusive Drift in the Absence of Information about Errors, Suggesting a Speed-Accuracy Tradeoff for Consolidation.

**Fig. 3.**
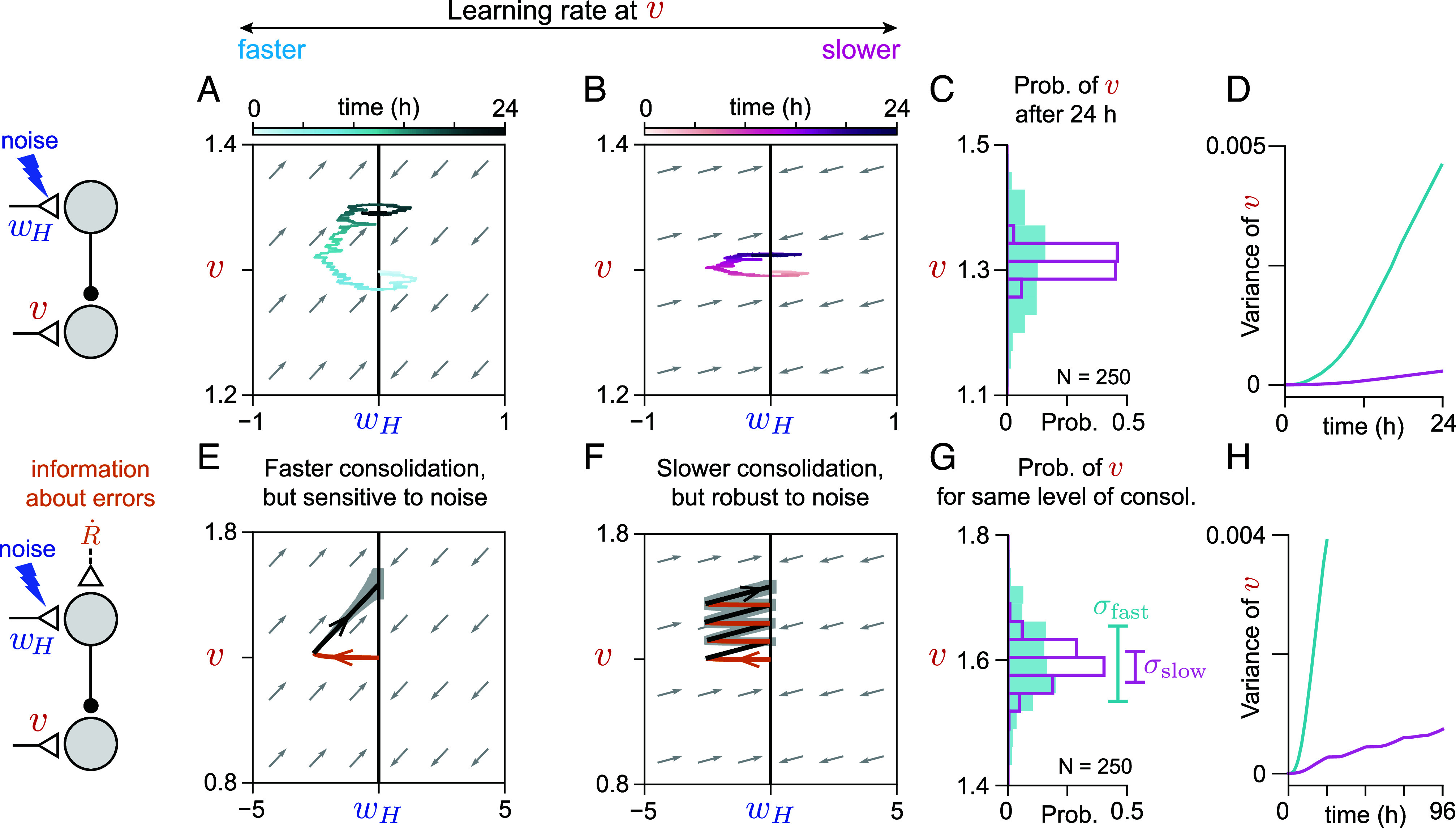
Diffusive drift of consolidated memory implies a tradeoff between speed of consolidation and sensitivity to noise. (*A* and *B*) Trajectories of synaptic weights in response to periodic random perturbations in the early-learning weight $w_{H}$ when the learning rate at v is relatively fast (*A*) or slow (*B*) (light to dark colors show progression of time). (*C*) Distribution of the value of the late-learning weight v at the end of 24 h of random perturbations of $w_{H}$ for N=250 simulations (see *Materials and Methods* for details), using the slower (open magenta bars) or faster (filled cyan bars) learning rates at v depicted in panels (*A*) and (*B*), respectively. (*D*) Time course of the variance of the distributions in (*C*). (*E* and *F*) Mean trajectories of synaptic weights during training (orange) and subsequent post-training periods (black) with the same perturbations as in (*A* and *B*) (N=250 simulations). Gray band indicates the range of trajectories within one SD of the mean at each time point. The learning rates in panels (*E*) and (*F*) were the same as in panels (*A*) and (*B*), respectively. Trajectories in panel (*F*) reach approximately the same mean level of consolidation as those in panel (*E*) after four consecutive days of training. (*G*) Distribution of v at the end of training for simulations shown in (*E*) (filled cyan bars) and (*F*) (open magenta bars). Vertical bars show SDs of the distributions, $\sigma_{\;\text{fast}}$ and $\sigma_{\;\text{slow}}$ respectively. (*H*) Time course of the variance of v due to perturbations for simulations shown in (*E*) (cyan) and (*F*) (magenta). With a slower learning rate at v, the circuit accumulates less noise. The scalloping in the variance for the simulations in (*F*) is due to the effect of feedback about errors during each training session.

Because the dynamics of systems consolidation can be understood as temporal integration, systems consolidation exhibits properties that have been well characterized in other kinds of integrators, such as diffusive drift in the value being memorized due to the accumulation of noise, which has been found in neural integrator circuits implementing working memory (12, 16, 53–55). In our model, noise in the neural activity driving plasticity at the late-learning weight v is accumulated, leading the input-to-output gain of the circuit to drift. In the absence of information about behavioral errors (e.g. during the post-training period), even if the output of the early-learning area is reset on average, noise in the early-learning weight $w_{H}$—for example, resulting from noise in the pathway that carries instructive signals during learning—is integrated into changes in v (Fig. 3*A*–*D* and see *SI Appendix*, section S1, for analytical derivation of how the noise accumulates). The amplitude of changes in v during the post-training period, resulting from changes in $w_{H}$, defines the slope of the flow field (Fig. 3 *A* and *B*, gray arrows), which is proportional to the learning rate of v and inversely proportional to the rate at which the early-learning site $w_{H}$ is reset to baseline. This implies that a system with fast consolidation will reach a given level of consolidated learning quickly (i.e., in a small number of training sessions) but accumulate noise quickly as well. By contrast, a system with slow learning will require many training sessions to reach the same level of consolidated learning, but will reach this level with a smaller total level of consolidated noise (Fig. 3*E*–*H*). This represents a type of “speed-accuracy” tradeoff in achieving a given level of consolidated learning, similar to that seen in integration-based tasks like accumulation of evidence for decision-making (56, 57).

### Consolidation Mitigates the Stability–Plasticity Dilemma through Averaging.

**Fig. 4.**
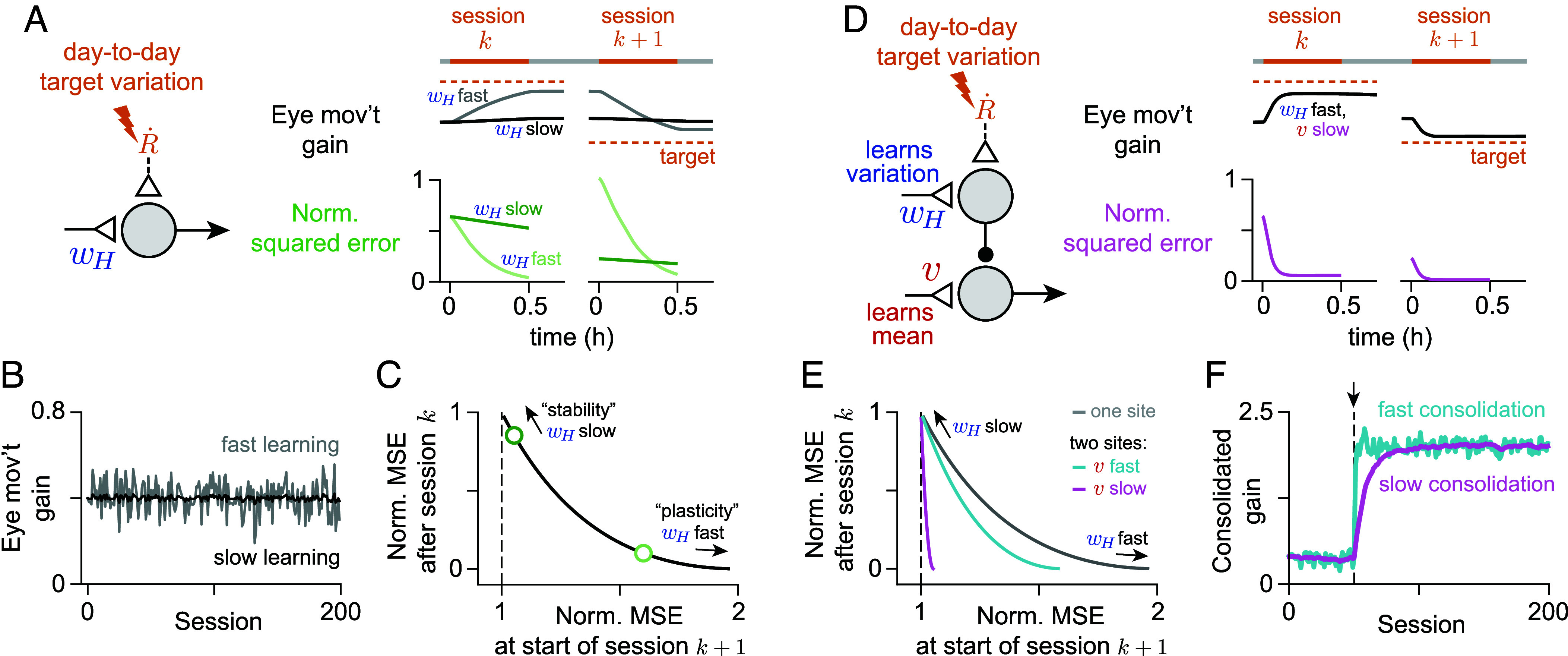
Consolidation averages over variability in the instructive signal to mitigate the stability-plasticity dilemma. (*A*) Learning in a model of a circuit with one site of plasticity that performs a graded amplitude input-to-output transformation is subject to the stability-plasticity dilemma. During a training session, the instructive signal drives a change in the weight $w_{H}$, which is stably remembered after training. Across training sessions, the externally instructed target value of the input-to-output gain varies randomly about a mean value. Fast learning at $w_{H}$ (light colors) reduces error more effectively during a training session compared to slow (dark colors), but increases the size of the expected error at the beginning of the next training session (distance from the orange dashed line). (*B*) Input-to-output gain over 200 training sessions in a simplified model that simulates weight changes discretely over sessions (*SI Appendix*, section S2). Slower learning (black) results in a more stable value of the weight due to greater averaging over variability in the instructive signal. (*C*) Expected (mean) squared error (MSE) after a training session in the simplified model trades off with expected error at the start of the subsequent session, calculated analytically and normalized to the variance of the target gain distribution. Dark and light green circles correspond to the learning rates used to generate the black and gray curves in panel (*B*). (*D*) A circuit with two sites of learning can mitigate the tradeoff in panel (*C*). A persistent, slow-learning site of consolidation, v, can estimate the mean of the expected gain distribution, while a forgetful, fast-learning early site, $w_{H}$, can account for day-to-day variation in the instructive signal. (*E*) Relationship between normalized expected MSE at the end versus start of a training session when there are two sites of learning with fast (cyan) or slow (magenta) learning at the late site v. Gray curve replots the relationship when there is only one site of learning (same as *C*). (*F*) Consolidated gain in response to a change in the mean of the target gain distribution (at session indicated with arrow) for relatively fast (cyan) or slow (magenta) learning at v (rates same as (*D*), but using the simplified model). Slow consolidation leads to reduced variability in the long run, but at the cost of larger errors early in training.

Framing consolidation as integration highlights mechanistically how systems consolidation can address the stability-plasticity dilemma (8, 9), i.e., the tradeoff between the ability of neural systems to adapt quickly and to not overwrite previous learning. Within the context of error-driven learning, this tradeoff arises when the optimal, “target” input-to-output gain of the circuit transformation, as conveyed by the instructive signals guiding learning, fluctuates across training sessions due to changes in the environment or in the motor plant (e.g., due to experimental manipulations, fatigue, injury, or changes in body mass). With one site of learning, a more plastic circuit that adapts quickly to reduce error during a given training session will tend to have a bigger initial error at the start of the next training session (Fig. 4*A*–*C*, “$w_{H}$ fast”). On the other hand, a more stable circuit, whose gain adapts slowly and approximates the mean of the fluctuating learning target, will have, on average, smaller initial errors across training sessions, but will less fully reduce the error during a given training session (Fig. 4*A*–*C*, “$w_{H}$ slow”; *SI Appendix*, section S2).

With two sites of learning—a fast-learning and fast-forgetting early site of plasticity, and a slow-learning late site of consolidation—the circuit can mitigate this tradeoff. Slow consolidation at v stores the long-term average of the fluctuating target gain conveyed by the instructive signals across training sessions, while fast plasticity at $w_{H}$ adapts quickly during a single training session but is reset after training (Fig. 4*D*). In this way, the circuit can both minimize the average error at the start of each training session and respond quickly to reduce errors within a session (Fig. 4*E* and *SI Appendix*, section S2). However, even with two sites of learning, the minimization is not perfect, and there is a tradeoff in the size of the expected error at the start of the next training session versus the number of sessions required to consolidate the mean target gain (Fig. 4*F*). This is another form of the speed-accuracy tradeoff discussed in the previous section (Fig. 3).

### Implications for Plasticity Rules.

To support consolidation, the late-learning site must be able to stably accumulate persistent weight changes over time. This requires that the plasticity rule at the late-learning site support a continuum of stable weight values, so that the synapse can persistently hold any change in the weight. This is readily achieved by a heterosynaptic plasticity rule (Fig. 2*E*), because weight changes stop whenever the activity at the early-learning site returns to its baseline or, more generally, becomes uncorrelated with the direct pathway input (58).

**Fig. 5.**
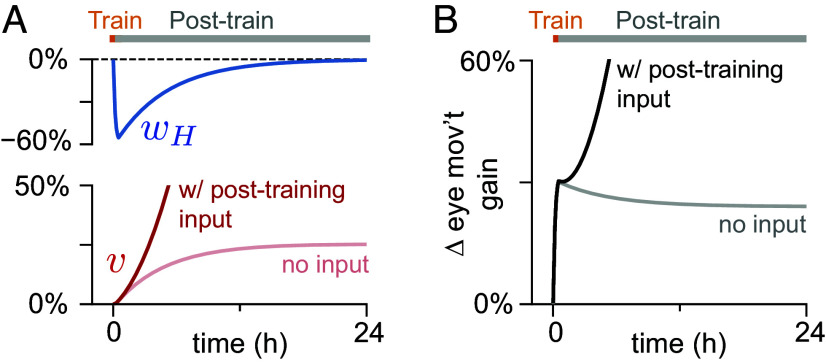
Hebbian covariance rule cannot support stable consolidation in the presence of time-varying signal or noise. (*A*) Evolution of the early- and late-learning weights, $w_{H}$ (blue) and v (red), over time when using a Hebbian covariance rule at v, during a simulation of 0.5 h of training to increase the input-to-output gain of the circuit, followed by a 23.5 h post-training period without information about errors, when time-varying input to the circuit was either present (dark lines) or not present (light “no input” lines) during the post-training period. (*B*) Change in the input-to-output gain corresponding to the simulations in (*A*).

Recent work has alternatively proposed a Hebbian, covariance-like rule for the consolidation of oculomotor learning (37). Here, we considered a Hebbian covariance-like rule of the form

$$\begin{array}{c} \Delta v\propto \;\text{MF}(\;\text{MVN}-\langle \;\text{MVN}\rangle ), \end{array}$$

in which plasticity is proportional to presynaptic activity (mossy fiber, MF) multiplied by the difference between postsynaptic activity (medial vestibular nucleus, MVN) and a sliding threshold equal to its recent average (angle brackets) (see *Learning rules* for the full equations). Our analysis shows that this covariance-like rule can support a continuum of stable values, and hence consolidation, if the circuit receives no time-varying input or noise during the post-training period (Fig. 5 *A* and *B*, light lines). However, in the more biologically realistic case that the circuit does receive time-varying input or noise, the synaptic weight v, and thus the input-to-output gain, becomes unstable, growing exponentially during the post-training period (Fig. 5 *A* and *B*, dark lines; *SI Appendix*, section S3).

The failure of the covariance-like rule reflects an inherent source of instability in Hebbian learning (59). In the basic Hebbian rule, correlations between presynaptic and postsynaptic firing rates drive increases in synaptic weights. These increased synaptic weights in turn drive increased postsynaptic activity and thus increased correlations between pre- and postsynaptic firing, forming a positive feedback loop. For the covariance-like rule considered above, the sliding threshold counters the increased correlations associated with changes in the average postsynaptic firing rate, but does not counter the increased correlations associated with fluctuations around the average (see *SI Appendix*, section S3; 60). Other proposed methods for countering this instability, including weight normalization (59, 61) and firing-rate-target homeostasis (62–65), also fail to support a continuum of stable values in our model. Rather, they yield dynamics that drive the late-learning weight to a single stable value (*SI Appendix*, section S4). Altogether, this analysis suggests that either heterosynaptic plasticity or a different variant of Hebbian rule from those typically considered is required to implement systems consolidation of an analog memory.

### A Circuit with an Internal Feedback Loop Requires an Active Post-Training Reset Mechanism at the Early-Learning Site.

In addition to constraints on the form of plasticity at the late-learning site, consolidation also requires that the output of the early-learning area be reset during the post-training period, stopping the integration at the late-learning site. For the simple feedforward model described above, this condition is met by the intrinsic decay of the early-learning weight $w_{H}$ to zero, which represents a balance of excitatory and inhibitory input to the early-learning area.

**Fig. 6.**
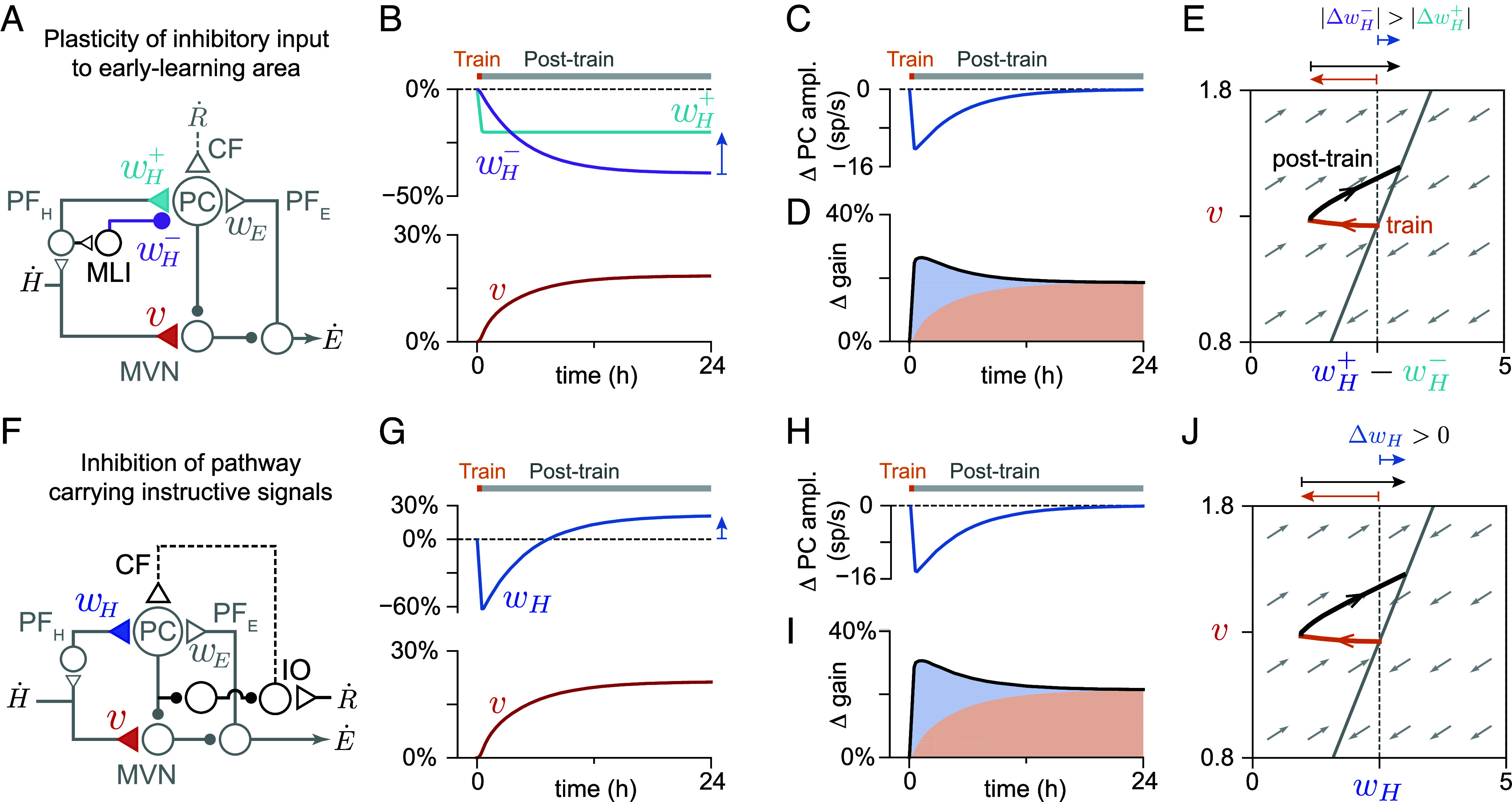
Circuit mechanisms for post-training reset enable consolidation in the presence of an internal feedback loop. (*A*–*E*) Reset of early-learning area output via plasticity of inhibitory input onto the early-learning area. (*A*) Circuit diagram. $\;{\text{PF}}_{H}$ and $\;{\text{PF}}_{E}$: cerebellar parallel fiber inputs to Purkinje cells (PC) carrying sensory (vestibular) input $\dot{H}$ and efference copy feedback of motor output $\dot{E}$. MLI: molecular layer interneuron. CF: climbing fiber. $\dot{R}$: information about behavioral (retinal slip) errors. (*B*) Change in early-learning excitatory weight $w_{H}^{+}$ (cyan), inhibitory weight $w_{H}^{-}$ (purple) and late-learning weight v (red), during 0.5 h of simulated training (orange block) to increase the input-to-output gain of the circuit, followed by a 23.5 h post-training period with no information about errors (gray block). At steady state, the value of $w_{H}^{-}$ has decreased more than $w_{H}^{+}$, so that the net change in the net weight $w_{H}=w_{H}^{+}-w_{H}^{-}$ is positive (blue arrow). (*C*) Early-learning area output (amplitude of Purkinje cell activity relative to moving baseline), which drives consolidation at the late-site. (*D*) Change in the gain of the eye movement response (black line) to a sensory input. Blue and red shaded areas show the contribution of the early- and late-learning areas to the circuit transformation. (*E*) Trajectory of synaptic weights during the training (orange) and post-training (black) periods. During training, the net early-learning weight decreases (orange arrow above plot) from its initial value (dashed black line), but after training (black arrow) approaches a steady-state value that is larger than before training (blue arrow). Gray arrows show the approximate instantaneous direction in which a weight configuration at a given point in synaptic weight space will evolve during the post-training period, determined analytically. All trajectories tend toward a marginally stable point along a line (solid gray line). (*F*–*J*) Reset of early-learning area output via inhibition of the pathway carrying instructive signals to the early-learning site. (*F*) Circuit diagram. IO: inferior olive. (*G*) Change in early-learning weight $w_{H}$ (dark blue) and late-learning weight v (red). The post-training reset drives $w_{H}$ to a steady-state value larger than before training (blue arrow). (*H*–*J*) Same as (*C*–*E*), but for model shown in (*F*).

An active, rather than passive, post-training reset mechanism is necessary for stable consolidation in the more complex case where the input-to-output transformation computed by the circuit contains an internal feedback loop. We modeled this case by adding to the feedforward model an internal feedback connection from the late- to the early-learning area (Fig. 6 *A* and *F*, and *SI Appendix*, section S5). For oculomotor learning, the internal loop corresponds to an efference copy of the eye movement command (66–69). Here, we describe a model with a fixed internal feedback weight $w_{E}>0$; similar results are obtained if $w_{E}$ is plastic (*SI Appendix*, Fig. S3 and section S6). As in the feedforward model, the condition that plasticity at the late-learning site supports stable accumulation can be met by a heterosynaptic plasticity rule at v. However, the reset condition can no longer be met just by the passive decay of the weight $w_{H}$ of the feedforward pathway in the early-learning area to a fixed baseline, but rather requires that, during the post-training period, $w_{H}$ be reset to a value that depends on v. This is because, due to the internal feedback from the late- to the early-learning area, changes in v also drive altered activity of the early-learning area. These changes must be offset following training by changes in $w_{H}$ so the feedforward sensory input and the feedback from the late-learning area effectively cancel, eliminating the drive for plasticity at the late-learning site and enabling v to reach a new steady state.

### The Reset Requirement Suggests a Role for Specific Features of Cerebellar Circuit Architecture in Consolidation.

Active reset of the early-learning site could be achieved in at least two ways. First, the feedforward input to the early-learning area can be decomposed into the sum of a direct excitatory and disynaptic inhibitory pathway that have weights $w_{H}^{+}$ and $w_{H}^{-}$, respectively, controlled by separate plasticity mechanisms (Fig. 6*A*). We assume that changes in the excitatory weight are driven only by instructive signals occurring during the training period, with no passive decay. We then find that, during the post-training period, plasticity in the inhibitory weight can reset the activity of the early-learning area such that it no longer responds to sensory input. This resetting is achieved when the inhibitory input weight is governed by a Hebbian rule driven by correlations between presynaptic inhibitory inputs and postsynaptic spiking relative to a sliding threshold (Fig. 6*B*–*E*; *Circuit Model with Internal Feedback Loop*; 70). Alternatively, the reset could be driven by inhibition from the late-learning area onto the source of the instructive signals that control plasticity at the early-learning site (Fig. 6*F*–*J*; *Circuit Model with Internal Feedback Loop*). In the cerebellar context, the first reset mechanism would correspond to plasticity of molecular layer interneuron-to-Purkinje cell synapses, and the second could be achieved by the inhibitory pathway from the cerebellar nuclei to the inferior olive (see *Discussion*). Thus, a central computational role for these components of the cerebellar circuitry may be to implement the post-training reset of the rapid, early plasticity in the cerebellar cortex to support stable consolidation of learning.

Our model predicts that, following consolidation of learning to increase the input-to-output gain of the circuit, the sensitivity of cells in the early-learning area to feedforward input, $w_{H}$, will be higher than the pre-training baseline, as has been suggested based on experimental measurements (68, 71). This can be understood more clearly by plotting the dynamics of the synaptic weights. During consolidation, the post-training reset dynamics cause the weights to move along a trajectory to a new steady state along a line in the space of synaptic weights. In the feedforward model, this line is vertical, so the early-learning weight returns to its pre-training baseline during the post-training consolidation period (Fig. 2*E*). When there is an internal feedback loop, the line has a finite, positive slope, so the net weight $w_{H}$ does not just return to its pre-training baseline value, but goes to a steady-state value that is larger than before training (Fig. 6 *E* and *J*, black arrows). Thus, our model predicts that, after consolidation, the change in the early-learning weight, relative to before training, will be in the opposite direction from the change in weight during training (Fig. 6 *E* and *J*, blue versus orange arrows above plot; 72).

## Discussion

We propose a computational principle governing systems consolidation of analog memories: Systems consolidation is defined by a temporal integration process in which the late-learning weight stably accumulates changes induced during training at the early-learning site. To obey this principle, the circuit must have certain properties: First, the synaptic weight at the late-learning site must be governed by plasticity rules that enable it to stably accumulate and maintain any one of a continuum of values. We show that this is achieved by a heterosynaptic plasticity rule but not by standard stabilized Hebbian rules. Second, the output of the early-learning area must be reset during the post-training period to stop the accumulation of weight changes at the late-learning site.

The accumulation of synaptic weight changes underlying systems consolidation is reminiscent of the temporal integration of transient spiking activity into persistent spiking activity in neural circuits that accumulate and store information in working memory, such as that implicated in the accumulation and storage of neural signals encoding evidence in decision-making tasks (1, 12–15, 17, 57, 73–75). Our model shares distinctive features with such neural integrator circuits, arising from their similar underlying dynamics. First, it is well known that integrator circuits require a fine-tuning of parameters for stability of the memory (1, 13, 14, 16). This fine-tuning occurs in two places: 1) in the strength of the recurrent connections between neurons, which offsets intrinsic decay of neural activity; and 2) in the strength of tonic background inputs entering the network. In the case of systems consolidation, we assume perfect integration (no decay) at the late-learning site, so only the second type of tuning is required. Specifically, output from the early-learning area must be reset during the post-training period; otherwise, it will drive incessant weight changes at the late-learning site. This is analogous to the finding that neural integrator circuits will integrate tonic background spiking activity unless these inputs are subtracted out or otherwise nullified (14, 16, 76). Drawing on the neural integrator literature, one proposed solution to this fine-tuning problem is to turn the continuum of steady states into a set of (many) finely discretized, robustly stable fixed points (77, 78). This could correspond to our single “lumped” synaptic weight v being composed of the sum of the binary weights of individual bistable synapses (79) that are recruited with different thresholds (77, 80). Second, noisy input causes diffusive drift that progressively corrupts the stored memory (Fig. 3; 53, 54, 55). In our model, this led to a tradeoff between the speed of consolidation and the accuracy of the consolidated memory (Fig. 3 and *SI Appendix*, section S1). This can be interpreted as the long-term memory analog of the speed-accuracy tradeoff in decision-making tasks (56, 57).

The similarities in dynamics between neural integrator circuits and our systems consolidation model arise from the fact that, in both cases, the memory stored in the circuit is analog—in our case, the amplitude of the circuit’s learned response to a given input is graded. Much of the previous theoretical work on systems consolidation has instead considered the case of effectively binary neurons (11, 29, 81–84), in which memory expression is evaluated with respect to whether or not a given neuron fires, rather than the graded value of its firing rate. In such models, Hebbian plasticity typically drives synaptic weights to take either a high (saturated) or a low value, making individual synaptic dynamics effectively bistable (59, 85). By contrast, in our model, the synaptic weight at the late-learning site must be able to stably take any analog value within a continuum, which is naturally achieved by a heterosynaptic plasticity rule. Although such heterosynaptic rules are commonly used in modeling supervised learning tasks (86), they may form a more specialized class of biological plasticity than Hebbian learning rules. Supporting the continuum of steady states required for consolidation is more challenging for classical Hebbian rules (*SI Appendix*, section S4), though may be possible with more complex forms of Hebbian plasticity, such as a three-factor (87) or dendritic plasticity rule (88). Alternatively, as noted in the previous paragraph, it is possible that the apparent continuum of weight values modeled here instead reflects a sum of discrete, bistable synaptic contributions.

The framing of systems consolidation as a process of temporal integration provides mechanistic insight into previous work on how consolidation addresses the stability-plasticity dilemma (8, 9). In our model, the stability-plasticity dilemma is mitigated because, in the presence of variability in the instructive signals about behavioral errors, slow integration (i.e., averaging) at the late-learning site tracks long-timescale behavioral requirements, while the early-learning site quickly tracks short-timescale changes in these requirements (Fig. 4*D*; for a related use of integration to improve deep network training, see ref. 89). In this manner, averaging leads to a more general memory at the late-learning site that is constantly updated by specific but transient memories at the early-learning site (20, 90–93). This framing could also be extended to describe synaptic consolidation, through which transient early plasticity is consolidated into persistent, graded late plasticity in the same synapse (94–96). The timescale over which the late-learning site averages is defined by the relative rates of plasticity at the late- and early-learning sites (Fig. 4*F*). This may suggest that the rate at which the circuit consolidates (i.e., the fraction of learning consolidated following training) is tuned so that the timescale of the average is matched to the timescale over which the mean is expected to change in the world (97).

Though systems consolidation appears to be a common feature of learning and memory systems, the details of how it occurs in each system may be shaped by specific computational needs. In hippocampus-dependent memory, the early-learning circuit in the hippocampus learns the associations between components or features of an episode. Post-training replay of the activity patterns representing these associations then drives consolidation to the neocortex (4, 11, 81, 83, 84, 98, 99). In the system we model, the circuit learns associations between a sensory input and a behavioral error signal that drives the system towards a desired output. Post-training “replay” of the activity patterns learned at the early site then drives consolidation of the learned input-to-output transformation at the late-learning site. Although in both cases the correlations present during training are recapitulated in the post-training neural activity, hippocampal replay occurs as discrete events with transient, spontaneous reactivation of representations (e.g., during a sharp wave ripple), whereas in the cerebellum the “replay” could be driven by ongoing input to the circuit.

Our work extends previous modeling of the consolidation of oculomotor learning (31–34, 36, 37) in two key ways. First, we analyzed the conditions under which consolidation can occur successfully. We showed that consolidation requires that plasticity at the late-learning site support a continuum of steady-state weight values, a requirement readily met by a heterosynaptic plasticity rule. This explains why heterosynaptic rules have been successful in previous work (32–34, 36), and is consistent with experimental work suggesting that plasticity at the late-learning site for cerebellum-dependent learning may be heterosynaptic (35, 100). Second, we considered an internal feedback loop from the late- to the early-learning area of the circuit, in contrast to the feedforward architecture used in previous models. This internal feedback loop, which carries an efference copy of motor commands, has been suggested to be essential for producing the dynamics of individual eye movement responses (67, 68). We showed that this internal feedback loop also fundamentally changes the dynamics of memory consolidation, with stable consolidation requiring a circuit mechanism through which plasticity resets the activity of the early-learning area following training (Fig. 6). This post-training reset provides a possible explanation for the previous suggestion, based on in vivo recordings (66, 68), that the weights of the parallel fiber input pathway to Purkinje cells may paradoxically undergo potentiation in cases where the standard hypothesis of error-driven cerebellar cortical plasticity predicts that this pathway undergoes depression. Our work suggests one possible resolution to this controversy: The initial synaptic changes are governed by depression (41, 49), but during consolidation the post-training reset mechanism reverses the decrease in weight, driving the weight to a larger steady-state value relative to before training (Fig. 6 *E* and *J*; see ref. 72 for further discussion as well as an alternative solution). Third, we propose two biologically plausible circuit mechanisms that can perform this reset, suggesting functional roles for elements of the cerebellar microcircuit in stabilizing a memory. The first mechanism, plasticity of the inhibitory feedforward inputs to the early-learning area, could be implemented by a Hebbian rule at the molecular layer interneuron (MLI)-to-Purkinje cell synapses (70). Although these synapses are known to be plastic (101, 102), weight changes were believed to be governed by climbing fiber-driven complex spikes in the postsynaptic Purkinje cell. Our model predicts that weight changes may instead be driven by the correlation of presynaptic MLI activity with Purkinje cell simple spikes. The second mechanism, inhibition of instructive signals by the late-learning area, could be implemented by an inhibitory pathway from the vestibular and cerebellar nuclei targeted by Purkinje cells to the inferior olive ( 103, 104; see refs. 33 and 38 for related ideas), and might explain nonvisual climbing fiber responses that have been observed in the oculomotor cerebellum (105, 106).

Learning and memory systems enable an organism to transform transient experiences into persistent effects. In working memory, neural integrator circuits accumulate and store transient input signals as persistent spiking activity. Here, we extend the concept of neural integration to the context of long-term memory by framing systems consolidation as a temporal integration process. Thus, this work suggests a unifying conceptual framework for describing computations underlying short- and long-term memory function.

## Materials and Methods

### Feedforward Sensorimotor Circuit Model.

Here we describe the feedforward model of Figs. 2–4. The circuit model has parallel pathways that transform a graded, time-varying input into a response. In particular, we modeled the transformation of a graded, time-varying sensory (vestibular) input into an eye movement response and its modification by cerebellum-dependent learning. We modeled each node in the circuit with a single variable representing the average firing rate of a population of cells: mossy fibers, MF(t); parallel fibers (granule cell axons), PF(t); Purkinje cells, PC(t); medial vestibular nucleus neurons, MVN(t); and climbing fibers, CF(t) (Fig. 2*A*). Specific values of model parameters used in simulations were taken from the literature, where available, and are summarized in *SI Appendix*, Table S1. For analysis, unless otherwise stated, all parameters are assumed to be nonnegative.

The firing rate of mossy fibers was modeled as a combination of a spontaneous baseline and a time-varying component encoding a sensory (vestibular) input driven by head motion $\dot{H}\left(t\right)$,

[1] $$\begin{array}{c} \;\text{MF}(t)=\;{\text{MF}}_{0}+\delta \;\text{MF}(t)=\;{\text{MF}}_{0}+k_{\text{MF}}\dot{H}(t), \end{array}$$

where we considered the encoding of the sensory input to be linear with sensitivity to head velocity *k*_MF_ (108). Rather than explicitly model the mossy fiber to granule cell transformation, we considered the firing rate of parallel fibers to similarly depend on vestibular input linearly,

[2] $$\begin{array}{c} \;\text{PF}(t)=\;{\text{PF}}_{0}+\delta \text{​}\text{PF}(t)=\;{\text{PF}}_{0}+k_{\text{PF}}\dot{H}(t), \end{array}$$

where *k*_PF_ was the firing rate sensitivity to head velocity.

Purkinje cells, in the early-learning area, were modeled as linearly combining direct excitatory input from parallel fibers and indirect inhibitory input via a parallel fiber-molecular layer interneuron pathway (37),

[3] $$\begin{array}{c} \;\text{PC}\left(t\right)=\;{\text{PC}}_{0}+\left(w_{H}^{+}\left(t\right)-w_{H}^{-}\right)\;\text{PF}\left(t\right)=\;{\text{PC}}_{0}+w_{H}\left(t\right)\;\text{PF}\left(t\right), \end{array}$$

where $w_{H}^{+}$ and $w_{H}^{-}$ are the total excitatory and inhibitory weights onto the Purkinje cell, so the early-learning weight $w_{H}=w_{H}^{+}-w_{H}^{-}$ is the net weight of the parallel fiber pathway onto Purkinje cells. For simplicity, the inhibitory weight was not plastic in the model; the dynamics of learning are driven by the net weight. Equivalently, $w_{H}\left(t\right)$ can be interpreted simply as modeling the dynamics of the net (excitatory minus inhibitory) weight onto Purkinje cells. The effect of climbing fibers, which fire at very low rates (∼1 sp/s), on Purkinje cell output was not modeled here.

The medial vestibular nucleus, the late-learning area, was modeled as responding linearly to its excitatory mossy fiber input and inhibitory Purkinje cell input (109, 110),

[4] $$\begin{array}{cc} \;\text{MVN}(t) & =\;{\text{MVN}}_{0}+v(t)\;\text{MF}(t)-w_{\text{PC}}\text{PC}(t)\\ & =\;{\text{MVN}}_{0}-w_{\text{PC}}{\text{PC}}_{0}+v(t)\;\text{MF}(t)-w_{H}(t)w_{\text{PC}}\text{PF}(t), \end{array}$$

where v is the late-learning weight of the vestibular mossy fiber input to the medial vestibular nucleus, *w*_PC_ is the weight of the Purkinje cell input to the MVN, and $\;{\text{MVN}}_{0}$ is an offset chosen so that the spontaneous MVN firing rate matched experimental values (57 sp/s) (110). The initial value of v before training was chosen so that the gain in the dark was 0.4 (50, 111).

Eye movement output, $\dot{E}\left(t\right)$, was modeled as being proportional to the deviation of medial vestibular nucleus neuron firing from its baseline (32, 37),

[5] $$\begin{array}{cc} \dot{E}\left(t\right) & =-k_{E}\left(\;\text{MVN}\left(t\right)-{\left\langle \;\text{MVN}\left(t\right)\right\rangle }_{\tau_{f}}\right). \end{array}$$

The baseline, ${\left\langle \;\text{MVN}\left(t\right)\right\rangle }_{\tau_{f}}$, was calculated as an exponential (low-pass filtered) average over the recent past of MVN activity. For any time-varying quantity x(t), its exponential average ${\left\langle x\left(t\right)\right\rangle }_{\tau }$ over timescale τ is defined by

[6] $$\begin{array}{c} \tau \frac{\;\text{d}{\left\langle x\right\rangle }_{\tau }}{\;\text{d}t}=-{\left\langle x\right\rangle }_{\tau }+x\left(t\right). \end{array}$$

The timescale of the average $\tau_{f}=0.017$ h (1 min) is long relative to variations in the sensory input, but is much shorter than the timescales of plasticity (defined below), so that ${\left\langle w_{H}\left(t\right)\right\rangle }_{\tau_{f}}\approx w_{H}\left(t\right)$ and ${\left\langle v\left(t\right)\right\rangle }_{\tau_{f}}\approx v\left(t\right)$ (37). Throughout the paper, we assume that over the timescale $\tau_{f}$, the sensory input is zero on average,

[7] $$\begin{array}{c} {\left\langle \dot{H}\left(t\right)\right\rangle }_{\tau_{f}}\approx 0. \end{array}$$

In the specific case of the bidirectional vestibular input to the oculomotor circuit, this corresponds to the time average of the rightward and leftward head movements being approximately equal.

The input-to-output gain of the circuit, g, is defined as the ratio of eye velocity output to head velocity input,

[8] $$\begin{array}{c} g(t)=-\frac{\dot{E}(t)}{\dot{H}(t)}\approx k_{E}(k_{\text{MF}}v(t)-k_{\text{PF}}w_{\text{PC}}w_{H}(t)), \end{array}$$

where the negative sign is present because eye movements are oppositely directed from head movements to keep the image of the world stable.

#### Learning rules.

Plasticity at the early-learning site was driven by feedback about behavioral errors, carried by the climbing fibers. Climbing fiber firing was represented by the sum of a spontaneous baseline rate and a time-varying component representing oculomotor errors,

[9] $$\begin{array}{c} \;\text{CF}\left(t\right)=\;{\text{CF}}_{0}+\delta \;\text{CF}\left(t\right). \end{array}$$

The time-varying component δCF(t) encodes retinal slip, or a failure of eye movements to stabilize images on the retina, which occurs when the input-to-output gain of the circuit is incorrect. During training, the target output ${\dot{E}}^{\;\text{target}}$ of the circuit is a (negatively) scaled version of the vestibular input $\dot{H}\left(t\right)$ with gain $g^{\;\text{target}}$,

[10] $$\begin{array}{c} {\dot{E}}^{\;\text{target}}\left(t\right)=-g^{\;\text{target}}\dot{H}\left(t\right), \end{array}$$

so, using Eq. **8**, the retinal slip error is

[11] $$\begin{array}{c} \dot{R}\left(t\right)={\dot{E}}^{\;\text{target}}\left(t\right)-\dot{E}\left(t\right)=-\left(g^{\;\text{target}}-g\left(t\right)\right)\dot{H}\left(t\right). \end{array}$$

We model the time-varying component of the climbing fiber firing as

[12] $$\begin{array}{c} \delta \text{​}\text{CF}(t)=k_{\text{CF}\text{​}}\tanh(-\beta_{\;\text{light}}\dot{R}), \end{array}$$

which is a saturating function of retinal slip. We chose the magnitude of the saturation to be $k_{\text{CF}}=\;{\text{CF}}_{0}=1$ Hz, so that when retinal slip errors are large, climbing fiber firing saturates at a maximum of 2 sp/s and a minimum of 0 sp/s (106, 112). When retinal slip errors are relatively small (relative to $1/\beta_{\;\text{light}}$), δCF(t) is approximately linearly related to negative retinal slip. Thus, if the eye movements are too small, so that the gain of the response needs to be increased, the covariance between the climbing fiber and parallel fiber firing rates is positive.

Experimentally, coactivation of parallel fibers and climbing fibers causes associative long-term depression at the early-learning site, whereas activation of parallel fibers alone causes long-term potentiation (113–115). Therefore, plasticity at the early-learning site was modeled as

[13] $$\begin{array}{c} \tau_{w}\frac{\;\text{d}w_{H}}{\;\text{d}t}=-(w_{H}(t)+w_{H}^{-})+k_{\text{LTP}\;}{\langle \text{PF}(t)\rangle }_{\tau_{f,w}}-k_{\text{LTD}}{\langle \text{​}\text{PF}(t)\cdot \text{​}\text{CF}(t)\rangle }_{\tau_{f,w}}, \end{array}$$

where the first term on the right is a passive decay, or forgetting, in the value of $w_{H}^{+}$ (32, 37). We chose *k*_LTP_ and *k*_LTD_ so that the steady-state value of $w_{H}^{+}$ when not training was equal to $w_{H}^{-}$, as required for stable consolidation (see *SI Appendix*, section S1.1, for details). The time constant of plasticity, $\tau_{w}$, was chosen to be relatively fast (0.15 h = 9 min) during training (111) and slower (5 h) during the post-training period (21, 41). The timescale of the averages over inputs, $\tau_{f,w}$, was chosen to be the same as $\tau_{f}$.

We considered two candidate learning rules for the late-learning site: a heterosynaptic and a Hebbian covariance-like rule. Heterosynaptic plasticity was modeled as

[14] $$\begin{array}{c} \frac{\;\text{d}v}{\;\text{d}t}=-k_{v,\;\text{hetero}}{\left\langle \;\text{MF}\left(t\right)\left(\;\text{PC}\left(t\right)-\;{\text{PC}}_{0}\right)\right\rangle }_{\tau_{f,v}}, \end{array}$$

where an increase in v is driven by the anticorrelation between mossy fiber firing and fluctuations of Purkinje cell activity around baseline (32–36, 100). Hebbian plasticity was modeled as

[15] $$\frac{\;\text{d}v}{\;\text{d}t}=k_{v,\;\text{Hebb}}{\langle \text{MF}(t)(\;\text{MVN}(t)-\theta (t))\rangle }_{\tau_{f,v}},$$

[16] $$\tau_{s}\frac{\;\text{d}\theta }{\;\text{d}t}=-\theta +\;\text{MVN}(t),$$

where the synaptic dynamics consist of the “readout” weight v and an internal sliding threshold variable θ that calculates a leaky average of postsynaptic activity (37). The threshold is similar to those used in previous studies to counteract the runaway plasticity that would otherwise result from the inherent positive feedback driven by this kind of correlational rule (see *SI Appendix*, section S4; 59, 62, 116). For stability, the timescale of the sliding threshold was chosen to be fast relative to the rate of plasticity (see *SI Appendix*, section S3, for details; 117).

#### Simulation of oculomotor learning.

We simulated oculomotor experiments with 0.5 h of training to increase the gain of the eye movement response, followed by a 23.5 h post-training period. During training, vestibular input was sinusoidal,

[17] $$\begin{array}{c} \dot{H}\left(t\right)=v_{\;\text{peak}}\sin\left(2\pi \;ft\right), \end{array}$$

with peak velocity $v_{\;\text{peak}}=15\;\text{deg/s}$ and rotational frequency f=1 Hz, which is representative of experimental protocols (41, 50, 111, 117). During the post-training period, we set $\dot{R}\equiv 0$. This models the animal being placed in the dark after training to eliminate feedback about oculomotor errors (40, 42–44, 111). We considered both the case in which vestibular input was not present during the post-training period (as in ref. 37), as well as the case in which it was present (as in ref. 32). We also studied the robustness of consolidated memories to perturbations in the early-learning site by adding random values to the early-learning weight $w_{H}$ at regular intervals (Fig. 3). All simulations were performed in Python by integrating the differential equations for the learning rules with the Radau solver (implemented in solve_ivp in the scipy.integrate package). Time steps in the simulation had a base unit of hours. Further simulation details are given in *SI Appendix*, section S7, and model parameters are shown in *SI Appendix*, Table S1.

#### Mitigation of stability-plasticity dilemma with two sites of learning.

Here we outline some simple analyses we performed to understand how the stability-plasticity dilemma is addressed in the presence of noisy instructive signals driving learning in the circuit. We modeled learning across multiple training sessions where, during each session k, the target gain $g^{\;\text{target}}={\hat{g}}^{\left(k\right)}$ used to calculate retinal slip was randomly drawn from a normal distribution. Target gain values were drawn independently across training sessions. We built simplified versions of the model with one and two sites of plasticity, in which we ignore the exact time course of the weight dynamics and instead describe how the weights change discretely across training sessions (*SI Appendix*, section S2 and Fig. 4 *B* and *F*). Using these simplified models, we calculated the mean squared error expected at the start and end of a typical training session as functions of the learning rates in the circuit (*SI Appendix*, section S2.2 and Fig. 4 *C* and *E*). To build intuition for the difference between the one-site and two-site models more concretely, in Fig. 4 *A* and *D*, we also show the detailed time course of the gain and the corresponding squared error (retinal slip) of the full, unsimplified one- and two-site models for two subsequent example training sessions (see *SI Appendix*, section S2.2, for details).

### Circuit Model with Internal Feedback Loop.

Here we describe the expanded version of the circuit model that includes both a feedforward pathway carrying the sensory (vestibular) input to the early-learning area (Purkinje cells) as well as an internal feedback loop carrying the output from the late-learning area (MVN) back to the early-learning area (Fig. 6 *A* and *F*). In the context of oculomotor learning, this internal feedback loop represents an efference copy of the output motor command. This expanded model has the same architecture as the Lisberger–Sejnowski model of oculomotor learning (67), but here we model the dynamics of the granule cell pathways as instantaneous. We show two candidate circuit mechanisms by which stable consolidation can be achieved when internal feedback is present.

The vestibular stimulus is carried by mossy fibers, described as before by Eq. **1**, and by vestibular parallel fibers with firing rate

[18] $$\begin{array}{c} \;{\text{PF}}_{H}\left(t\right)=\;{\text{PF}}_{0}+k_{\;\text{PF},H}\dot{H}\left(t\right). \end{array}$$

The efference copy is carried through a different set of parallel fibers with firing rate

[19] $$\begin{array}{c} \;{\text{PF}}_{E}\left(t\right)=\;{\text{PF}}_{0}+k_{\;\text{PF},E}\dot{E}\left(t\right), \end{array}$$

where the eye velocity output, $\dot{E}\left(t\right)$, is defined as before in Eq. **5**. The firing rate of Purkinje cells is then a linear combination of vestibular and efference copy input, with a spontaneous baseline rate, i.e.,

[20] $$\begin{array}{c} \;\text{PC}\left(t\right)=\;{\text{PC}}_{0}+w_{H}\left(t\right)\;{\text{PF}}_{H}\left(t\right)+w_{E}\left(t\right)\;{\text{PF}}_{E}\left(t\right). \end{array}$$

We assume that both parallel fiber pathways result in monosynaptic excitation and disynaptic inhibition onto the Purkinje cell, so that

[21] $$\begin{array}{cc} w_{H}\left(t\right) & =w_{H}^{+}\left(t\right)-w_{H}^{-}, \end{array}$$

[22] $$\begin{array}{cc} w_{E}\left(t\right) & =w_{E}^{+}\left(t\right)-w_{E}^{-}, \end{array}$$

where $w_{H}^{+}$ and $w_{E}^{+}$ are the excitatory weights of the feedforward and feedback parallel fibers, and $w_{H}^{-}$ and $w_{E}^{-}$ are the inhibitory weights of the interneurons in each pathway. Thus, as before, we can interpret $w_{H}=0$ and $w_{E}=0$ as balanced excitatory and inhibitory weights for each pathway. The firing rate of MVN neurons is defined as before in the first line of Eq. **4**, except we substitute the new definition of Purkinje cell activity from Eq. **20**. The late-learning weight of the direct pathway is still called v. Then, following the same reasoning as the derivation leading to Eq. **8**, we can calculate the eye velocity output as

[23] $$\begin{array}{c} \dot{E}(t)\approx -\frac{k_{E}(\tilde{v}(t)-w_{\text{PC}}{\tilde{w}}_{H}(t))}{1-w_{\text{PC}}k_{E}{\tilde{w}}_{E}(t)}\dot{H}(t)=-g(t)\dot{H}(t), \end{array}$$

defining the prefactor in the second equality as the gain g(t), and where for simplicity we let

[24] $$\begin{array}{cc} \tilde{v}(t) & =k_{\text{MF}}\cdot v(t), \end{array}$$

[25] $$\begin{array}{cc} {\tilde{w}}_{H}\left(t\right) & =k_{\;\text{PF},H}\cdot w_{H}\left(t\right), \end{array}$$

[26] $$\begin{array}{cc} \;\text{and}\;{\tilde{w}}_{E}\left(t\right) & =k_{\;\text{PF},E}\cdot w_{E}\left(t\right). \end{array}$$

We use modified learning rules for the excitatory weights in the parallel fiber pathways, in which the weight values do not passively decay,

[27] $$\begin{array}{c} \begin{array}{cc} \frac{\;\text{d}w_{x}^{+}}{\;\text{d}t} & =k_{\text{LTP}}{\langle \text{​}{\text{PF}}_{x}(t)\rangle }_{\tau_{f,w}}-k_{\text{LTD}}{\langle \text{​}{\text{PF}}_{x}(t)\cdot \text{​}\text{CF}(t)\rangle }_{\tau_{f,w}}\\ & \approx w_{x,\infty }^{+}-c_{x}(t), \end{array} \end{array}$$

where x=H or x=E for the head pathway or efference copy pathway respectively, and $w_{H,\infty }^{+}=w_{E,\infty }^{+}=\;{\text{PF}}_{0}(k_{\text{LTP}}-k_{\text{LTD}}{\text{CF}}_{0})$ and $c_{x}(t)=k_{\text{LTD}}{\langle \delta \text{​}{\text{PF}}_{x}(t)\delta \text{​}\text{CF}(t)\rangle }_{\tau_{f,w}}$. Here, we assumed that $w_{E}$ has a fixed nonnegative value for the purpose of visualizing the dynamics, but stable consolidation was also possible when $w_{E}$ was plastic (*SI Appendix*, Fig. S3 and section S6). So that plasticity is completely controlled by the correlation between parallel fibers and climbing fibers, we assumed values of *k*_LTD_ and *k*_LTP_ such that $w_{H,\infty }^{+}=0$. This condition can be interpreted as the plasticity due to spontaneous PF activity being offset by spontaneous CF activity (38).

For the direct pathway weight v, we used a modified version of the heterosynaptic rule in Eq. **14**,

[28] $$\begin{array}{c} \frac{\;\text{d}v}{\;\text{d}t}=-k_{v,\;\text{hetero}}{\left\langle \;\text{MF}\left(t\right)\left(\;\text{PC}\left(t\right)-{\left\langle \;\text{PC}\left(t\right)\right\rangle }_{\tau_{f}}\right)\right\rangle }_{\tau_{f,v}}, \end{array}$$

where the subtraction of the fixed Purkinje cell baseline has been replaced with a sliding threshold equal to the average PC activity over the recent past, similar to that used in the covariance-like Hebb rule, Eq. **15**. The time constant of the sliding threshold was $\tau_{f}$, which was slow relative to variations in the sensory input but fast compared to the rates of plasticity.

As discussed in the main text, stable consolidation in the presence of a feedback loop requires that the circuit implement an active mechanism to reset early-learning activity during the post-training period. We considered two such mechanisms. First, stable consolidation can be achieved if the feedforward inhibitory synapses onto the early-learning area ($w_{H}^{-}$) are plastic (Fig. 6*A*), governed by a Hebbian covariance-like rule,

[29] $$\begin{array}{c} \frac{\;\text{d}w_{H}^{-}}{\;\text{d}t}=k_{\;\text{inh}}{\left\langle \;{\text{PF}}_{H}\left(t\right)\left(\;\text{PC}\left(t\right)-{\left\langle \;\text{PC}\left(t\right)\right\rangle }_{\tau_{f}}\right)\right\rangle }_{\tau_{\;\text{inh}}}. \end{array}$$

Second, stable consolidation can be achieved if, in addition to providing information about behavioral errors, the climbing fiber input at the early-learning site also serves to reset early-learning activity (Fig. 6*F*). We modeled climbing fiber activity as being driven by the instructive signal and inhibited by medial vestibular nucleus cells that receive only Purkinje cell inhibition, which corresponds to net excitation by PC activity,

[30] $$\begin{array}{c} \delta \text{​}\text{CF}(t)=k_{\text{CF}}\tanh\left(-\beta_{\text{​}\text{light}}\dot{R}(t)+\beta_{\text{reset}}\delta \text{PC}(t)\right), \end{array}$$

where $\delta \;\text{PC}\left(t\right)=\;\text{PC}\left(t\right)-{\left\langle \;\text{PC}\left(t\right)\right\rangle }_{\tau_{f}}$. For both mechanisms, stable consolidation requires that the effective rate of plasticity of v be slower than that of $w_{H}$ (*SI Appendix*, section S5).

We simulated the same oculomotor experiment as for the model without feedback (see *Simulation of oculomotor learning* and *SI Appendix*, section S7), with differences in parameters specified in *SI Appendix*, Tables S2 and S3.

## Supplementary Material

Appendix 01 (PDF)

## Data Availability

Code for simulations, analyses, and producing plots for figures can be found on GitHub (118).

## References

[1] J. Zylberberg, B. W. Strowbridge, Mechanisms of persistent activity in cortical circuits: Possible neural substrates for working memory. Annu. Rev. Neurosci. **40**, 603–627 (2017). 28772102 10.1146/annurev-neuro-070815-014006 PMC5995341

[2] P. Goldman-Rakic, Cellular basis of working memory. Neuron **14**, 477–485 (1995). 7695894 10.1016/0896-6273(95)90304-6

[3] Y. Dudai, A. Karni, J. Born, The consolidation and transformation of memory. Neuron **88**, 20–32 (2015). 26447570 10.1016/j.neuron.2015.09.004

[4] L. R. Squire, L. Genzel, J. T. Wixted, R. G. Morris, Memory consolidation. Cold Spring Harb. Perspect. Biol. **7**, a021766 (2015). 26238360 10.1101/cshperspect.a021766 PMC4526749

[5] L. Genzel, J. T. Wixted, “Cellular and systems consolidation of declarative memory” in *Cognitive Neuroscience of Memory Consolidation*, N. Axmacher, B. Rasch, Eds. (Springer International Publishing, Cham, 2017), pp. 3–16.

[6] F. H. Do Monte, G. J. Quirk, B. Li, M. A. Penzo, F. H. Do Monte, G. J. Quirk, B. Li, M. A. Penzo, Retrieving fear memories, as time goes by... *Mol. Psychiatry* **21**, 1027–1036 (2016). 10.1038/mp.2016.78 PMC4956525 27217148

[7] J. W. Krakauer, R. Shadmehr, Consolidation of motor memory. Trends Neurosci. **29**, 58–64 (2006). 16290273 10.1016/j.tins.2005.10.003 PMC2553888

[8] W. C. Abraham, A. Robins, Memory retention–the synaptic stability versus plasticity dilemma. Trends Neurosci. **28**, 73–78 (2005). 15667929 10.1016/j.tins.2004.12.003

[9] S. Grossberg, Competitive learning: From interactive activation to adaptive resonance. Cogn. Sci. **11**, 23–63 (1987).

[10] J. L. McClelland, B. L. McNaughton, R. C. O’Reilly, Why there are complementary learning systems in the hippocampus and neocortex: Insights from the successes and failures of connectionist models of learning and memory. Psychol. Rev. **102**, 419–457 (1995). 7624455 10.1037/0033-295X.102.3.419

[11] A. Roxin, S. Fusi, Efficient partitioning of memory systems and its importance for memory consolidation. PLoS Comput. Biol. **9**, e1003146 (2013). 23935470 10.1371/journal.pcbi.1003146 PMC3723499

[12] C. D. Brody, R. Romo, A. Kepecs, Basic mechanisms for graded persistent activity: Discrete attractors, continuous attractors, and dynamic representations. Curr. Opin. Neurobiol. **13**, 204–211 (2003). 12744975 10.1016/s0959-4388(03)00050-3

[13] R. Chaudhuri, I. Fiete, Computational principles of memory. Nat. Neurosci. **19**, 394–403 (2016). 26906506 10.1038/nn.4237

[14] M. S. Goldman, A. Compte, X. J. Wang, Neural Integrator Models in Encyclopedia of Neuroscience (Elsevier, 2009), pp. 165–178.

[15] G. Major, D. Tank, Persistent neural activity: Prevalence and mechanisms. Curr. Opin. Neurobiol. **14**, 675–684 (2004). 15582368 10.1016/j.conb.2004.10.017

[16] H. S. Seung, How the brain keeps the eyes still. Proc. Natl. Acad. Sci. U.S.A. **93**, 13339–13344 (1996). 8917592 10.1073/pnas.93.23.13339 PMC24094

[17] X. J. Wang, Synaptic reverberation underlying mnemonic persistent activity. Trends Neurosci. **24**, 455–463 (2001). 11476885 10.1016/s0166-2236(00)01868-3

[18] O. Hardt, K. Nader, L. Nadel, Decay happens: The role of active forgetting in memory. Trends Cogn. Sci. **17**, 111–120 (2013). 23369831 10.1016/j.tics.2013.01.001

[19] E. Lesburguères , Early tagging of cortical networks is required for the formation of enduring associative memory. Science **331**, 924–928 (2011). 21330548 10.1126/science.1196164

[20] B. A. Richards, P. W. Frankland, The persistence and transience of memory. Neuron **94**, 1071–1084 (2017). 28641107 10.1016/j.neuron.2017.04.037

[21] S. F. Cooke, P. J. E. Attwell, C. H. Yeo, Temporal properties of cerebellar-dependent memory consolidation. J. Neurosci. **24**, 2934–2941 (2004). 15044532 10.1523/JNEUROSCI.5505-03.2004 PMC6729844

[22] S. G. Lisberger, The rules of cerebellar learning: Around the Ito hypothesis. Neuroscience **462**, 164–190 (2021). 10.1016/j.neuroscience.2020.08.026 PMC7914257 32866603

[23] J. L. Raymond, J. F. Medina, Computational principles of supervised learning in the cerebellum. Annu. Rev. Neurosci. **41**, 233–253 (2018). 29986160 10.1146/annurev-neuro-080317-061948 PMC6056176

[24] A. S. Andalman, M. S. Fee, A basal ganglia-forebrain circuit in the songbird biases motor output to avoid vocal errors. Proc. Natl. Acad. Sci. U.S.A. **106**, 12518–12523 (2009). 19597157 10.1073/pnas.0903214106 PMC2709669

[25] T. L. Warren, E. C. Tumer, J. D. Charlesworth, M. S. Brainard, Mechanisms and time course of vocal learning and consolidation in the adult songbird. J. Neurophysiol. **106**, 1806–1821 (2011). 21734110 10.1152/jn.00311.2011 PMC3191835

[26] H. H. Yin , Dynamic reorganization of striatal circuits during the acquisition and consolidation of a skill. Nat. Neurosci. **12**, 333–341 (2009). 19198605 10.1038/nn.2261 PMC2774785

[27] H. Makino, E. J. Hwang, N. G. Hedrick, T. Komiyama, Circuit mechanisms of sensorimotor learning. Neuron **92**, 705–721 (2016). 27883902 10.1016/j.neuron.2016.10.029 PMC5131723

[28] T. Teşileanu, B. Ölveczky, V. Balasubramanian, Rules and mechanisms for efficient two-stage learning in neural circuits. eLife **6**, e20944 (2017). 28374674 10.7554/eLife.20944 PMC5380437

[29] J. M. Murray, G. S. Escola, Remembrance of things practiced with fast and slow learning in cortical and subcortical pathways. Nat. Commun. **11**, 6441 (2020). 33361766 10.1038/s41467-020-19788-5 PMC7758336

[30] J. F. Medina, J. Christopher Repa, M. D. Mauk, J. E. LeDoux, Parallels between cerebellum- and amygdala-dependent conditioning. Nat. Rev. Neurosci. **3**, 122–131 (2002). 11836520 10.1038/nrn728

[31] L. An , Flexible learning models utilizing different neural plasticities. IEEE Trans. Cogn. Dev. Syst. **15**, 1150–1160 (2023).

[32] C. Clopath, A. Badura, C. I. De Zeeuw, N. Brunel, A cerebellar learning model of vestibulo-ocular reflex adaptation in wild-type and mutant mice. J. Neurosci. **34**, 7203–7215 (2014). 24849355 10.1523/JNEUROSCI.2791-13.2014 PMC6608186

[33] D. J. Herzfeld, N. J. Hall, M. Tringides, S. G. Lisberger, Principles of operation of a cerebellar learning circuit. eLife **9**, e55217 (2020). 32352914 10.7554/eLife.55217 PMC7255800

[34] J. F. Medina, M. D. Mauk, Simulations of cerebellar motor learning: Computational analysis of plasticity at the mossy fiber to deep nucleus synapse. J. Neurosci. **19**, 7140–7151 (1999). 10436067 10.1523/JNEUROSCI.19-16-07140.1999 PMC6782874

[35] J. R. W. Menzies, J. Porrill, M. Dutia, P. Dean, Synaptic plasticity in medial vestibular nucleus neurons: Comparison with computational requirements of VOR adaptation. PLoS One **5**, e13182 (2010). 20957149 10.1371/journal.pone.0013182 PMC2950150

[36] J. Porrill, P. Dean, Cerebellar motor learning: When is cortical plasticity not enough? PLoS Comput. Biol. **3**, e197 (2007). 17967048 10.1371/journal.pcbi.0030197 PMC2041974

[37] T. Yamazaki, S. Nagao, W. Lennon, S. Tanaka, Modeling memory consolidation during posttraining periods in cerebellovestibular learning. Proc. Natl. Acad. Sci. U.S.A. **112**, 3541–3546 (2015). 25737547 10.1073/pnas.1413798112 PMC4371920

[38] G. T. Kenyon, J. F. Medina, M. D. Mauk, A mathematical model of the cerebellar-olivary system I: Self-regulating equilibrium of climbing fiber activity. J. Comput. Neurosci. **5**, 17–33 (1998). 9540047 10.1023/a:1008874209991

[39] D. M. Broussard, C. D. Kassardjian, Learning in a simple motor system. Learn. Mem. **11**, 127–136 (2004). 15054127 10.1101/lm.65804

[40] M. Anzai, H. Kitazawa, S. Nagao, Effects of reversible pharmacological shutdown of cerebellar flocculus on the memory of long-term horizontal vestibulo-ocular reflex adaptation in monkeys. Neurosci. Res. **68**, 191–198 (2010). 20674618 10.1016/j.neures.2010.07.2038

[41] D. C. Jang, H. G. Shim, S. J. Kim, Intrinsic plasticity of cerebellar Purkinje cells contributes to motor memory consolidation. J. Neurosci. **40**, 4145–4157 (2020). 32295816 10.1523/JNEUROSCI.1651-19.2020 PMC7244189

[42] C. D. Kassardjian , The site of a motor memory shifts with consolidation. J. Neurosci. **25**, 7979–7985 (2005). 16135754 10.1523/JNEUROSCI.2215-05.2005 PMC6725450

[43] S. Nagao, H. Kitazawa, Effects of reversible shutdown of the monkey flocculus on the retention of adaptation of the horizontal vestibulo-ocular reflex. Neuroscience **118**, 563–570 (2003). 12699790 10.1016/s0306-4522(02)00991-0

[44] F. Shutoh, M. Ohki, H. Kitazawa, S. Itohara, S. Nagao, Memory trace of motor learning shifts transsynaptically from cerebellar cortex to nuclei for consolidation. Neuroscience **139**, 767–777 (2006). 16458438 10.1016/j.neuroscience.2005.12.035

[45] J. S. Albus, A theory of cerebellar function. Math. Biosci. **10**, 25–61 (1971).

[46] D. Marr, A theory of cerebellar cortex. J. Physiol. **202**, 437–470 (1969). 5784296 10.1113/jphysiol.1969.sp008820 PMC1351491

[47] M. Ito, Cerebellar control of the vestibulo-ocular reflex–around the flocculus hypothesis. Annu. Rev. Neurosci. **5**, 275–297 (1982). 6803651 10.1146/annurev.ne.05.030182.001423

[48] E. S. Boyden, A. Katoh, J. L. Raymond, Cerebellum-dependent learning: The role of multiple plasticity mechanisms. Annu. Rev. Neurosci. **27**, 581–609 (2004). 15217344 10.1146/annurev.neuro.27.070203.144238

[49] T. Inoshita, T. Hirano, Occurrence of long-term depression in the cerebellar flocculus during adaptation of optokinetic response. eLife **7**, e36209 (2018). 29582755 10.7554/eLife.36209 PMC5871328

[50] R. R. Kimpo, J. M. Rinaldi, C. K. Kim, H. L. Payne, J. L. Raymond, Gating of neural error signals during motor learning. eLife **3**, e02076 (2014). 24755290 10.7554/eLife.02076 PMC3989583

[51] J. G. McElligott, P. Beeton, J. Polk, Effect of cerebellar inactivation by lidocaine microdialysis on the vestibuloocular reflex in goldfish. J. Neurophysiol. **79**, 1286–1294 (1998). 9497410 10.1152/jn.1998.79.3.1286

[52] T. Okamoto, T. Shirao, F. Shutoh, T. Suzuki, S. Nagao, Post-training cerebellar cortical activity plays an important role for consolidation of memory of cerebellum-dependent motor learning. Neurosci. Lett. **504**, 53–56 (2011). 21911037 10.1016/j.neulet.2011.08.056

[53] Y. Burak, I. R. Fiete, Fundamental limits on persistent activity in networks of noisy neurons. Proc. Natl. Acad. Sci. U.S.A. **109**, 17645–17650 (2012). 23047704 10.1073/pnas.1117386109 PMC3491496

[54] S. Ganguli, D. Huh, H. Sompolinsky, Memory traces in dynamical systems. Proc. Natl. Acad. Sci. U.S.A. **105**, 18970–18975 (2008). 19020074 10.1073/pnas.0804451105 PMC2596211

[55] S. Lim, M. S. Goldman, Noise tolerance of attractor and feedforward memory models. Neural Comput. **24**, 332–390 (2012). 22091664 10.1162/NECO_a_00234 PMC5529185

[56] R. Bogacz, E. J. Wagenmakers, B. U. Forstmann, S. Nieuwenhuis, The neural basis of the speed–accuracy tradeoff. Trends Neurosci. **33**, 10–16 (2010). 19819033 10.1016/j.tins.2009.09.002

[57] J. I. Gold, M. N. Shadlen, The neural basis of decision making. Annu. Rev. Neurosci. **30**, 535–574 (2007). 17600525 10.1146/annurev.neuro.29.051605.113038

[58] P. Dean, J. Porrill, J. V. Stone, Decorrelation control by the cerebellum achieves oculomotor plant compensation in simulated vestibulo-ocular reflex. Proc. R. Soc. Lond. B Biol. Sci. **269**, 1895–1904 (2002). 10.1098/rspb.2002.2103 PMC1691115 12350251

[59] K. D. Miller, D. J. C. MacKay, The role of constraints in Hebbian learning. Neural Comput. **6**, 100–126 (1994).

[60] Y. Loewenstein, Robustness of learning that is based on covariance-driven synaptic plasticity. PLoS Comput. Biol. **4**, e1000007 (2008). 18369414 10.1371/journal.pcbi.1000007 PMC2265526

[61] E. Oja, Simplified neuron model as a principal component analyzer. J. Math. Biol. **15**, 267–273 (1982). 7153672 10.1007/BF00275687

[62] E. L. Bienenstock, L. N. Cooper, P. W. Munro, Theory for the development of neuron selectivity: Orientation specificity and binocular interaction in visual cortex. J. Neurosci. **2**, 32–48 (1982). 7054394 10.1523/JNEUROSCI.02-01-00032.1982 PMC6564292

[63] M. Chistiakova, N. M. Bannon, J. Y. Chen, M. Bazhenov, M. Volgushev, Homeostatic role of heterosynaptic plasticity: Models and experiments. Front. Comput. Neurosci. **9**, 89 (2015). 26217218 10.3389/fncom.2015.00089 PMC4500102

[64] G. G. Turrigiano, The self-tuning neuron: Synaptic scaling of excitatory synapses. Cell **135**, 422–435 (2008). 18984155 10.1016/j.cell.2008.10.008 PMC2834419

[65] P. Yger, M. Gilson, Models of metaplasticity: A review of concepts. Front. Comput. Neurosci. **9**, 138 (2015). 26617512 10.3389/fncom.2015.00138 PMC4639700

[66] F. A. Miles, S. G. Lisberger, Plasticity in the vestibulo-ocular reflex: A new hypothesis. Annu. Rev. Neurosci. **4**, 273–299 (1981). 6784658 10.1146/annurev.ne.04.030181.001421

[67] S. G. Lisberger, T. J. Sejnowski, Motor learning in a recurrent network model based on the vestibulo-ocular reflex. Nature **360**, 159–161 (1992). 1436091 10.1038/360159a0

[68] S. G. Lisberger, Neural basis for motor learning in the vestibuloocular reflex of primates. III. Computational and behavioral analysis of the sites of learning. J. Neurophysiol. **72**, 974–998 (1994). 7983549 10.1152/jn.1994.72.2.974

[69] A. L. Person, Corollary discharge signals in the cerebellum. Biol. Psychiatry Cogn. Neurosci. Neuroimaging **4**, 813–819 (2019). 31230918 10.1016/j.bpsc.2019.04.010 PMC6733673

[70] T. P. Vogels, H. Sprekeler, F. Zenke, C. Clopath, W. Gerstner, Inhibitory plasticity balances excitation and inhibition in sensory pathways and memory networks. Science **334**, 1569–1573 (2011). 22075724 10.1126/science.1211095

[71] F. A. Miles, D. J. Braitman, B. M. Dow, Long-term adaptive changes in primate vestibuloocular reflex. IV. Electrophysiological observations in flocculus of adapted monkeys. J. Neurophysiol. **43**, 1477–1493 (1980). 6768854 10.1152/jn.1980.43.5.1477

[72] H. L. Payne, J. L. Raymond, M. S. Goldman, Interactions between circuit architecture and plasticity in a closed-loop cerebellar system. eLife **13**, e84770 (2024). 38451856 10.7554/eLife.84770 PMC10919899

[73] A. K. Churchland, J. Ditterich, New advances in understanding decisions among multiple alternatives. Curr. Opin. Neurobiol. **22**, 920–926 (2012). 22554881 10.1016/j.conb.2012.04.009 PMC3422607

[74] M. Usher, J. L. McClelland, The time course of perceptual choice: The leaky, competing accumulator model. Psychol. Rev. **108**, 550–592 (2001). 11488378 10.1037/0033-295x.108.3.550

[75] X. J. Wang, Decision making in recurrent neuronal circuits. Neuron **60**, 215–234 (2008). 18957215 10.1016/j.neuron.2008.09.034 PMC2710297

[76] S. C. Cannon, D. A. Robinson, S. Shamma, A proposed neural network for the integrator of the oculomotor system. Biol. Cybern. **49**, 127–136 (1983). 6661444 10.1007/BF00320393

[77] M. S. Goldman, J. H. Levine, G. Major, D. W. Tank, H. S. Seung, Robust persistent neural activity in a model integrator with multiple hysteretic dendrites per neuron. Cereb. Cortex **13**, 1185–1195 (2003). 14576210 10.1093/cercor/bhg095

[78] A. A. Koulakov, S. Raghavachari, A. Kepecs, J. E. Lisman, Model for a robust neural integrator. Nat. Neurosci. **5**, 775–782 (2002). 12134153 10.1038/nn893

[79] D. H. O’Connor, G. M. Wittenberg, S. S. H. Wang, Graded bidirectional synaptic plasticity is composed of switch-like unitary events. Proc. Natl. Acad. Sci. U.S.A. **102**, 9679–9684 (2005). 15983385 10.1073/pnas.0502332102 PMC1172253

[80] M. Nikitchenko, A. Koulakov, Neural integrator: A sandpile model. Neural Comput. **20**, 2379–2417 (2008). 18533820 10.1162/neco.2008.12-06-416 PMC2814182

[81] P. Alvarez, L. R. Squire, Memory consolidation and the medial temporal lobe: A simple network model. Proc. Natl. Acad. Sci. U.S.A. **91**, 7041–7045 (1994). 8041742 10.1073/pnas.91.15.7041 PMC44334

[82] M. W. H. Remme , Hebbian plasticity in parallel synaptic pathways: A circuit mechanism for systems memory consolidation. PLoS Comput. Biol. **17**, e1009681 (2021). 34874938 10.1371/journal.pcbi.1009681 PMC8683039

[83] D. F. Tomé, S. Sadeh, C. Clopath, Coordinated hippocampal-thalamic-cortical communication crucial for engram dynamics underneath systems consolidation. Nat. Commun. **13**, 840 (2022). 35149680 10.1038/s41467-022-28339-z PMC8837777

[84] G. M. Wittenberg, M. R. Sullivan, J. Z. Tsien, Synaptic reentry reinforcement based network model for long-term memory consolidation. Hippocampus **12**, 637–647 (2002). 12440578 10.1002/hipo.10102

[85] D. W. Dong, J. J. Hopfield, Dynamic properties of neural networks with adapting synapses. Netw. Comput. Neural Syst. **3**, 267–283 (1992).

[86] P. Dayan, L. F. Abbott, Theoretical Neuroscience: Computational and Mathematical Modeling of Neural Systems, Computational Neuroscience (MIT Press, Cambridge, MA, 2005).

[87] Ł Kuśmierz, T. Isomura, T. Toyoizumi, Learning with three factors: Modulating Hebbian plasticity with errors. Curr. Opin. Neurobiol. **46**, 170–177 (2017). 28918313 10.1016/j.conb.2017.08.020

[88] R. Urbanczik, W. Senn, Learning by the dendritic prediction of somatic spiking. Neuron **81**, 521–528 (2014). 24507189 10.1016/j.neuron.2013.11.030

[89] R. Johnson, T. Zhang, “Accelerating stochastic gradient descent using predictive variance reduction” in *Advances in Neural Information Processing Systems*, C. J. Burges, L. Bottou, M. Welling, Z. Ghahramani, K. Q. Weinberge, Eds. (Curran Associates, Inc., 2013), vol. 26.

[90] M. J. Sekeres, G. Winocur, M. Moscovitch, The hippocampus and related neocortical structures in memory transformation. Neurosci. Lett. **680**, 39–53 (2018). 29733974 10.1016/j.neulet.2018.05.006

[91] D. Tse , Schemas and memory consolidation. Science **316**, 76–82 (2007). 17412951 10.1126/science.1135935

[92] J. Lindsey, A. Litwin-Kumar, Theory of systems memory consolidation via recall-gated plasticity. eLife [Preprint] (2023). https://elifesciences.org/reviewed-preprints/90793v1 (Accessed 28 November). 10.7554/eLife.90793 PMC11257680 39023518

[93] W. Sun, M. Advani, N. Spruston, A. Saxe, J. E. Fitzgerald, Organizing memories for generalization in complementary learning systems. Nat. Neurosci. **26**, 1438–1448 (2023). 37474639 10.1038/s41593-023-01382-9 PMC10400413

[94] K. Jacquerie *et al*., Switches to slow rhythmic neuronal activity lead to a plasticity-induced reset in synaptic weights. bioRxiv [Preprint] (2023). 10.1101/2022.07.15.500198 (Accessed 2 July 2023).

[95] P. Leimer, M. Herzog, W. Senn, Synaptic weight decay with selective consolidation enables fast learning without catastrophic forgetting. bioRxiv [Preprint] (2019). 10.1101/613265 (Accessed 17 August 2023).

[96] H. L. Li, M. C. Van Rossum, Energy efficient synaptic plasticity. eLife **9**, e50804 (2020). 32053106 10.7554/eLife.50804 PMC7082127

[97] K. P. Körding, J. B. Tenenbaum, R. Shadmehr, The dynamics of memory as a consequence of optimal adaptation to a changing body. Nat. Neurosci. **10**, 779–786 (2007). 17496891 10.1038/nn1901 PMC2551734

[98] M. F. Carr, S. P. Jadhav, L. M. Frank, Hippocampal replay in the awake state: A potential substrate for memory consolidation and retrieval. Nat. Neurosci. **14**, 147–153 (2011). 21270783 10.1038/nn.2732 PMC3215304

[99] J. M. Murre, TraceLink: A model of amnesia and consolidation of memory. Hippocampus **6**, 675–684 (1996). 9034854 10.1002/(SICI)1098-1063(1996)6:6<675::AID-HIPO10>3.0.CO;2-Y

[100] J. R. Pugh, I. M. Raman, Potentiation of mossy fiber EPSCs in the cerebellar nuclei by NMDA receptor activation followed by postinhibitory rebound current. Neuron **51**, 113–123 (2006). 16815336 10.1016/j.neuron.2006.05.021

[101] T. Hirano, Regulation and interaction of multiple types of synaptic plasticity in a Purkinje neuron and their contribution to motor learning. Cerebellum **17**, 756–765 (2018). 29995220 10.1007/s12311-018-0963-0

[102] L. Mapelli, M. Pagani, J. A. Garrido, E. D’Angelo, Integrated plasticity at inhibitory and excitatory synapses in the cerebellar circuit. Front. Cell. Neurosci. **9**, 169 (2015). 25999817 10.3389/fncel.2015.00169 PMC4419603

[103] M. Najac, I. M. Raman, Integration of Purkinje cell inhibition by cerebellar nucleo-olivary neurons. J. Neurosci. **35**, 544–549 (2015). 25589749 10.1523/JNEUROSCI.3583-14.2015 PMC4293410

[104] M. Uusisaari, T. Knöpfel, Functional classification of neurons in the mouse lateral cerebellar nuclei. Cerebellum **10**, 637–646 (2011). 21116763 10.1007/s12311-010-0240-3 PMC3215887

[105] A. S. Fanning, A. M. Shakhawat, J. L. Raymond, Population calcium responses of Purkinje cells in the oculomotor cerebellum driven by nonvisual input. J. Neurophysiol. **126**, 1391–1402 (2021). 34346783 10.1152/jn.00715.2020 PMC8560417

[106] B. Winkelman, M. Frens, Motor coding in floccular climbing fibers. J. Neurophysiol. **95**, 2342–2351 (2006). 16354726 10.1152/jn.01191.2005

[107] D. M. Lasker, G. C. Han, H. J. Park, L. B. Minor, Rotational responses of vestibular–nerve afferents innervating the semicircular canals in the C57BL/6 mouse. J. Assoc. Res. Otolaryngol. **9**, 334–348 (2008). 18473139 10.1007/s10162-008-0120-4 PMC2538153

[108] M. W. Bagnall, L. E. McElvain, M. Faulstich, S. du Lac, Frequency-independent synaptic transmission supports a linear vestibular behavior. Neuron **60**, 343–352 (2008). 18957225 10.1016/j.neuron.2008.10.002 PMC2614234

[109] M. Beraneck, K. E. Cullen, Activity of vestibular nuclei neurons during vestibular and optokinetic stimulation in the alert mouse. J. Neurophysiol. **98**, 1549–1565 (2007). 17625061 10.1152/jn.00590.2007

[110] E. S. Boyden, J. L. Raymond, Active reversal of motor memories reveals rules governing memory encoding. Neuron **39**, 1031–1042 (2003). 12971901 10.1016/s0896-6273(03)00562-2

[111] C. C. Guo, M. C. Ke, J. L. Raymond, Cerebellar encoding of multiple candidate error cues in the service of motor learning. J. Neurosci. **34**, 9880–9890 (2014). 25057191 10.1523/JNEUROSCI.5114-13.2014 PMC4107405

[112] J. L. Raymond, S. G. Lisberger, Neural learning rules for the vestibulo-ocular reflex. J. Neurosci. **18**, 9112–9129 (1998). 9787014 10.1523/JNEUROSCI.18-21-09112.1998 PMC6793522

[113] A. Suvrathan, H. L. Payne, J. L. Raymond, Timing rules for synaptic plasticity matched to behavioral function. Neuron **92**, 959–967 (2016). 27839999 10.1016/j.neuron.2016.10.022 PMC5165237

[114] V. Lev-Ram, S. T. Wong, D. R. Storm, R. Y. Tsien, A new form of cerebellar long-term potentiation is postsynaptic and depends on nitric oxide but not cAMP. Proc. Natl. Acad. Sci. U.S.A. **99**, 8389–8393 (2002). 12048250 10.1073/pnas.122206399 PMC123077

[115] F. Zenke, W. Gerstner, S. Ganguli, The temporal paradox of Hebbian learning and homeostatic plasticity. Curr. Opin. Neurobiol. **43**, 166–176 (2017). 28431369 10.1016/j.conb.2017.03.015

[116] F. Zenke, G. Hennequin, W. Gerstner, Synaptic plasticity in neural networks needs homeostasis with a fast rate detector. PLoS Comput. Biol. **9**, e1003330 (2013). 24244138 10.1371/journal.pcbi.1003330 PMC3828150

[117] T. D. B. Nguyen-Vu , Cerebellar Purkinje cell activity drives motor learning. Nat. Neurosci. **16**, 1734–1736 (2013). 24162651 10.1038/nn.3576 PMC3966616

[118] B. J. Bhasin, J. L. Raymond, M. S. Goldman, Repository containing code for Bhasin *et al.*, “Synaptic weight dynamics underlying memory consolidation: Implications for learning rules, circuit organization, and circuit function”. GitHub. https://github.com/goldman-lab/consolidation-integration. Deposited 24 July 2024. 10.1073/pnas.2406010121 PMC11474072 39365821

