# Supplementary material for: Synaptic weight dynamics underlying memory consolidation: Implications for learning rules, circuit organization, and circuit function
Source: Proc Natl Acad Sci U S A. 2024 Oct 4;121(41):e2406010121. doi: 10.1073/pnas.2406010121 (PMC11474072; doi:10.1073/pnas.2406010121)
Supplement: Supplementary file 1 — Appendix 01 (PDF) [file pnas.2406010121.sapp.pdf]

Supporting Information for

Synaptic weight dynamics underlying memory consolidation: implications for learning rules, circuit organization, and circuit function

Brandon J Bhasin, Jennifer L Raymond and Mark S Goldman

Jennifer Raymond, Mark Goldman
E-mail: jenr@stanford.edu, msgoldman@ucdavis.edu

This PDF file includes:

Supporting text
Figs. S1 to S3
Tables S1 to S3
SI References

Supporting Information Text

Contents

S1 Analysis of synaptic learning dynamics in the feedforward model . . . 2
S1.1 Post-training dynamics and conditions for stable consolidation . . . 2
S1.2 Flow field analysis and diffusive drift . . . 3
S1.3 Stability during training . . . 4
S1.4 Analysis of drift without error feedback . . . 4
S2 Mitigation of stability-plasticity dilemma with two sites of learning . . . 6
S2.1 Consolidation averages over variability in instructive signals . . . 6
S2.2 Comparing a one-site and two-site model . . . 8
S3 Consolidation with the Hebbian rule is not robust to noisy input . . . 9
S4 Traditional homeostatic Hebbian rules do not readily support consolidation of analog memories . . . 10
S5 Analysis of model dynamics with fixed-strength internal feedback loop . . . 11
S5.1 Conditions for stable post-training consolidation . . . 11
S5.2 Dynamics during training . . . 12
S5.3 Effect of post-training reset on weight dynamics . . . 15
S6 Circuit model with plastic internal feedback . . . 15
S6.1 Model formulation . . . 16
S6.2 Stability of shared linearized dynamics . . . 17
S7 Simulation of oculomotor learning . . . 18

Below, we first provide mathematical analysis of the synaptic learning dynamics of the feedforward circuit models with heterosynaptic (§S1, §S2) or Hebbian (§S3, §S4) plasticity rules at the late-learning site. We then analyze the learning dynamics in recurrent circuit models with either a fixed-strength (§S5) or plastic (§S6) internal feedback pathway.

S1. Analysis of synaptic learning dynamics in the feedforward model. To understand the conditions for successful consolidation in the model, we examined the dynamics of learning analytically. To simplify our analysis, we consider timescales that are relatively long compared to variation in the sensory input, so that the input is zero on average, Eq. (7), and the variability of the sensory input, as measured by the time average of the squared input, is constant,

$$\langle \dot{H}(t)^2 \rangle_{\tau_f} \approx \sigma_H^2. \qquad \text{[S1]}$$

S1.1. Post-training dynamics and conditions for stable consolidation. Using these assumptions, the dynamics of the early-learning site can be described by simplifying Eq. (13) to

$$\tau_w \frac{\mathrm{d}w_H}{\mathrm{d}t} \approx -(w_H(t) + w_H^-) + w_\infty^+ - c(t), \qquad \text{[S2]}$$

where $w_\infty^+ = \mathrm{PF}_0(k_{\mathrm{LTP}} - k_{\mathrm{LTD}}\mathrm{CF}_0)$ depends only on the baseline firing rates of inputs to the Purkinje cell, and $c(t) = k_{\mathrm{LTD}}\langle \delta\mathrm{PF}(t)\delta\mathrm{CF}(t)\rangle_{\tau_f}$ is proportional to the covariance of the parallel fiber-mediated sensory input and climbing fiber feedback about errors. When the circuit is producing behavioral output without error (as we assume is the case before training), or during the post-training period, when there is no feedback about errors, $c(t) \equiv 0$. As a result, w_H tends toward a baseline value of $w_\infty^+ - w_H^-$. If $c(t) > 0$ during training, as was the case in our simulations, w_H decreases from this baseline, increasing the input-output gain of the circuit at the end of the training period. On the other hand, eye movements that are too large would result in $c(t) < 0$, causing an increase in w_H and a decrease in the gain.

At the late-learning site, the heterosynaptic rule, Eq. (14), can be simplified to

$$\frac{\mathrm{d}v}{\mathrm{d}t} \approx -k_{v,\mathrm{hetero}}(\mathrm{MF}_0 \cdot \mathrm{PF}_0 + k_{\mathrm{MF}}k_{\mathrm{PF}}\sigma_H^2)w_H(t), \qquad \text{[S3]}$$

where we assume that the timescale of plasticity of w_H is significantly longer than τ_f (the timescale of correlations in neural activity to which the learning rule is sensitive) and therefore the value of w_H is effectively uncorrelated with the sensory input. From the form of Eq. (S3), the late-learning weight v is proportional to the temporal integral of the early-learning weight w_H.

We can then understand the dynamics of learning as follows. Initially, when errors are large and correlated with the sensory input, because of the saturation in the CF response, we have

$$c(t) \approx k_{\mathrm{LTD}}k_{\mathrm{PF}}k_{\mathrm{CF}}C, \qquad \text{[S4]}$$

where

$$C = \langle |\dot{H}| \rangle_{\tau_{f,w}}. \qquad \text{[S5]}$$

For sinusoidal sensory input with period of oscillation much shorter than $\tau_{f,w}$, $C \approx 2/\pi \cdot v_{\mathrm{peak}}$, which is constant for a fixed choice of peak input amplitude. During training, when $c(t) \neq 0$, w_H tends toward a value that is smaller than baseline by an amount proportional to C (Fig. S1*A*,*B*). This saturation can be understood as long-term depression driven by error being

balanced by the decay in w_H to baseline. As v starts to integrate, this further decreases the magnitude of error. When errors are decreased enough such that $c(t)$ is approximately linear in terms of error magnitude,

$$c(t) \approx k_{\mathrm{LTD}} k_{\mathrm{PF}} k_{\mathrm{CF}} \beta_{\mathrm{light}} (g^{\mathrm{target}} - g(t)) \sigma_H^2. \quad \text{[S6]}$$

In this regime, there is a stable fixed point of the weights that corresponds to the target input-to-output gain (Fig. S1*B*,*C*; see below and §S1.3).

After training ends, w_H returns to baseline. v must also go to a steady state in order for the system to be stable. From Eq. (S3), this occurs as long as w_H has a baseline value of 0, which from Eq. (S2) requires that

$$w_\infty^+ = w_H^-, \quad \text{[S7]}$$

that is, when at steady state the excitatory and inhibitory weights of the sensory input to the early-learning site are balanced. As a result, there is a continuum of steady states of the system in the post-training period, corresponding to a continuum of input-to-output gains of the circuit, since any weight configuration where $w_H = 0$ is a fixed point of the dynamics, which can be represented as a line in w_H-v space (Fig. 2*E*). We note that the stable fixed points of the system during training, as determined by the target gain g^{target}, also lie along the same line (Fig. S1*C*). When the steady-state excitation-inhibition balance condition, Eq. (S7), is met, any initial set of weights will evolve toward some fixed point on the line $w_H = 0$. Therefore, $w_H = 0$ is furthermore a line attractor. This can be visualized by plotting a flow field in w_H-v space (Fig. 2*D*,*E*), where a vector describing the direction of a trajectory at any point is given by $\mathbf{x}(w_H, v) = [\mathrm{d}w_H/\mathrm{d}t, \mathrm{d}v/\mathrm{d}t]$.

The overall dynamics of learning and consolidation can thus be put succinctly: oculomotor errors drive a change Δw_H in the early-learning weight that is temporally integrated into a persistent change Δv in the late-learning weight, as the change in w_H decays away. The need to transform a negative Δw_H into a positive Δv explains why plasticity at v is driven by the *anti*correlation between mossy fiber and Purkinje cell fluctuations in Eq. (14).

A similar analysis for the Hebbian covariance-like rule shows that a line attractor may exist when there is no variability in the sensory input, $\sigma_H^2 = 0$ (Fig. 5*A*,*B*, light lines), but that the introduction of variability collapses the line attractor into a single unstable fixed point, which would not allow the circuit to learn an analog input-to-output gain (Fig. 5*A*,*B*, dark lines; §S3). As discussed in the main text (*Implications for plasticity rules*), simple implementations of classic homeostatic Hebbian learning rules that fix the norm of the weight(s) onto the neuron (1, 2) or attempt to maintain postsynaptic firing rate at a target value (3, 4) also failed to support a continuum of stable weight configurations in the model (see §S4). Our analysis found that a modified Hebbian covariance rule containing a decay could stably support a continuum of steady states even for $\sigma_H^2 > 0$, but this required meeting a fine-tuning condition that depends on the value of σ_H^2 (see §S3).

S1.2. Flow field analysis and diffusive drift. Using the flow field, we can understand how the learning rates at the early- and late-learning sites control the amount of consolidation after a period of training. We assume that the circuit starts in a consolidated state before training, so that during the training period w_H changes from its baseline value of 0 by an amount Δw_H. We also assume that v does not change much during the training period. From Eq. (S2) and Eq. (S3), the slope of the vectors $\mathbf{x}(w_H, v)$ describing the flow during the post-training period is

$$\frac{\mathrm{d}v}{\mathrm{d}w_H} \approx k_{v,\mathrm{hetero}} \tau_w (\mathrm{MF}_0 \cdot \mathrm{PF}_0 + k_{\mathrm{MF}} k_{\mathrm{PF}} \sigma_H^2). \quad \text{[S8]}$$

In other words, geometrically, the weight configuration moves approximately along a line with slope given by Eq. (S8), so that as w_H returns to 0, the change in v during the post-training period is given by $\Delta v \approx -\mathrm{d}v/\mathrm{d}w_H \cdot \Delta w_H$. Since the slope is proportional to $k_{v,\mathrm{hetero}} \tau_w$, a learning rate $k_{v,\mathrm{hetero}}$ at v that is much smaller than the learning rate $1/\tau_w$ at w_H would correspond to slow consolidation, but also a corresponding insensitivity of v to noisy fluctuations in w_H during the post-training period (Fig. 3).

For the circuit to completely consolidate the changes made at the early-learning site during the training period—i.e., preserve the value of the gain g_{train} achieved during training—trajectories need to follow the line in synaptic weight space corresponding to a constant gain (Eq. (8), with $g(t) \equiv g_{\mathrm{train}}$). This is achieved if the slope of the flow field is close to that of the constant gain line,

$$k_{v,\mathrm{hetero}} \tau_w \langle \mathrm{MF} \cdot \mathrm{PF} \rangle_{\tau_{f,v}} \approx \frac{w_{\mathrm{PC}} k_{\mathrm{PF}}}{k_{\mathrm{MF}}}, \quad \text{[S9]}$$

(see Fig. 2*D*, dashed ~~s~~lines). In general, we can write the fraction p of the gain change induced during training that is consolidated post-training as

$$p = \frac{g_{\mathrm{cons}} - g_{\mathrm{pre}}}{g_{\mathrm{train}} - g_{\mathrm{pre}}} = \frac{k_{v,\mathrm{hetero}} \tau_w \langle \mathrm{MF} \cdot \mathrm{PF} \rangle_{\tau_{f,v}}}{w_{\mathrm{PC}} \cdot k_{\mathrm{PF}} / k_{\mathrm{MF}}}, \quad \text{[S10]}$$

where g_{pre} is the gain before training, g_{train} is the gain immediately after training, and g_{cons} is the gain after consolidation. Complete consolidation (i.e., Eq. (S9)) corresponds to $p = 1$.

As noted in the main text, because v is integrating, noise is also accumulated. We examined this by perturbing w_H in the absence of training signals from feedback about errors (see §S7)Extending the current analysis to the case of random perturbations, we can show that, for a sequence of perturbations in w_H that are uniformly distributed over $[-\alpha, \alpha]$, the variance across simulations of the value of v after the kth perturbation will be

$$\mathrm{Var}(v^{(k)}) = \frac{1}{3} \alpha^2 (k_{v,\mathrm{hetero}} \tau_w \langle \mathrm{MF} \cdot \mathrm{PF} \rangle_{\tau_{f,v}})^2 (k + 1 + A(k)), \quad \text{[S11]}$$

where $A(k)$ is an exponentially decaying function of the number of perturbations and the time between perturbations (see §S1.4 for definition and derivation). Thus, the variance is asymptotically linear in k and the behavior of v is diffusive on long timescales. This is the key mathematical observation underlying the "speed-accuracy" tradeoff shown in Fig. 3.

The slope of the flow field is also related to the variability of sensory input σ_H^2. In this way, an input with larger variability would increase the rate of plasticity at v. However, this contribution to the rate of plasticity due to stimulus-driven variability is much smaller than that due to the high baseline firing rates of the mossy fibers MF_0 and parallel fibers PF_0 (not shown; see Eq. (S8)) given the parameters we used, which were chosen to match experimental values.

S1.3. Stability during training. During training, we assume that the climbing fiber modulation $\delta\mathrm{CF}(t)$ is modeled by Eq. (12), i.e., it is a saturating function of the oculomotor (retinal slip) error $\dot{R}(t)$ (Eq. (11)). Then, there is a fixed point of the weight dynamics corresponding to the point at which the measured gain of the system is $g = g^{\mathrm{target}}$,

$$(w_H^*, v^*) = \left(0, \frac{g^{\mathrm{target}}}{k_E k_{\mathrm{MF}}}\right). \qquad \text{[S12]}$$

The dynamics of trajectories leading to a steady state can be separated into two regimes. Initially, the amplitude of oculomotor errors is large, so that $\delta\mathrm{CF}$ saturates, and correspondingly so does the parallel fiber-climbing fiber covariance $c(t)$. As a result, the weight w_H tends toward the value defined by Eq. (S4). From the simplified form of the heterosynaptic plasticity rule for v, Eq. (S3), this leads to initially linear growth in v. As v increases, eventually the amplitude of oculomotor errors will decrease, such that $\delta\mathrm{CF}$ will be a linear function of $\dot{R}$, and from Eq. (S6), $c(t)$ will be a linear function of w_H and v, such that

$$\tau_w \frac{\mathrm{d}w_H}{\mathrm{d}t} \approx -w_H + k_{\mathrm{LTD}} k_{\mathrm{PF}}^2 k_{\mathrm{CF}} \beta_{\mathrm{light}} k_E w_{\mathrm{PC}} \left(\frac{g^{\mathrm{target}} - k_E k_{\mathrm{MF}} v}{k_E k_{\mathrm{PF}} w_{\mathrm{PC}}} - w_H\right) \sigma_H^2. \qquad \text{[S13]}$$

Thus, in the linear regime, the dynamics have eigenvalues

$$\lambda_\pm = -\frac{1}{2\tau_w}(1 + k_{\mathrm{LTD}} k_{\mathrm{PF}}^2 k_{\mathrm{CF}} \beta_{\mathrm{light}} \sigma_H^2 k_E w_{\mathrm{PC}}) \cdot \left[1 \mp \sqrt{1 - \frac{4\tau_w k_{\mathrm{LTD}} k_{\mathrm{PF}} k_{\mathrm{CF}} \beta_{\mathrm{light}} \sigma_H^2 k_E k_{\mathrm{MF}} k_{v,\mathrm{hetero}} \langle \mathrm{MF}(t)\mathrm{PF}(t)\rangle_{\tau_{f,v}}}{(1 + k_{\mathrm{LTD}} k_{\mathrm{PF}}^2 k_{\mathrm{CF}} \beta_{\mathrm{light}} \sigma_H^2 k_E w_{\mathrm{PC}})^2}}\right]. \qquad \text{[S14]}$$

Since all of the parameters are strictly positive (during training, $\sigma_H^2 > 0$), the steady state in Eq. (S12) is a stable fixed point of the dynamics.

The approach to the fixed point could involve decaying oscillations in the values of w_H and v around the steady state. Oscillations do not occur if

$$k_{v,\mathrm{hetero}} \langle \mathrm{MF}(t)\mathrm{PF}(t)\rangle_{\tau_{f,v}} \cdot \tau_w < \frac{(1 + k_{\mathrm{LTD}} k_{\mathrm{PF}}^2 k_{\mathrm{CF}} \beta_{\mathrm{light}} \sigma_H^2 k_E w_{\mathrm{PC}})^2}{4 k_{\mathrm{LTD}} k_{\mathrm{PF}} k_{\mathrm{CF}} \beta_{\mathrm{light}} \sigma_H^2 k_E k_{\mathrm{MF}}}. \qquad \text{[S15]}$$

The above inequality places a constraint on the ratio of the rate of plasticity at v relative to the intrinsic rate of plasticity $(1/\tau_w)$ at w_H to avoid oscillations.

The above analysis shows that, even when the size of climbing fiber responses decreases across learning as a result of decreased errors in the output, stable consolidation is still achieved. Learning at the early-learning site decreases in amplitude as consolidation occurs at the late-learning site until the system reaches desired performance, at which point the early-learning area is no longer required (Fig. S1).

S1.4. Analysis of drift without error feedback. We noted above that consolidation could be understood as temporal integration at the late-learning site v of weight changes initially induced in the early-learning weight w_H. Drawing on other work on integrators, we showed that periodically applied random perturbations of w_H led to the value of v random-walking over time (Fig. 3*A*–*D*). Here, we derive analytically a formula for the variance across sample paths of the late-learning weight v as a function of time and the learning rate at v.

We start from the analysis in §S1.1 above. As in the simulation of Fig. 3*A*–*D* (see §S7), we assumed that the value of w_H was perturbed at a regular interval T, with each perturbation k given by an i.i.d. random value $\Delta w_H^{(k)}$ drawn uniformly over the interval $[-\alpha, \alpha]$. After the perturbation, the synaptic dynamics cause w_H to decay toward a baseline value of zero. To model the effect of each perturbation, we assume the value of w_H immediately following the kth perturbation is given by

$$w_{H,\mathrm{perturb}}^{(k)} = w_{H,\mathrm{post}}^{(k-1)} + \Delta w_H^{(k)}, \qquad \text{[S16]}$$

where the value of w_H just prior to the kth perturbation is given by

$$w_{H,\mathrm{post}}^{(k-1)} = w_{H,\mathrm{perturb}}^{(k-1)} e^{-T/\tau_w}, \qquad \text{[S17]}$$

which models the decay in w_H between perturbations. We can simplify this recurrence relation as

$$\begin{aligned} w_{H,\text{post}}^{(k)} &= e^{-T/\tau_w}(w_{H,\text{post}}^{(k-1)} + \Delta w_H^{(k)}) \\ &= e^{-T/\tau_w}(e^{-T/\tau_w}(w_{H,\text{post}}^{(k-2)} + \Delta w_H^{(k-1)}) + \Delta w_H^{(k)}) \\ &= w_{H,\text{post}}^{(-1)}(e^{-T/\tau_w})^{k+1} + \sum_{k'=0}^{k} \Delta w_H^{(k-k')}(e^{-T/\tau_w})^{k'+1} \\ &= w_{H,\text{post}}^{(-1)}(e^{-T/\tau_w})^{k+1} + \sum_{k'=0}^{k} \Delta w_H^{(k')}(e^{-T/\tau_w})^{k-k'+1}. \end{aligned} \quad \text{[S18]}$$

We assume that the system starts in a stable steady state, i.e., $w_{H,\text{post}}^{(-1)} = 0$. The change during each post-perturbation period $k \geq 0$ is thus

$$\begin{aligned} \Delta w_{H,\text{post}}^{(k)} &= w_{H,\text{post}}^{(k)} - w_{H,\text{perturb}}^{(k)} = w_{H,\text{perturb}}^{(k)}(e^{-T/\tau_w} - 1) \\ &= \left(\sum_{k'=0}^{k-1} \Delta w_H^{(k')}(e^{-T/\tau_w})^{k-k'} + \Delta w_H^{(k)}\right)(e^{-T/\tau_w} - 1) \\ &= (e^{-T/\tau_w} - 1)\sum_{k'=0}^{k} \Delta w_H^{(k')}(e^{-T/\tau_w})^{k-k'}. \end{aligned} \quad \text{[S19]}$$

Then, from Eq. (S8), the change in v during the kth perturbation period can be approximated as

$$\begin{aligned} \Delta v^{(k)} &\approx k_{v,\text{hetero}}\tau_w \langle \text{MF}(t)\text{PF}(t)\rangle_{\tau_{f,v}} \Delta w_{H,\text{post}}^{(k)} \\ &= -k_{v,\text{hetero}}\tau_w \langle \text{MF}(t)\text{PF}(t)\rangle_{\tau_{f,v}} (1 - e^{-T/\tau_w}) \sum_{k'=0}^{k} \Delta w_H^{(k')}(e^{-T/\tau_w})^{k-k'}, \end{aligned} \quad \text{[S20]}$$

using the relationship in Eq. (S19).

We can calculate the overall value of v after the kth perturbation as

$$v^{(k)} = v_0 + \sum_{k'=0}^{k} \Delta v^{(k')}. \quad \text{[S21]}$$

This has variance

$$\begin{aligned} \text{Var}(v^{(k)}) &= \text{Var}\left(v_0 + \sum_{k'=0}^{k} \Delta v^{(k')}\right) = \text{Var}\left(\sum_{k'=0}^{k} \Delta v^{(k')}\right) \\ &= \sum_{k'=0}^{k}\sum_{k''=0}^{k} \text{Cov}(\Delta v^{(k')}, \Delta v^{(k'')}) \\ &= \sum_{k'=0}^{k}\left(2\sum_{k''=0}^{k'} \text{Cov}(\Delta v^{(k')}, \Delta v^{(k'')}) - \text{Var}(\Delta v^{(k')})\right). \end{aligned} \quad \text{[S22]}$$

Thus, to calculate the variance in $v^{(k)}$, we need to calculate the variance of $\Delta v^{(k)}$ and the covariance between $\Delta v^{(k)}$ and $\Delta v^{(j)}$. For convenience, define

$$E = e^{-T/\tau_w}, \text{ and} \quad \text{[S23]}$$

$$K = k_{v,\text{hetero}}\tau_w \langle \text{MF}(t)\text{PF}(t)\rangle_{\tau_{f,v}}. \quad \text{[S24]}$$

We can write the covariance between the change in v due to perturbations j and k as

$$\begin{aligned} \text{Cov}(\Delta v^{(k)}, \Delta v^{(j)}) &= (K(1-E))^2 \sum_{k'=0}^{\min(k,j)} \text{Var}(\Delta w_H^{(k')}) E^{(k+j-2k')} \\ &= \frac{1}{3}\alpha^2 (K(1-E))^2 \sum_{k'=0}^{\min(k,j)} E^{(k+j-2k')}. \end{aligned} \quad \text{[S25]}$$

where, because $\Delta w_H^{(k)}$ is uniformly distributed over $[-\alpha, \alpha]$, $\mathrm{Var}(\Delta w_H^{(k)}) = 1/3 \cdot \alpha^2$. The variance of $\Delta v^{(k)}$ can be found by taking $j = k$ in Eq. (S25),

$$\begin{aligned}\mathrm{Var}(\Delta v^{(k)}) = \mathrm{Cov}(\Delta v^{(k)}, \Delta v^{(k)}) &= \frac{1}{3}\alpha^2 (K(1-E))^2 \sum_{k'=0}^{k} E^{2k'} \\ &= (K(1-E))^2 \cdot \frac{1}{3}\alpha^2 \cdot \frac{1-E^{2(k+1)}}{1-E^2} \\ &= \frac{1}{3}\alpha^2 K^2 \cdot \frac{1-E}{1+E}(1-E^{2(k+1)}). \end{aligned} \tag{S26}$$

We can then calculate the sum

$$\begin{aligned}\sum_{k'=0}^{k} \mathrm{Var}(\Delta v^{(k')}) &= \frac{1}{3}\alpha^2 K^2 \frac{1-E}{1+E} \sum_{k'=0}^{k} (1-E^{2(k'+1)}) \\ &= \frac{1}{3}\alpha^2 K^2 \left(\frac{1-E}{1+E}(k+1) - \frac{E^2(1-E^{2(k+1)})}{(1+E)^2} \right) \end{aligned} \tag{S27}$$

Finally, from Eq. (S25), we can also calculate the sum of covariances

$$\begin{aligned}\sum_{k'=0}^{k} \sum_{k''=0}^{k'} \mathrm{Cov}(\Delta v^{(k')}, \Delta v^{(k'')}) &= \frac{1}{3}\alpha^2 (K(1-E))^2 \sum_{k'=0}^{k} \sum_{k''=0}^{k'} \sum_{k'''=0}^{k''} E^{(k'+k''-2k''')} \\ &= \frac{1}{3}\alpha^2 K^2 \left(\frac{k+1}{1+E} - \frac{E(1-E^{k+1})}{1-E} + \frac{E^3(1-E^{2(k+1)})}{(1-E)(1+E)^2} \right). \end{aligned} \tag{S28}$$

Putting these results together, from Eq. (S22), the variance of $v^{(k)}$ is

$$\begin{aligned}\mathrm{Var}(v^{(k)}) &= \sum_{k'=0}^{k} \left(2 \sum_{k''=0}^{k'} \mathrm{Cov}(\Delta v^{(k')}, \Delta v^{(k'')}) - \mathrm{Var}(\Delta v^{(k')}) \right) \\ &= \frac{1}{3}\alpha^2 K^2 \Bigg(-\frac{1-E}{1+E}(k+1) + \frac{E^2(1-E^{2(k+1)})}{(1+E)^2} \\ &\qquad + 2\left[\frac{k+1}{1+E} - \frac{E(1-E^{k+1})}{1-E} + \frac{E^3(1-E^{2(k+1)})}{(1-E)(1+E)^2} \right] \Bigg) \\ &= \frac{1}{3}\alpha^2 K^2 \left(k+1 - \frac{2E(1-E^{k+1})}{1-E} + \frac{E^2(1-E^{2(k+1)})}{(1-E^2)} \right). \end{aligned} \tag{S29}$$

That is, the term $A(k)$ in Eq. (S11) is given by

$$A(k) = -\frac{2E(1-E^{k+1})}{1-E} + \frac{E^2(1-E^{2(k+1)})}{(1-E^2)}. \tag{S30}$$

S2. Mitigation of stability-plasticity dilemma with two sites of learning. To understand how consolidation helps to mitigate the stability-plasticity dilemma, here we build and analyze simplified discrete models of the weight dynamics. In the first section below, we show that consolidation, as an integration process, causes the circuit to effectively average over the noise in the instructive signal. In the following section, we show that averaging allows the circuit to mitigate a stability-plasticity tradeoff that would occur if the circuit only had one site of plasticity.

S2.1. Consolidation averages over variability in instructive signals. We begin by considering a simplified version of the full feedforward model of Figs. 2 and 3. We assume that w_H returns to a baseline value of zero after each training period, and that v perfectly integrates. During a training session $k \geq 0$, the model receives a retinal slip error signal corresponding to a target gain value $g^{\mathrm{target}} = \hat{g}^{(k)}$ that is randomly drawn from a normal distribution, and learns a fraction q of the change in w_H that would fully minimize the error. From Eq. (8) and Eq. (11), this can be written as

$$w_H^{(k)} = -\frac{q}{k_E k_{\mathrm{PF}} w_{\mathrm{PC}}} \left(\hat{g}^{(k)} - k_E k_{\mathrm{MF}} v^{(k-1)} \right), \tag{S31}$$

so that the output of the circuit after training has measured gain

$$g_{\mathrm{train}}^{(k)} = q\hat{g}^{(k)} + (1-q) k_E k_{\mathrm{MF}} v^{(k-1)}, \tag{S32}$$

where we assume that the change in v during training is negligible, and that $\hat{g}^{(k)} = w_H^{(k)} = v^{(k)} = 0$ for all $k < 0$. During the subsequent post-training period, the model consolidates a fraction p_w of the learned weight change in w_H into v,

$$v^{(k)} = v^{(k-1)} - p_w w_H^{(k)}, \quad \text{[S33]}$$

and w_H returns to a baseline value of zero, so the measured gain after consolidation is

$$g_{\text{cons}}^{(k)} = k_E k_{\text{MF}} v^{(k)}. \quad \text{[S34]}$$

Then, the relative gain change consolidated is

$$p = \frac{g_{\text{cons}}^{(k)} - g_{\text{cons}}^{(k-1)}}{g_{\text{train}}^{(k)} - g_{\text{cons}}^{(k-1)}} = \frac{p_w k_{\text{MF}}}{k_{\text{PF}} w_{\text{PC}}}, \quad \text{[S35]}$$

and we can rewrite the change in v after consolidation and the measured gain as

$$v^{(k)} = (1 - pq)v^{(k-1)} + pq\left(\frac{\hat{g}^{(k)}}{k_{\text{MF}} k_E}\right) \quad \text{[S36]}$$

$$g_{\text{cons}}^{(k)} = (1 - pq)g_{\text{cons}}^{(k-1)} + pq\hat{g}^{(k)}. \quad \text{[S37]}$$

To solve the recurrence relation above, we take the unilateral z-transform of Eq. (S37):

$$G_{\text{cons}}^{(z)} = (1 - pq)z^{-1} G_{\text{cons}}^{(z)} + pq\hat{G}^{(z)},$$

where the capital letters represent z-transformed functions. We can rearrange this as

$$G_{\text{cons}}^{(z)} = \frac{pq}{1 - (1 - pq)z^{-1}} \hat{G}^{(z)},$$

and take the inverse z-transform to find

$$g_{\text{cons}}^{(k)} = \left((1 - pq)^k u(k) * \hat{g}^{(k)}\right) = pq\left(e^{k \log(1-pq)} u(k) * \hat{g}^{(k)}\right), \quad \text{[S38]}$$

where the star operator represents discrete convolution and the discrete unit step function is defined to be $u(k) = 1$ for $k \geq 0$ and 0 for $k < 0$. Intuitively, we can understand the measured gain after consolidation as taking a leaky average of the noisy target gain signal $\hat{g}^{(k)}$ over the recent past with time constant (in terms of training sessions) approximately $-1/\log(1 - pq)$.

Given that $\hat{g}^{(k)}$ is a random variable, we can also understand Eq. (S37) as an autoregressive AR(1) process, whose stationary distribution has mean and distribution

$$\mathbb{E}[g_{\text{cons}}^{(\infty)}] = \mathbb{E}[\hat{g}^{(k)}] \quad \text{[S39]}$$

$$\text{Var}(g_{\text{cons}}^{(\infty)}) = \frac{(pq)^2}{1 - (1 - pq)^2} \text{Var}(\hat{g}^{(k)}). \quad \text{[S40]}$$

That is, the stationary distribution of the measured gain after consolidation has mean equal to the true mean of the noisy target gain distribution, and variance that is an increasing function of the consolidation fraction p.

From Eq. (S38) we can see that a system that consolidates a large fraction of what is learned during the training period, $p \to 1$, averages over a relatively small amount of the recent past—for fast early-learning, $q = 1$, the time constant approaches zero as $p \to 1$. Furthermore, if the mean of the noisy target gain distribution changes, the approach of the consolidated gain to the new true mean will be fast. However, as a result, the variance of the measured gain across training sessions will have a relatively large variance, from Eq. (S40). On the other hand, a system that consolidates slowly ($p \to 0$) averages over a longer period, reaching the true mean slowly but with a lower stationary variance.

These features of the model are illustrated in Figure 4*F*. We plotted the value of the consolidated gain $g_{\text{cons}}^{(k)}$ resulting from simulating the simplified model, Eq. (S31) and Eq. (S33), with $p = 0.1$ ("slow consolidation", magenta) or $p = 0.75$ ("fast consolidation", cyan), in response to the same set of target gain values. Initial values of w_H and v and all other parameters are the same as for the full model above (Table S1). For the first 50 training sessions, target gain values were drawn from a distribution with mean 0.4 and standard deviation 0.1, and for the subsequent 150 sessions, from a distribution with the same standard deviation but with mean 2.

S2.2. Comparing a one-site and two-site model. To see the advantage of the averaging effect described above, we consider a one site version of the simplified model (effectively, the late-learning weight is constant), with weight $w_{H,1}$ and no consolidation. From Eq. (8), the measured gain of this circuit after each training period k is then given by

$$g_1^{(k)} = k_E(k_{\mathrm{MF}}v - k_{\mathrm{PF}}w_{\mathrm{PC}}w_{H,1}^{(k)}),$$

where v is fixed at the initial value v_0. During each training period, the model again learns a fraction q of the total weight change in $w_{H,1}$ required to minimize error,

$$\begin{aligned} w_{H,1}^{(k)} &= w_{H,1}^{(k-1)} + q(w_1^* - w_{H,1}^{(k-1)}) \\ &= (1-q)w_{H,1}^{(k-1)} - \frac{q}{k_E k_{\mathrm{PF}} w_{\mathrm{PC}}}\left(\hat{g}^{(k)} - k_E k_{\mathrm{MF}} v\right), \end{aligned} \quad \text{[S41]}$$

or equivalently in terms of the measured gain,

$$g_1^{(k)} = (1-q)g_1^{(k-1)} + q\hat{g}^{(k)}. \quad \text{[S42]}$$

Note that the weight update, Eq. (S41), is equivalent to the weight update for the two-site model, Eq. (S31), if the weight were reset post-training, i.e., if $w_{H,1}^{(k-1)} = 0$ after consolidation. That is, just as for the two site model, the measured gain of the system is an AR(1) process representing a leaky average of the target gain signal with time constant $-1/\log(1-q)$. For a sequence of target gain values drawn from a normal distribution, the stationary distribution of the measured gain of the circuit will have mean equal to the true mean and variance

$$\mathrm{Var}(g_1^{(\infty)}) = \frac{q^2}{1-(1-q)^2}\mathrm{Var}(\hat{g}^{(k)}) \quad \text{[S43]}$$

which is minimized as q becomes small ("w_H slow" in Fig. 4*A*–*C*). For such a small value of q, the measured gain will remain relatively stable across training sessions, but the circuit will be unable to quickly correct errors. That is, after each training session, the mean squared error of the one-site model in the stationary limit will be

$$\begin{aligned} \mathrm{MSE}_{\mathrm{train},1} &= \lim_{k\to\infty} \mathbb{E}[(\hat{g}^{(k)} - g_1^{(k)})^2] \\ &= (1-q)^2 \lim_{k\to\infty} \mathbb{E}[(\hat{g}^{(k)} - \hat{g}_1^{(k-1)})^2] \\ &= (1-q)^2 \mathrm{MSE}_{\mathrm{pre},1}, \end{aligned} \quad \text{[S44]}$$

where $\mathrm{MSE}_{\mathrm{pre},1}$ is the mean squared error at the start of each training session,

$$\begin{aligned} \mathrm{MSE}_{\mathrm{pre},1} &= \lim_{k\to\infty} \mathbb{E}[(\hat{g}^{(k)} - g_1^{(k-1)})^2] \\ &= \mathbb{E}[(\hat{g}^{(k)})^2] - \lim_{k\to\infty}\left(2\mathbb{E}[\hat{g}^{(k)}]\mathbb{E}[g_1^{(k-1)}] - \mathbb{E}[(g_1^{(k-1)})^2]\right) \\ &= \frac{2}{2-q}\mathrm{Var}(\hat{g}^{(k)}). \end{aligned} \quad \text{[S45]}$$

That is, after training to adapt to a target gain experienced during training session $k-1$, $\mathrm{MSE}_{\mathrm{pre},1}$ represents the mean squared error expected at the start of training session k, when a new target gain value will be drawn. From Eq. (S44) and Eq. (S45), we see that the average error after training is a decreasing function of q, whereas the average error at the start of training is an increasing function of q (for $q < 2$). Hence, there is a tradeoff between the ability of the circuit to correct errors quickly during a training session and the size of the expected future error given a noisy target gain.

Repeating this calculation for the model with two sites of learning, we have

$$\begin{aligned} \mathrm{MSE}_{\mathrm{train},2} &= \lim_{k\to\infty} \mathbb{E}[(\hat{g}^{(k)} - g_{\mathrm{train}}^{(k)})^2] \\ &= (1-q)^2 \lim_{k\to\infty} \mathbb{E}[(\hat{g}^{(k)} - g_{\mathrm{cons}}^{(k-1)})^2] \\ &= (1-q)^2 \mathrm{MSE}_{\mathrm{pre},2}, \end{aligned} \quad \text{[S46]}$$

and

$$\begin{aligned} \mathrm{MSE}_{\mathrm{pre},2} &= \lim_{k\to\infty} \mathbb{E}[(\hat{g}^{(k)} - g_{\mathrm{cons}}^{(k-1)})^2] \\ &= \frac{2}{2-pq}\mathrm{Var}(\hat{g}^{(k)}). \end{aligned} \quad \text{[S47]}$$

Thus, the average error after training can be reduced by fast learning at the early-learning site ($q \to 1$), and the average error at the start of training can be simultaneously reduced by slow learning at the late-learning site ($p \to 0$).

In Figure 4*C,E*, we plotted the one-site tradeoff curve as functions of the early site learning rate q ("w_H slow" and "w_H fast" arrows), with $\mathrm{MSE}_{\mathrm{pre},1}$ on the x-axis and $\mathrm{MSE}_{\mathrm{train},1}$ on the y-axis (black line in Fig. 4*C* and grey line in *E*), normalized by

plotting in units of the variance of the target gain distribution. For the two-site model (Fig. 4E), we plotted the normalized $\text{MSE}_{\text{pre},2}$ on the x-axis and normalized $\text{MSE}_{\text{train},2}$ on the y-axis for $p = 0.1$ ("v slow", magenta) and $p = 0.75$ ("v fast", cyan), while again varying q. In Figure 4B, we plotted the post-training gain $g_1^{(k)}$ from a simulation of the simple one-site model with $q = 0.1$ ("slow learning", black) or $q = 0.75$ ("fast learning", grey) over 200 training sessions, where the target gain value for each session was independently drawn from a normal distribution with mean 0.4 and standard deviation 0.1. The values of $\text{MSE}_{\text{pre},1}$ and $\text{MSE}_{\text{train},1}$ corresponding to these values of q, calculated from Eq. (S44) and Eq. (S45), are plotted in Figure 4C with circles.

To build intuition for the difference between the one-site and two-site models more concretely, in Figure 4A,D we show the time course of the gain and the corresponding squared error (retinal slip) for two subsequent example training sessions. Figure 4D was generated by simulating the full two-site model with weight dynamics controlled by Eq. (S2) and Eq. (14). We set the learning rate at v to $k_{v,\text{hetero}} = 1.29 \times 10^{-6} (\text{s/sp})^2/\text{h}$, which resulted in a fraction of learning consolidated $p \approx 0.13$ for the kth and $p \approx 0.27$ for the $(k+1)$th sessions. All other parameters were the same as those in Table S1, and the initial value of v was set so that the system would have an initial gain of 0.4, as above. To similarly simulate the time course of learning in the one-site model (Fig. 4A), we modified the full two-site model by removing learning at v, and the learning at w_H was governed by

$$\frac{\text{d}w_H}{\text{d}t} = k_w (k_{\text{LTP}} \langle \text{PF}(t) \rangle_{\tau_{f,w}} - k_{\text{LTD}} \langle \text{PF}(t)\text{CF}(t) \rangle_{\tau_{f,w}}), \qquad \text{[S48]}$$

where we set the learning rate parameter $k_w = 0.191\ \text{h}^{-1}$ (light colors) or $k_w = 1.97\ \text{h}^{-1}$ (dark colors), so that for the two training sessions shown the fraction learned was $q \approx 0.10$ for the kth session and $q \approx 0.11$ for the $(k+1)$th session (light colors), or $q \approx 0.75$ for the kth and $q \approx 0.80$ for the $(k+1)$th sessions (dark colors). Unlike the two-site model, we set $k_{\text{LTP}} = 0.648$ s/sp, so that outside of the training period, $\text{d}w/\text{d}t \approx 0$. The model used the same initial values, as well as all other parameters not specified here, as the two-site model for w_H and v. For both models, we simulated two periods of 0.5 h training followed by 11.5 h post-training without feedback about behavioral errors (post-training periods clipped for illustrative purposes).

S3. Consolidation with the Hebbian rule is not robust to noisy input. The failure of the stabilized Hebbian rule, Eq. (15), to successfully consolidate memory from the early- to late-learning site in the presence of variability in the sensory input (Fig. 5) can be understood through an analysis similar to the one performed for the heterosynaptic rule (§S1). We again assume that the mean of the sensory input is approximately zero and that the variability σ_H^2 is constant over the timescales $\tau_{f,v}$ and τ_s. We also assume, as before, that the timescale over which the weights change is much slower than $\tau_{f,v}$. Using that the threshold $\theta(t)$ is the exponential average of $\text{MVN}(t)$, we have

$$\theta(t) = \langle \text{MVN}(t) \rangle_{\tau_s} \approx \text{MF}_0 \langle v \rangle_{\tau_s} - w_{\text{PC}} \text{PF}_0 \langle w \rangle_{\tau_s} - w_{\text{PC}} \text{PC}_0 + \text{MVN}_0. \qquad \text{[S49]}$$

Then, substituting this and the definition of $\text{MVN}(t)$, Eq. (4), into the learning rule, Eq. (15) becomes

$$\frac{\text{d}v}{\text{d}t} \approx k_{v,\text{Hebb}} [\langle \text{MF}(t)^2 \rangle_{\tau_{f,v}} v - w_{\text{PC}} \langle \text{MF}(t)\text{PF}(t) \rangle_{\tau_{f,v}} w_H - \text{MF}_0^2 v_s + w_{\text{PC}} \text{MF}_0 \text{PF}_0 w_{H,s}], \qquad \text{[S50]}$$

where $w_{H,s} = \langle w_H \rangle_{\tau_s}$ and $v_s = \langle v \rangle_{\tau_s}$.

In the post-training period, when $c \to 0$, we have that $w_H \to w_{H,0} = w_\infty^+ - w_H^-$. As w_H approaches this steady state, so will the exponential average, i.e., $w_{H,s} \to w_{H,0}$ (see Eq. (6)), so we can use this quasi-steady state assumption on w_H to simplify Eq. (S50) further to

$$\frac{\text{d}v}{\text{d}t} \approx \frac{1}{\tau_v(\sigma_H^2)} v - k_{v,\text{Hebb}} [w_{\text{PC}} k_{\text{MF}} k_{\text{PF}} \sigma_H^2 w_{H,0} + \text{MF}_0^2 v_s] \quad \text{for } w_H, w_{H,s} \to w_0, \qquad \text{[S51]}$$

where the time constant τ_v is a function of the variability of the sensory input, σ_H^2,

$$\tau_v(\sigma_H^2) = \frac{1}{k_{v,\text{Hebb}} (\text{MF}_0^2 + k_{\text{MF}}^2 \sigma_H^2)}. \qquad \text{[S52]}$$

In the case that there is no variability in the sensory input, $\sigma_H^2 = 0$, we have that the dynamics of learning are given by

$$\tau_v(0) \frac{\text{d}v}{\text{d}t} \approx v - v_s, \qquad \text{[S53]}$$

$$\tau_s \frac{\text{d}v_s}{\text{d}t} = -v_s + v. \qquad \text{[S54]}$$

Here, the first eigenvalue of the system is 0, with corresponding eigenvector $[1, 1]$ so that any state along the line $v = v_s$ is a fixed point of the system. The second eigenvalue of the dynamics is

$$\lambda = \frac{1}{\tau_v(0)} - \frac{1}{\tau_s}, \qquad \text{[S55]}$$

which is decaying if $\tau_s < \tau_v(0)$, i.e., the timescale of the sliding average is faster than the timescale of plasticity (cf. ref. 5). Thus, the synaptic learning dynamics contain a line attractor in the space of v and the internal variable v_s that does not depend on w_0. Geometrically, when $\sigma_H^2 = 0$, the nullclines for the dynamics of v and v_s overlap, creating the line attractor.

If there is variability in the sensory input, $\sigma_H^2 > 0$, the nullclines no longer exactly overlap; there is now a single fixed point $v^* = v_s^* = w_{H,0}$. This fixed point is unstable (it is a saddle point of the dynamics), with eigenvalues

$$\lambda_\pm = \left(\frac{1}{\tau_v(\sigma_H^2)} - \frac{1}{\tau_s}\right)\cdot\frac{1}{2}\left[1 \mp \sqrt{1 + \frac{4k_{v,\mathrm{Hebb}}k_{\mathrm{MF}}^2\sigma_H^2}{\tau_s(1/\tau_v(\sigma_H^2) - 1/\tau_s)^2}}\right],$$

implying that one of the eigenvalues will always be positive.

In principle, the instability can be rectified by adding an additional decay term $-\varepsilon v$ to the learning rule, so that Eq. (15) is

$$\frac{\mathrm{d}v}{\mathrm{d}t} = -\varepsilon v + \langle \mathrm{MF}(t)(\mathrm{MVN}(t) - \theta(t))\rangle_{\tau_{f,v}}. \qquad [\mathrm{S56}]$$

If we write the decay rate as $\varepsilon = k_{v,\mathrm{Hebb}}k_{\mathrm{MF}}^2\sigma^2$ in terms of a new variable σ^2, we can simplify this equation (as we did for Eq. (S51)) to

$$\frac{\mathrm{d}v}{\mathrm{d}t} \approx k_{v,\mathrm{Hebb}}[k_{\mathrm{MF}}^2(\sigma_H^2 - \sigma^2)v + \mathrm{MF}_0^2(v - v_s) - w_{\mathrm{PC}}k_{\mathrm{MF}}k_{\mathrm{PF}}\sigma_H^2 w_0]. \qquad [\mathrm{S57}]$$

If σ^2 is equal to σ_H^2, the variability of the vestibular input in some period, and if $w_{H,0} = 0$, then the system will again be described by the dynamics given by Eq. (S53) and Eq. (S54). That is, $v = v_s$ will still be a line attractor. If σ^2 is not exactly equal to σ_H^2, then the weight will either grow unstably or decay to zero, depending on whether $\sigma^2 < \sigma_H^2$ or $\sigma^2 > \sigma_H^2$, respectively. This can be seen by assuming the threshold θ moves instantaneously, so that $v_s \approx v$, and noting that Eq. (S57) becomes either exponential growth or decay, with time constant inversely proportional to the degree of mistuning. So, for successful systems consolidation in a circuit using this modified version of the Hebbian rule, the learning rule must “know" or otherwise measure the variability of the input σ_H^2. While a learning rule that accounts for the natural statistics of inputs presents an interesting possibility, we note that it may be challenging to implement biologically.

S4. Traditional homeostatic Hebbian rules do not readily support consolidation of analog memories. It has long been recognized that the basic form of the Hebbian learning rule generally leads to unstable positive feedback (1, 6, 7), leading to the development of rules which contain homeostatic mechanisms that attempt to counter this instability. The most well-known classes of such homeostatic mechanisms include weight normalization, in which weights are adjusted so that the weight vector for a neuron maintains a given length (1, 2, 8), and firing rate-target homeostasis methods, in which weights are adjusted so that the mean postsynaptic firing rate is maintained close to a specified target. The latter class of mechanisms includes those commonly called synaptic scaling (3, 9, 10). In order to stably consolidate analog memories, the learning rule at the late-learning site must be able to support a continuum of stable weight configurations. For the circuit model we developed here, we show below that Oja's rule and the BCM rule, prominent examples of weight normalization and firing rate-target homeostasis rules, respectively, cannot support such a stable weight continuum.

First, we examine Oja's rule, a form of weight normalization (2). We will consider the Hebbian piece in a “covariance" form,

$$\frac{\mathrm{d}v}{\mathrm{d}t} = \eta(\langle \delta\mathrm{MF}(t)\delta\mathrm{MVN}(t)\rangle - \langle \delta\mathrm{MVN}^2\rangle v), \qquad [\mathrm{S58}]$$

where for simplicity, $\delta\mathrm{MF}(t)$ and $\delta\mathrm{MVN}(t)$ are baseline-subtracted forms of mossy fiber and MVN neuron firing. We assume that the timescale of the average indicated by the brackets is slow compared to variation in the input but faster than the timescale of plasticity of v (as for τ_f in the main text). Both before training and after consolidation, when $w \to 0$, so that $\mathrm{PC}(t) \to \mathrm{PC}_0$,

$$\delta\mathrm{MVN}(t) = v\delta\mathrm{MF}(t).$$

Then, the learning rule is at steady state when

$$v^*\langle \delta\mathrm{MF}^2\rangle - {v^*}^3\langle \delta\mathrm{MF}^2\rangle = 0 \Rightarrow v^* = 0 \text{ or } v^* = \pm 1.$$

This is because Oja's rule is designed to maintain the Euclidean length of the weight vector fixed at 1, and here there is only one input source with a plastic weight. Even in the case where there are multiple mossy fiber input types with plastic weights, Oja's rule will drive the weight vector so that it is parallel (or antiparallel) to the eigenvector of the covariance matrix of the inputs that has largest eigenvalue (2). In our model, in which we do not expect the signals carried by the mossy fiber inputs to themselves change persistently as a result of training, only one stable weight configuration can be maintained. Thus, Oja's rule does not readily support consolidation in our model.

We next examine the Bienenstock-Cooper-Monroe (BCM) rule (4), one of the most well-known firing rate-target homeostasis rules. The BCM rule consists of a Hebbian term multiplied by a term that changes the sign of plasticity depending on whether postsynaptic activity is greater than or less than a sliding threshold. For our circuit, this takes the form

$$\tau_v\frac{\mathrm{d}v}{\mathrm{d}t} = \langle \mathrm{MF}(t)\mathrm{MVN}(t)\rangle[\langle \mathrm{MVN}(t)\rangle - \theta(t)], \qquad [\mathrm{S59}]$$

$$\tau_\theta\frac{\mathrm{d}\theta}{\mathrm{d}t} = -\theta + \frac{\langle \mathrm{MVN}(t)\rangle^2}{\mathrm{MVN}_{\mathrm{target}}}. \qquad [\mathrm{S60}]$$

The angle brackets again represent time averages taken over a timescale longer than the variation in the input, but much shorter than the timescale of plasticity. The system has a fixed point whenever $\theta = \langle \mathrm{MVN} \rangle = \mathrm{MVN}_{\mathrm{target}}$.

There is one fixed point, with steady state weight value

$$v^* = \frac{\mathrm{MVN}_{\mathrm{target}} - \mathrm{MVN}_0 + w_{\mathrm{PC}} \langle \mathrm{PC} \rangle}{\mathrm{MF}_0}.$$

The value of v^* is determined by parameters of the system that do not change as a result of learning ($\mathrm{MF}_0, \mathrm{MVN}_{\mathrm{target}}$ and MVN_0), as well as a variable ($\langle \mathrm{PC} \rangle$) that returns to its pre-training baseline value during the post-training period. Therefore, the system with one plastic synapse cannot persistently hold all possible values of v that may be reached after training.

For multiple plastic inputs to the (linear) MVN neuron, the BCM rule is less restrictive than Oja's rule: there is a hyperplane—i.e., a continuum—in synaptic weight space of stable weight configurations for which the homeostatic condition of postsynaptic firing rate reaching a target is achieved. This opens the possibility that a more complex model of the circuit could allow consolidation of an analog memory using the BCM rule. However, such a rule would not allow for the weight of a single input type to change independently of the others, unlike the heterosynaptic rule.

S5. Analysis of model dynamics with fixed-strength internal feedback loop. In this section, we analyze the dynamics of the model with a fixed-strength internal feedback loop (Materials and Methods, *Circuit model with internal feedback loop*). We first show the conditions under which stable post-training consolidation occurs. We then find conditions under which the circuit can also stably learn a target input-to-output gain during training.

S5.1. Conditions for stable post-training consolidation. For stable consolidation, we need the late-learning site to stably hold any value of v. Following the same line of reasoning leading to Eq. (S3), we can rewrite the heterosynaptic plasticity rule, Eq. (28), in terms of the weights as

$$\frac{\mathrm{d}v}{\mathrm{d}t} \approx -k_{v,\mathrm{hetero}} \frac{k_{\mathrm{MF}}(\tilde{w}_H(t) - k_E \tilde{w}_E \tilde{v}(t))}{1 - w_{\mathrm{PC}} k_E \tilde{w}_E} \sigma_H^2. \qquad \text{[S61]}$$

Note that, unlike the feedforward model, there must be variability in the post-training input, $\sigma_H^2 > 0$, for consolidation to occur. From Eq. (S61), v is at steady state as long as the feedforward weight at the early-learning weight is

$$w_H = \frac{k_E}{k_{\mathrm{PF},H}} \tilde{w}_E \tilde{v}, \qquad \text{[S62]}$$

which defines a line in w_H-v space (Fig. 6*E,J*). Values of w_H and v that lie along this line cause the early-learning area to not respond to sensory input to the circuit, i.e., the reset condition is satisfied. If $w_E > 0$, this can no longer be achieved in general by a passive decay at w_H to zero, as w_H needs to go to a positive steady state value since v is also positive. Below we evaluate the conditions under which this occurs for the two post-training reset mechanisms.

Inhibitory plasticity-driven resetting In the inhibitory plasticity-driven reset mechanism, the weight of feedforward inhibition onto the early-learning area is governed by a Hebbian-like covariance rule, Eq. (29):

$$\frac{\mathrm{d}w_H^-}{\mathrm{d}t} = k_{\mathrm{inh}} \langle \mathrm{PF}_H(t)(\mathrm{PC}(t) - \langle \mathrm{PC}(t) \rangle_{\tau_f}) \rangle_{\tau_{\mathrm{inh}}}.$$

During the post-training period, the correlation between climbing fibers and the feedforward parallel fibers is zero, $c_H(t) \equiv 0$, so simplifying as for Eq. (S61), and combining with the learning rule for the excitatory weight, Eq. (27), we can write the equation for the overall feedforward weight at the early-learning site as

$$\frac{\mathrm{d}w_H}{\mathrm{d}t} \approx -\frac{k_{\mathrm{inh}} k_{\mathrm{PF},H}^2 \sigma_H^2}{1 - w_{\mathrm{PC}} k_E \tilde{w}_E} \left(w_H - \frac{k_E}{k_{\mathrm{PF},H}} \tilde{w}_E \tilde{v} \right), \qquad \text{[S63]}$$

which has a fixed point that lies along the line in Eq. (S62), satisfying the reset condition, as long as

$$w_E < \frac{1}{k_{\mathrm{PF},E} w_{\mathrm{PC}} k_E}. \qquad \text{[S64]}$$

To determine whether the fixed point is attractive, it suffices to show that the slope of the trajectory approaching the fixed point is smaller than the slope of the line in Eq. (S62). Otherwise, trajectories will move continuously away from the line of fixed points. From Eq. (S63) and Eq. (S61), trajectories have slope

$$\frac{\mathrm{d}v}{\mathrm{d}w_H} \approx \frac{k_{v,\mathrm{hetero}} k_{\mathrm{MF}}}{k_{\mathrm{inh}} k_{\mathrm{PF},H}}, \qquad \text{[S65]}$$

whereas from Eq. (S62) the slope of the line of fixed points is $k_{\mathrm{PF},H} / (k_E \tilde{w}_E k_{\mathrm{MF}})$. Thus, the slope of trajectories is smaller if

$$\frac{k_{v,\mathrm{hetero}} k_{\mathrm{MF}}^2 k_E \tilde{w}_E}{k_{\mathrm{inh}} k_{\mathrm{PF},H}^2} = \frac{\tau_{w_H,\mathrm{dark}}(\sigma_H^2)}{\tau_v(\sigma_H^2)} < 1, \qquad \text{[S66]}$$

where the time constants $\tau_{w_H,\text{dark}}$ and τ_v are defined from Eq. (S63) and Eq. (S61) respectively as functions of the input variability σ_H^2,

$$\tau_{w_H,\text{dark}}(\sigma_H^2) = \frac{1 - w_{\text{PC}} k_E \tilde{w}_E}{k_{\text{reset}} k_{\text{PF},H}^2 \sigma_H^2} \qquad \text{[S67]}$$

$$\tau_v(\sigma_H^2) = \frac{1 - w_{\text{PC}} k_E \tilde{w}_E}{k_{v,\text{hetero}} k_{\text{MF}}^2 k_E \tilde{w}_E \sigma_H^2}, \qquad \text{[S68]}$$

and where, to highlight the similarity of these expressions to the analogous quantities for the other reset mechanism below, we have defined

$$k_{\text{reset}} = k_{\text{inh}} \quad \text{(inhibitory plasticity reset mechanism)}.$$

In other words, the line in Eq. (S62) is an attractor only if plasticity is faster at the early-learning site than at the late-learning site.

The inhibitory plasticity here drives w_H so that the activity of the early-learning area does not modulate in response to sensory input. This result is broadly consistent with previous work showing that Hebbian plasticity of inhibitory synapses tends toward restoring excitatory-inhibitory balance (11), except here this balance is only in terms of the fluctuating components of the inputs and not the spontaneous components, because of the sliding threshold term in Eq. (29). Note that, if $\sigma_H^2 = 0$, early-learning activity will not be reset, but there will also be no consolidation at v.

Inhibition of instructive signals-driven resetting In the inhibition of instructive signals-driven reset mechanism, the activity of the pathway carrying instructive signals is inhibited by an output pathway from the late-learning area (Fig. 6*F*; Eq. (30)):

$$\delta\text{CF}(t) = k_{\text{CF}} \tanh\left(-\beta_{\text{light}} \dot{R}(t) + \beta_{\text{reset}} \delta\text{PC}(t)\right),$$

where $\delta\text{PC}(t) = \text{PC}(t) - \langle \text{PC}(t) \rangle_{\tau_f}$. When information about errors is not present, $\dot{R} \equiv 0$, so that for small errors, the parallel fiber-climbing fiber covariance $c_H(t)$ is in the linear regime, and the learning rule for w_H becomes

$$\frac{\mathrm{d}w_H}{\mathrm{d}t} \approx -\frac{k_{\text{LTD}} k_{\text{CF}} \beta_{\text{reset}} k_{\text{PF},H}^2 \sigma_H^2}{1 - w_{\text{PC}} k_E \tilde{w}_E} \left(w_H - \frac{k_E}{k_{\text{PF},H}} \tilde{w}_E \tilde{v} \right), \qquad \text{[S69]}$$

which has the same fixed points as Eq. (S63) that satisfy the reset condition if Eq. (S64) holds. Similarly, as in Eq. (S66), the fixed point for a given trajectory is attractive if $\tau_{w_H,\text{dark}} < \tau_v$, where $\tau_{w_H,\text{dark}}$ and τ_v are defined as in Eq. (S67) and Eq. (S68) but where we now define

$$k_{\text{reset}} = k_{\text{LTD}} k_{\text{CF}} \beta_{\text{reset}} \quad \text{(inhibition of instructive signals reset mechanism)}.$$

For large errors, the magnitude of δCF will be saturated, and $\mathrm{d}w_H/\mathrm{d}t$ will reach a maximum value, so the slope of trajectories will be

$$\frac{\mathrm{d}v}{\mathrm{d}w_H} \approx \frac{k_{v,\text{hetero}} k_{\text{MF}} \sigma_H^2}{k_{\text{LTD}} k_{\text{CF}} C (1 - w_{\text{PC}} k_E \tilde{w}_E)} \left(w_H - \frac{k_E}{k_{\text{PF},H}} \tilde{w}_E \tilde{v} \right), \qquad \text{[S70]}$$

where C is defined as in Eq. (S5). That is, the trajectories become increasingly vertical the further they are from the line of fixed points. For trajectories to converge to a stable fixed point, they must have slopes shallower than the line of fixed points. This occurs when

$$\left| w_H - \frac{k_E}{k_{\text{PF},H}} \tilde{w}_E \tilde{v} \right| < \frac{k_{\text{LTD}} k_{\text{CF}} k_{\text{PF},H} C (1 - w_{\text{PC}} k_E \tilde{w}_E)}{k_{v,\text{hetero}} k_{\text{MF}}^2 \sigma_H^2 k_E \tilde{w}_E}. \qquad \text{[S71]}$$

This effectively places a limit on how large a gain change the circuit can learn during the training period in order for that gain change to be stably consolidated (see §S5.2, *Inhibition of instructive signals-driven resetting*).

S5.2. Dynamics during training. Here we analyze the dynamics of the synaptic weights in the circuit during training for each mechanism, and show that under similar conditions as post-training, both mechanisms are also stable during training and result in the circuit correctly learning the target input-to-output computation (Fig. S3).

Local stability of the fixed point during training We first show that both mechanisms have the same locally stable fixed point, corresponding to the circuit achieving the target gain. For both mechanisms, the learning rule for the late-learning weight v is identical, Eq. (S61), and the only difference comes from the effect on the early-learning feedforward weight w_H. We assume that the weight of the feedback pathway is fixed and that the total feedback strength is less than 1, as in Eq. (S64). We also assume that $w_{H,\infty}^+ = 0$ and that during training the variability of the head input is $\langle \dot{H}^2 \rangle_{\tau_f} = \sigma_H^2 > 0$. In our analysis, we make use of the fact that, from Eq. (23), the error in gain can be expanded as

$$g^{\text{target}} - g(t) = -\frac{k_E w_{\text{PC}} k_{\text{PF},H}}{1 - w_{\text{PC}} k_E \tilde{w}_E} (w_{H,\text{opt}}^*(v(t)) - w_H(t)), \qquad \text{[S72]}$$

and for convenience we use variables with tildes as defined in Eq. (24)–Eq. (26), and where

$$w_{H,\text{opt}}^*(v) = -\frac{1}{w_{\text{PC}} k_{\text{PF},H}} \left(\frac{g^{\text{target}}}{k_E} (1 - w_{\text{PC}} k_E \tilde{w}_E) - k_{\text{MF}} v \right) \qquad \text{[S73]}$$

is the value of w_H that would minimize the error in gain for a fixed choice of v. Similarly, we define the value of w_H that would reset early-learning activity for a fixed choice of v as

$$w^*_{H,\text{reset}}(v) = \frac{k_E \tilde{w}_E k_{\text{MF}} v}{k_{\text{PF},H}}. \tag{S74}$$

For the inhibitory plasticity mechanism (Fig. 6*A*), changes in the excitatory weight w_H^+ are governed by instructive climbing fiber input driven by the retinal slip signal, Eq. (12), according to the learning rule in Eq. (27), whereas the inhibitory weight w_H^- tends to drive the activity of the early-learning area back to its long-term average, as in Eq. (29). More precisely, for a target gain value g^{target}, plasticity at w_H^+ is driven by the parallel fiber-climbing fiber covariance

$$\begin{aligned} c_H(t) &= k_{\text{LTD}} \langle \delta \text{PF}_H(t) \delta \text{CF}(t) \rangle_{\tau_{f,w}} \\ &= k_{\text{LTD}} k_{\text{CF}} k_{\text{PF},H} \langle \dot{H}(t) \tanh[\beta_{\text{light}}(g^{\text{target}} - g(t)) \dot{H}(t)] \rangle_{\tau_{f,w}}. \end{aligned} \tag{S75}$$

We can combine the learning rules for w_H^+ and the inhibitory weight w_H^- to write an expression for the change in the net weight $w_H = w_H^+ - w_H^-$, and simplify using the same logic as for the post-training period (see §S1). This yields

$$\frac{\mathrm{d}w_H}{\mathrm{d}t} \approx k_{\text{LTD}} k_{\text{CF}} k_{\text{PF},H} \left\langle \dot{H} \tanh\left[\beta_{\text{light}} \frac{k_E w_{\text{PC}} k_{\text{PF},H}}{1 - w_{\text{PC}} k_E \tilde{w}_E} (w^*_{H,\text{opt}}(v) - w_H) \dot{H}\right] \right\rangle_{\tau_{f,w}} + \frac{k_{\text{inh}} k^2_{\text{PF},H} \sigma^2_H}{1 - w_{\text{PC}} k_E \tilde{w}_E} (w^*_{H,\text{reset}}(v) - w_H), \tag{S76}$$

using the simplifications in Eq. (S72)–Eq. (S74).

For the inhibition of instructive signals mechanism (Fig. 6*F*), the climbing fiber input is both driven by retinal slip error and inhibited by a pathway carrying Purkinje cell output, Eq. (30), leading to a parallel fiber-climbing fiber covariance

$$c_H(t) = k_{\text{LTD}} k_{\text{CF}} k_{\text{PF},H} \langle \dot{H}(t) \tanh[\beta_{\text{light}}(g^{\text{target}} - g(t)) \dot{H}(t) + \beta_{\text{reset}} \delta \text{PC}(t)] \rangle_{\tau_{f,w}}. \tag{S77}$$

This yields a simplified learning rule for w_H:

$$\frac{\mathrm{d}w_H}{\mathrm{d}t} \approx k_{\text{LTD}} k_{\text{CF}} k_{\text{PF},H} \left\langle \dot{H} \tanh\left[\left(\beta_{\text{light}} \frac{k_E w_{\text{PC}} k_{\text{PF},H}}{1 - w_{\text{PC}} k_E \tilde{w}_E} (w^*_{H,\text{opt}}(v) - w_H) + \frac{\beta_{\text{reset}} k_{\text{PF},H}}{1 - w_{\text{PC}} k_E \tilde{w}_E} (w^*_{H,\text{reset}}(v) - w_H)\right) \dot{H}\right] \right\rangle_{\tau_{f,w}}. \tag{S78}$$

Both mechanisms have the same fixed point,

$$(w^*_H, v^*) = \left(\frac{k_{\text{PF},E} w_E g^{\text{target}}}{k_{\text{PF},H}}, \frac{g^{\text{target}}}{k_E k_{\text{MF}}} \right), \tag{S79}$$

at which point the gain is equal to g^{target} and $w^*_H = w^*_{H,\text{opt}}(v^*) = w^*_{H,\text{reset}}(v^*)$. Furthermore, the fixed point lies along the post-training line attractor, Eq. (S62). Close to the fixed point, the dynamics for both mechanisms are linear, as the hyperbolic tangent term is in an approximately linear regime,

$$\frac{\mathrm{d}w_H}{\mathrm{d}t} \approx -\frac{1}{\tau_{w_H,\text{light}}(\sigma^2_H)} [w_H - (\alpha w^*_{H,\text{opt}}(v) + (1-\alpha) w^*_{H,\text{reset}}(v))]. \tag{S80}$$

where we defined

$$\alpha = \frac{k_{\text{light}}}{k_{\text{light}} + k_{\text{reset}}}, \text{ and} \tag{S81}$$

$$\tau_{w_H,\text{light}}(\sigma^2_H) = \frac{1 - w_{\text{PC}} k_E \tilde{w}_E}{(k_{\text{light}} + k_{\text{reset}}) k^2_{\text{PF},H} \sigma^2_H} = (1-\alpha) \tau_{w_H,\text{dark}}(\sigma^2_H), \tag{S82}$$

with

$$k_{\text{light}} = k_{\text{LTD}} k_{\text{CF}} \beta_{\text{light}} k_E w_{\text{PC}}, \tag{S83}$$

and

$$k_{\text{reset}} = \begin{cases} k_{\text{inh}} & \text{for the inhibitory plasticity mechanism} \\ k_{\text{LTD}} k_{\text{CF}} \beta_{\text{reset}} & \text{inhibition of instructive signals} \end{cases}. \tag{S84}$$

From Eq. (S61) and Eq. (S80), the Jacobian is

$$\mathbf{J} = -\frac{\sigma^2_H}{1 - w_{\text{PC}} k_E \tilde{w}_E} \cdot \begin{pmatrix} (k_{\text{light}} + k_{\text{reset}}) k^2_{\text{PF},H} & -k_{\text{MF}} k_{\text{PF},H} k_E (k_{\text{reset}} \tilde{w}_E + k_{\text{LTD}} k_{\text{CF}} \beta_{\text{light}}) \\ k_{v,\text{hetero}} k_{\text{MF}} k_{\text{PF},H} & -k_{v,\text{hetero}} k^2_{\text{MF}} k_E \tilde{w}_E \end{pmatrix}. \tag{S85}$$

The eigenvalues of the Jacobian are

$$\lambda_\pm = \frac{1}{2} \left(\frac{1}{\tau_v} - \frac{1}{\tau_{w_H,\text{light}}} \pm \left| \frac{1}{\tau_v} - \frac{1}{\tau_{w_H,\text{light}}} \right| \sqrt{1 - \frac{4 k_{v,\text{hetero}} k^2_{\text{MF}} k^2_{\text{PF},H} k_E k_{\text{LTD}} k_{\text{CF}} \beta_{\text{light}} \sigma^4_H}{(1 - w_{\text{PC}} k_E \tilde{w}_E)(1/\tau_v - 1/\tau_{w_H,\text{light}})^2}} \right), \tag{S86}$$

where for conciseness, we omitted that $\tau_{w_H,\text{light}}$ and τ_v are functions of σ_H^2, with the latter defined as in Eq. (S68). Since by definition all parameter values are positive, during training $\sigma_H^2 > 0$, and we assumed that Eq. (S64) holds, both eigenvalues have negative real part and the fixed point is stable if

$$\frac{k_{v,\text{hetero}} k_{\text{MF}}^2 k_E \tilde{w}_E}{(k_{\text{light}} + k_{\text{reset}}) k_{\text{PF},H}^2} = \frac{\tau_{w_H,\text{light}}(\sigma_H^2)}{\tau_v(\sigma_H^2)} < 1. \qquad \text{[S87]}$$

Note that if the post-training stability condition, Eq. (S66), is met, then this stability condition is also automatically met, because by definition $\tau_{w_H,\text{dark}} > \tau_{w_H,\text{light}}$ for all $\sigma_H^2 > 0$ (see Eq. (S82)).

Outside of the region near the fixed point, the dynamics of the weights differ between the two mechanisms. In the following sections, we evaluate the stability of the dynamics in this region for each mechanism.

Inhibitory plasticity-driven resetting For the inhibitory plasticity mechanism, we can understand the dynamics by first looking at w_H, as defined by the learning rule in Eq. (S76). During training to increase the gain, w_H will both be driven by the climbing fiber input away from its initial value along the post-training attractor, towards the line of points $(w_{H,\text{opt}}^*(v), v)$ (along which $g = g^{\text{target}}$), and by the inhibitory plasticity back towards the attractor (the v-nullcline). Thus, the nullcline for w_H lies in between these two lines. As training continues, the weight trajectory will cross the w_H-nullcline, entering a region in which both v and w_H are increasing. If the slope of the flow field along the $w_{H,\text{opt}}^*$ line is shallower than the slope of the line itself, then the trajectory will stay bounded between the $w_{H,\text{opt}}^*$ line and the w_H-nullcline, reaching the fixed point without any overshoot in the circuit gain (Fig. S3*A*,*B*). Otherwise, the trajectory will cross the $w_{H,\text{opt}}^*$ line, causing an overshoot in the gain. In this case, the trajectory will still tend toward the fixed point as long as the post-training stability condition, Eq. (S66), holds.

First, we determine the conditions under which the trajectory will reach the fixed point without any overshoot in gain. The slope of the flow field along the $w_{H,\text{opt}}^*$ line is

$$\left.\frac{\mathrm{d}v}{\mathrm{d}w_H}\right|_{(w_{H,\text{opt}}^*(v),v)} = \frac{k_{v,\text{hetero}} k_{\text{MF}}}{k_{\text{inh}} k_{\text{PF},H}} = \left.\frac{\mathrm{d}v}{\mathrm{d}w_H}\right|_{\text{post}}, \qquad \text{[S88]}$$

i.e., it is equal to the slope of trajectories during the post-training period (see Eq. (S65)). The slope of the $w_{H,\text{opt}}^*$ line is

$$\frac{\mathrm{d}}{\mathrm{d}w_H}(v_{\text{opt}}^*(w_H)) = \frac{k_{\text{PF},H} w_{\text{PC}}}{k_{\text{MF}}} = w_{\text{PC}} k_E \tilde{w}_E \cdot \frac{k_{\text{PF},H}}{k_E \tilde{w}_E k_{\text{MF}}}, \qquad \text{[S89]}$$

where we rearrange Eq. (S73) to define $v_{\text{opt}}^*(w_H)$ as the value of v that satisfies $w_{H,\text{opt}}^* = w_H$. Note that the second term in the rightmost expression above is the slope of the v-nullcline (i.e., the post-training attractor). Therefore, following the reasoning leading to the post-training stability condition in Eq. (S66), the slope of the flow field along the $w_{H,\text{opt}}^*$ line will be shallower than the slope of the line itself if

$$\frac{\tau_{w_H,\text{dark}}(\sigma_H^2)}{\tau_v(\sigma_H^2)} < w_{\text{PC}} k_E \tilde{w}_E, \qquad \text{[S90]}$$

which can also be written as

$$\frac{k_{v,\text{hetero}} k_{\text{MF}}^2}{k_{\text{reset}} k_{\text{PF},H}^2} < w_{\text{PC}}. \qquad \text{[S91]}$$

Note that, since $w_{\text{PC}} k_E \tilde{w}_E < 1$ (Eq. (S64)), this is a stricter condition than Eq. (S66).

If the condition in Eq. (S90) does not hold, then the trajectory will cross the $w_{H,\text{opt}}^*$ line. In this region, from Eq. (S61) and Eq. (S72)–Eq. (S76), the instantaneous slope of the trajectory is given by

$$\begin{aligned}\frac{\mathrm{d}v}{\mathrm{d}w_H} &\approx \frac{k_{v,\text{hetero}} k_{\text{PF},H} k_{\text{MF}} \sigma_H^2 (w_{H,\text{reset}}^* - w_H)}{k_{\text{inh}} k_{\text{PF},H}^2 \sigma_H^2 (w_{H,\text{reset}}^* - w_H) - c_H(t)(1 - w_{\text{PC}} k_E \tilde{w}_E)} \\ &= \frac{1}{1 - \frac{c_H(t)(1 - w_{\text{PC}} k_E \tilde{w}_E)}{k_{\text{inh}} k_{\text{PF},H} \sigma_H^2 (w_{H,\text{reset}}^* - w_H)}} \cdot \left.\frac{\mathrm{d}v}{\mathrm{d}w_H}\right|_{\text{post}},\end{aligned} \qquad \text{[S92]}$$

where $c_H(t)$ is as defined in Eq. (S75). Since c_H has a saturating nonlinearity, $-C k_{\text{LTD}} k_{\text{CF}} k_{\text{PF},H} < c_H \leq 0$ in the region $w_H \leq w_{H,\text{opt}}^* \leq w_{H,\text{reset}}^*$, with C as defined by Eq. (S5). Then, the slope of trajectories is bounded,

$$\frac{1}{1 + \frac{C k_{\text{LTD}} k_{\text{CF}} k_{\text{PF},H} (1 - w_{\text{PC}} k_E \tilde{w}_E)}{k_{\text{inh}} k_{\text{PF},H} \sigma_H^2 (w_{H,\text{reset}}^* - w_H)}} \cdot \left.\frac{\mathrm{d}v}{\mathrm{d}w_H}\right|_{\text{post}} < \frac{\mathrm{d}v}{\mathrm{d}w_H} \leq \left.\frac{\mathrm{d}v}{\mathrm{d}w_H}\right|_{\text{post}}, \qquad \text{[S93]}$$

where the right-hand equality holds along the $w_{H,\text{opt}}^*$ line, as we saw above. Therefore, as long as Eq. (S66) holds (i.e., the slope of post-training trajectories is shallower than the slope of the v-nullcline), trajectories during training will also have slope shallower than the v-nullcline and tend toward the fixed point.

Inhibition of instructive signals-driven resetting For the inhibition of instructive signals mechanism, we saw above (§S5.1, *Inhibition of instructive signals-driven resetting*) that post-training dynamics could become unstable if the change in w_H during the training period was too large, limiting the maximum change in gain that the circuit could stably consolidate. This was defined by the slope of trajectories in the region of weight space where the climbing fiber response was saturated, Eq. (S70), from which we found a stable region of weight space, Eq. (S71). Assuming that before training the weights start at a fixed point and that during training the learned change in gain initially only comes from changes in w_H, the largest gain change that could be learned is

$$\begin{aligned}\Delta g_{\max} &= -\frac{w_{\rm PC}k_E k_{{\rm PF},H}}{1-w_{\rm PC}k_E\tilde{w}_E}\Delta w_{H,\max}\\ &= -\frac{w_{\rm PC}k_{{\rm PF},H}^2 k_{\rm LTD}k_{\rm CF}C}{k_{v,{\rm hetero}}k_{\rm MF}^2\sigma_H^2\tilde{w}_E},\end{aligned} \quad \text{[S94]}$$

where $\Delta w_{H,\max}$ has value equal to the right hand side of Eq. (S71). With this largest gain change in mind, below we examine the weight dynamics during training.

From Eq. (S78) we can see that for a fixed value of v, $\mathrm{d}w_H/\mathrm{d}t = 0$ when

$$\begin{aligned}w_H &= \frac{\beta_{\rm light}k_E w_{\rm PC}}{\beta_{\rm light}k_E w_{\rm PC}+\beta_{\rm reset}}w^*_{H,{\rm opt}}(v) + \frac{\beta_{\rm reset}}{\beta_{\rm light}k_E w_{\rm PC}+\beta_{\rm reset}}w^*_{H,{\rm reset}}(v)\\ &= \alpha w^*_{H,{\rm opt}}(v) + (1-\alpha)w^*_{H,{\rm reset}}(v),\end{aligned} \quad \text{[S95]}$$

where α is defined as in Eq. (S81). That is, the w_H-nullcline is linear and lies between the line $(w^*_{H,{\rm opt}}(v), v)$ (along which $g = g^{\rm target}$) and the post-training attractor (v-nullcline). Thus, during training to increase the gain, initially w_H will decrease from a point on the post-training attractor toward the w_H-nullcline, during which time v will start to grow. The trajectory will then cross the w_H-nullcline. As long as the trajectory does so within the region defined by Eq. (S71), the trajectory will tend toward the post-training attractor and eventually the fixed point (Fig. S3*D*).

However, it is possible that trajectories may cross the $w^*_{H,{\rm opt}}$ line, which would correspond to the gain of the circuit overshooting the target. We can try to ensure that trajectories do not overshoot by keeping them within the region bounded by $w^*_{H,{\rm opt}}$ and the w_H-nullcline. If the $w^*_{H,{\rm opt}}$ line is near the w_H-nullcline and therefore within the linear regime of the climbing fiber response δCF, the slope of trajectories along the line is approximately as defined in Eq. (S89). As for the inhibitory plasticity reset mechanism, trajectories will therefore stay approximately within the region between the $w^*_{H,{\rm opt}}$ line and the w_H-nullcline if the condition in Eq. (S90) is met. If the $w^*_{H,{\rm opt}}$ line is far from the nullcline, and the climbing fiber response is saturated, then the slope of the flow field along the $w^*_{H,{\rm opt}}$ line is

$$\left.\frac{\mathrm{d}v}{\mathrm{d}w_H}\right|_{(w^*_{H,{\rm opt}}(v),v)} \approx \frac{k_v k_{\rm MF}\sigma_H^2}{k_{\rm LTD}k_{\rm CF}C(1-w_{\rm PC}k_E\tilde{w}_E)}(w^*_{H,{\rm reset}} - w^*_{H,{\rm opt}}). \quad \text{[S96]}$$

Then, trajectories will stay bounded by the $w^*_{H,{\rm opt}}$ line and there will be no overshoot in gain if

$$\left.\frac{\mathrm{d}v}{\mathrm{d}w_H}\right|_{(w^*_{H,{\rm opt}}(v),v)} < \frac{\mathrm{d}}{\mathrm{d}w_H}(v^*_{\rm opt}),$$

where $\mathrm{d}/\mathrm{d}w_H(v^*_{\rm opt})$ is the slope of the $w^*_{H,{\rm opt}}$ line defined in Eq. (S89). This directly gives that, for a given value of v, the distance between the $w^*_{H,{\rm opt}}$ line and the v-nullcline $w^*_{H,{\rm reset}}(v)$ can be no larger than

$$w^*_{H,{\rm reset}}(v) - w^*_{H,{\rm opt}}(v) < w_{\rm PC}k_E\tilde{w}_E\Delta w_{H,\max}. \quad \text{[S97]}$$

S5.3. Effect of post-training reset on weight dynamics. With either the inhibitory plasticity or homeostatic climbing fiber mechanism, two important properties arise in the model's dynamics that are also observed experimentally. First, the early-learning weight w_H tends toward a saturation point during training that is not simply the value that would minimize errors. In the feedforward model, this property was modeled explicitly by including a decay term in the learning rule Eq. (13) (following previous work), which we do not include in the model with internal feedback (i.e., in Eq. (27)). Second, during training, the approach of the early-learning weight to the saturation point occurs over a faster timescale than the subsequent post-training reset during consolidation (i.e., Eq. (S82); for the inhibition of instructive signals, this is true in the linear regime of the CF response). This was also modeled explicitly in the feedforward model with two different values of τ_w during the training and post-training periods, but in the model with internal feedback results from the fact that learning and resetting are being driven by two different mechanisms. Note that we could also include either of the reset mechanisms in the feedforward model (as it is the case of $w_E = 0$), and the resulting dynamics would also have these properties without having to explicitly model them.

S6. Circuit model with plastic internal feedback. In this section, we extend the feedback model discussed in the previous section (§S5) to the case in which the weight of the internal feedback pathway to the early-learning area is also plastic. Plasticity in the excitatory feedforward and feedback weights, w_H^+ and w_E^+ respectively, is described by Eq. (27). The plasticity at the late-learning site v is described, as before, by Eq. (S61). Similar to the circuits with fixed strength feedback, we can show how changes at the early-learning site can be successfully consolidated post-training if the circuit either has plasticity at w_H^-

(and/or w_E^-), or through inhibition of the climbing fiber instructive signal pathway, both of which reset early-learning activity so that the output of the early-learning area does not modulate in response to sensory input.

Below, we describe in further detail the implementation of each of these reset mechanisms in the circuit. Then, we show that the linearized dynamics of the circuit with either reset mechanism are the same, and determine conditions under which learning and consolidation occur stably. In general, learning of a target input-output gain and post-training consolidation will be locally stable around its fixed point as long as the weight of the feedback pathway w_E stays within the region $w_E < 1/(w_{\mathrm{PC}} k_E k_{\mathrm{PF},E})$, so that the strength of the feedback around the loop from early- to late-learning area and back to the early-learning area is less than 1, and if the rate of plasticity at the early-learning sites is fast compared to the rate of plasticity at the late-learning site.

S6.1. Model formulation. We implemented the two circuit reset mechanisms by extending the model with non-plastic feedback (see Materials and Methods, *Circuit model with internal feedback loop*) as follows.

Inhibitory plasticity-driven resetting For the inhibitory plasticity mechanism we model plasticity in w_H^- as governed by Eq. (29) and in w_E^- by

$$\frac{\mathrm{d}w_E^-}{\mathrm{d}t} = k_{\mathrm{inh}} \langle \mathrm{PF}_E(t) \delta\mathrm{PC}(t) \rangle_{\tau_{\mathrm{inh}}}, \quad \text{[S98]}$$

where $\delta\mathrm{PC}(t) = \mathrm{PC}(t) - \langle \mathrm{PC}(t) \rangle_{\tau_f}$. During training with a target gain of g^{target}, plasticity at the excitatory weights to the early-learning area is driven by a climbing fiber signal carrying only retinal slip, Eq. (12). Plasticity at the feedforward excitatory weight w_H^+ is driven by (negative of) the feedforward parallel fiber-climbing fiber covariance $c_H(t)$, Eq. (S75), leading to the simplified learning rule for the net weight w_H in Eq. (S76), where now $w_{H,\mathrm{opt}}^*$ and $w_{H,\mathrm{reset}}^*$ are functions of both w_E and v. Similarly, plasticity at w_E^+ is driven by (negative of) the feedback covariance

$$\begin{aligned} c_E(t) &= k_{\mathrm{LTD}} \langle \delta\mathrm{PF}_E(t) \mathrm{CF}(t) \rangle_{\tau_{f,w}} \\ &\approx -k_{\mathrm{LTD}} k_{\mathrm{CF}} k_{\mathrm{PF},E} \cdot g(t) \langle \dot{H}(t) \tanh[\beta_{\mathrm{light}}(g^{\mathrm{target}} - g(t)) \dot{H}(t)] \rangle_{\tau_{f,w}}. \end{aligned} \quad \text{[S99]}$$

As for the feedforward input, we note that

$$g^{\mathrm{target}} - g(t) = \frac{w_{\mathrm{PC}} k_E k_{\mathrm{PF},E} \cdot g^{\mathrm{target}}}{1 - w_{\mathrm{PC}} k_E \tilde{w}_E(t)} (w_{E,\mathrm{opt}}^*(w_H(t), v(t)) - w_E(t)), \quad \text{[S100]}$$

where we define

$$w_{E,\mathrm{opt}}^*(w_H, v) = \frac{1}{w_{\mathrm{PC}} k_E k_{\mathrm{PF},E}} \left[1 - \frac{k_E}{g^{\mathrm{target}}} (k_{\mathrm{MF}} v - w_{\mathrm{PC}} k_{\mathrm{PF},H} w_H) \right], \quad \text{[S101]}$$

i.e., the value of w_E that would minimize errors for a fixed choice of w_H and v. Further defining

$$w_{E,\mathrm{reset}}^*(w_H, v) = \frac{k_{\mathrm{PF},H} w_H}{k_E k_{\mathrm{PF},E} k_{\mathrm{MF}} v}, \quad \text{[S102]}$$

we can write a combined learning rule for the net feedback weight w_E,

$$\begin{aligned} \frac{\mathrm{d}w_E}{\mathrm{d}t} \approx{}& \delta_{\mathrm{train}} k_{\mathrm{LTD}} k_{\mathrm{CF}} k_{\mathrm{PF},E} \cdot g \left\langle \dot{H} \tanh \left[\beta_{\mathrm{light}} \frac{w_{\mathrm{PC}} k_E k_{\mathrm{PF},E} \cdot g^{\mathrm{target}}}{1 - w_{\mathrm{PC}} k_E k_{\mathrm{PF},E} w_E} \left(w_{E,\mathrm{opt}}^*(w_H, v) - w_E \right) \dot{H} \right] \right\rangle_{\tau_{f,w}} \\ &+ \frac{k_{\mathrm{inh}} k_{\mathrm{PF},E}^2 \sigma_H^2 \cdot g \cdot k_E k_{\mathrm{MF}} v}{1 - w_{\mathrm{PC}} k_E k_{\mathrm{PF},E} w_E} \left(w_{E,\mathrm{reset}}^*(w_H, v) - w_E \right), \end{aligned} \quad \text{[S103]}$$

where $\delta_{\mathrm{train}} = 1$ during training. Post-training, $\delta_{\mathrm{train}} = 0$ since $\dot{R} \equiv 0$, which implies that $c_H(t) = c_E(t) \equiv 0$. Similarly, post-training plasticity at w_H is governed by Eq. (S63).

Inhibition of instructive signals-driven resetting For the inhibition of instructive signals mechanism, we assume the inhibitory weight is fixed and that plasticity at both w_H^+ and w_E^+ is governed by the climbing fiber response defined in Eq. (30) (Fig. S2*A*). More specifically, plasticity at w_H^+ is driven by $c_H(t)$ as defined in Eq. (S77), and we can similarly write the feedback parallel fiber-climbing fiber covariance that drives plasticity at w_E^+ as

$$c_E(t) \approx -k_{\mathrm{LTD}} k_{\mathrm{CF}} k_{\mathrm{PF},E} \cdot g(t) \langle \dot{H}(t) \tanh[\delta_{\mathrm{train}} \beta_{\mathrm{light}} (g^{\mathrm{target}} - g(t)) \dot{H}(t) + \beta_{\mathrm{reset}} \delta\mathrm{PC}(t)] \rangle_{\tau_{f,w}}, \quad \text{[S104]}$$

where g^{target} is the target gain of the circuit during training, and $\delta_{\mathrm{train}} = 1$ during training and 0 post-training. From this, we can write that plasticity of the net weight w_E is governed by

$$\begin{aligned} \frac{\mathrm{d}w_E}{\mathrm{d}t} \approx{}& k_{\mathrm{LTD}} k_{\mathrm{CF}} k_{\mathrm{PF},E} \cdot g \left\langle \dot{H} \tanh \left[\left(\delta_{\mathrm{train}} \beta_{\mathrm{light}} \frac{w_{\mathrm{PC}} k_E k_{\mathrm{PF},E} \cdot g^{\mathrm{target}}}{1 - w_{\mathrm{PC}} k_E k_{\mathrm{PF},E} w_E} \left(w_{E,\mathrm{opt}}^*(w_H, v) - w_E \right) \right. \right. \right. \\ &\left. \left. \left. + \beta_{\mathrm{reset}} \frac{k_{\mathrm{PF},E} k_E k_{\mathrm{MF}} v}{1 - w_{\mathrm{PC}} k_E k_{\mathrm{PF},E} w_E} \left(w_{E,\mathrm{reset}}^*(w_H, v) - w_E \right) \right) \dot{H} \right] \right\rangle_{\tau_{f,w}}, \end{aligned} \quad \text{[S105]}$$

where $w_{E,\mathrm{opt}}^*$ and $w_{E,\mathrm{reset}}^*$ are defined by Eq. (S101) and Eq. (S102) above.

In Figure S2, we simulated the time evolution of the weights in the circuit implementing this latter mechanism for a 0.5 h period of training to increase the gain to $g^{\mathrm{target}} = 2$ followed by a 23.5 h post-training period. The model parameters were the same as for the model without plasticity of the feedback pathway (Tables S1–S3).

S6.2. Stability of shared linearized dynamics. Here we extend the analyses of the model without plastic feedback during the training (§S5.2) and post-training (§S5.1) periods to the case of plastic feedback.

Training We consider the system of differential equations for the three synaptic weights w_H, w_E and v from Eq. (S61) and either Eq. (S76) and Eq. (S103) for the inhibitory plasticity reset mechanism, or Eq. (S78) and Eq. (S105) for the inhibition of instructive signals reset mechanism. For either mechanism, there is a line of steady states (parameterized by s),

$$\begin{pmatrix} w_H^* \\ w_E^* \\ v^* \end{pmatrix} = \begin{pmatrix} k_{\mathrm{PF},E}/k_{\mathrm{PF},H} \cdot g^{\mathrm{target}} \\ 1 \\ 0 \end{pmatrix} s + \begin{pmatrix} 0 \\ 0 \\ g^{\mathrm{target}}/(k_E k_{\mathrm{MF}}) \end{pmatrix}, \tag{S106}$$

along which $w_H^* = w_{H,\mathrm{opt}}^* = w_{H,\mathrm{reset}}^*$ and $w_E^* = w_{E,\mathrm{opt}}^* = w_{E,\mathrm{reset}}^*$. This corresponds to the intersection of the surface in weight space that leads the circuit to produce the target gain g^{target} with the surface defined by Eq. (S62).

To analyze the stability of the fixed points defined by this line, we calculate the eigenvalues of the Jacobian of the system of differential equations. We note that the Jacobian has the same form for both reset mechanisms after substituting the relevant expressions for k_{reset}, as defined in Eq. (S84), into the differential equations. Leaving Eq. (S106) parameterized by w_E^* (i.e., taking an arbitrary value of s), we calculate the characteristic polynomial of the Jacobian, which has three roots. One of the roots is $\lambda_1 = 0$ and corresponds to the direction along the line of steady states. Dividing this root out, we are left with $\lambda^2 + a\lambda + b = 0$ for determining the remaining eigenvalues λ_2 and λ_3, where

$$a = -(\lambda_2 + \lambda_3) = \frac{\sigma_H^2}{1 - w_{\mathrm{PC}} k_E k_{\mathrm{PF},E} w_E^*} \cdot \left[(k_{\mathrm{reset}} + k_{\mathrm{light}})(k_{\mathrm{PF},H}^2 + (g^{\mathrm{target}})^2 k_{\mathrm{PF},E}^2) - k_{v,\mathrm{hetero}} k_{\mathrm{MF}}^2 k_E k_{\mathrm{PF},E} w_E^* \right] \tag{S107}$$

$$b = \lambda_2 \lambda_3 = k_{v,\mathrm{hetero}} k_E k_{\mathrm{MF}}^2 k_{\mathrm{LTD}} k_{\mathrm{CF}} \beta_{\mathrm{light}} \sigma_H^4 \frac{(g^{\mathrm{target}})^2 k_{\mathrm{PF},E}^2 + k_{\mathrm{PF},H}^2}{1 - w_{\mathrm{PC}} k_E k_{\mathrm{PF},E} w_E^*}, \tag{S108}$$

using k_{light} and k_{reset} as defined in Eq. (S83)) and Eq. (S84). For the eigenvalues to have negative real parts, we must have $a > 0$ and $b > 0$. For $w_E < 1/(w_{\mathrm{PC}} k_E k_{\mathrm{PF},E})$, since all parameters are positive and $\sigma_H^2 > 0$ during training, $b > 0$ is satisfied. $a > 0$ is satisfied if

$$\frac{1}{\tau_{w_H,\mathrm{light}}} + \frac{1}{\tau_{w_E,\mathrm{light}}} > \frac{1}{\tau_v}, \tag{S109}$$

where $\tau_{w_H,\mathrm{light}}$ and τ_v are defined as in Eq. (S82) and Eq. (S68) and depend on the value of w_E^* that parameterizes the fixed point as well as on σ_H^2, and where

$$\tau_{w_E,\mathrm{light}}(\sigma_H^2, w_E^*) = \frac{1 - w_{\mathrm{PC}} k_E k_{\mathrm{PF},E} w_E^*}{(k_{\mathrm{reset}} + k_{\mathrm{light}})(g^{\mathrm{target}} k_{\mathrm{PF},E})^2 \sigma_H^2}. \tag{S110}$$

That is, for stable learning, the sum of the instantaneous effective rates of plasticity of the early-learning sites w_H and w_E should be faster than the instantaneous effective rate of plasticity at the late-learning site v, extending the result from the fixed-strength feedback case above.

Assuming that w_E^* is upper-bounded by $1/(w_{\mathrm{PC}} k_E k_{\mathrm{PF},E})$, and assuming that the circuit is learning only positive gains so that $g^{\mathrm{target}} > 0$, we can satisfy Eq. (S109) for the whole range of w_E^* and potential gains with a stricter condition

$$\frac{k_{v,\mathrm{hetero}} k_{\mathrm{MF}}^2}{k_{\mathrm{PF},H}^2 (k_{\mathrm{light}} + k_{\mathrm{reset}})} < w_{\mathrm{PC}}. \tag{S111}$$

Post-training In the absence of information about errors, we take $\delta_{\mathrm{train}} = 0$ in Eq. (S103) and Eq. (S105). Then, the steady states of the dynamics lie along the surface represented by Eq. (S62). Calculating the eigenvalues of the Jacobian at a fixed point $(k_E \tilde{w}_E^* \tilde{v}^*/k_{\mathrm{PF},H}, w_E^*, v^*)$, parameterized by w_E^* and v^*, we find that there are two zero eigenvalues, corresponding to the surface in Eq. (S62), and a third eigenvalue

$$\lambda = -\frac{\sigma_H^2}{1 - w_{\mathrm{PC}} k_E \tilde{w}_E^*} \left(k_{\mathrm{reset}} (k_{\mathrm{PF},H}^2 + (k_{\mathrm{PF},E} k_E k_{\mathrm{MF}} v^*)^2) - k_{v,\mathrm{hetero}} k_{\mathrm{MF}}^2 k_E k_{\mathrm{PF},E} w_E^* \right), \tag{S112}$$

which is negative if $\sigma_H^2 > 0$, $w_E^* < 1/(w_{\mathrm{PC}} k_E k_{\mathrm{PF},E})$ and

$$\frac{1}{\tau_{w_H,\mathrm{dark}}} + \frac{1}{\tau_{w_E,\mathrm{dark}}} > \frac{1}{\tau_v}, \tag{S113}$$

where $\tau_{w_H,\mathrm{dark}}$ and τ_v are defined as in Eq. (S67) and Eq. (S68) and depend on the value of w_E^* that parameterizes the fixed point as well as on σ_H^2, and where

$$\tau_{w_E,\mathrm{dark}}(\sigma_H^2, w_E^*, v^*) = \frac{1 - w_{\mathrm{PC}} k_E k_{\mathrm{PF},E} w_E^*}{k_{\mathrm{reset}} (k_{\mathrm{PF},E} k_E k_{\mathrm{MF}} v^*)^2 \sigma_H^2}. \tag{S114}$$

For $w_E^* < 1/(w_{\mathrm{PC}} k_E k_{\mathrm{PF},E})$, and assuming $v^* > 0$, we can write a single stricter condition that is equivalent to Eq. (S91) to satisfy Eq. (S113). If this condition is met, then the surface of fixed points Eq. (S62) will be locally attractive during the post-training period, and since Eq. (S111) will be automatically satisfied, the dynamics during training will be locally stable around the fixed point as well.

S7. Simulation of oculomotor learning. In all simulations, we chose parameters such that the initial gain of the response was 0.4. In the simulations of the feedforward model shown in Figs. 2 and 5, we used a target gain value of $g^{\text{target}} = 2$, and chose w_H^-, k_{LTP} and k_{LTD} so that during training, w_H^+ decreased by 51% (12) and the input-to-output gain increased by 30% from baseline (13, 14). The learning rates $k_{v,\text{hetero}}$ and $k_{v,\text{Hebb}}$ were picked so that approximately 75% of the increase in gain during the training period was consolidated (14). All other model parameters are shown in Table S1.

For the perturbation simulations in Fig. 3, we initialized the weights to the same baseline as the training simulations and, every 10 minutes of simulation time, added a value chosen uniformly at random between -0.1 and 0.1 to w_H. We simulated the effect of perturbations either in the absence of (Fig. 3*A*–*D*) or in the presence of (Fig. 3*E*–*H*) information about errors. For the simulations shown in Fig. 3*A* and *E*, we used the same parameters as for the simulation in Fig. 2 (Table S1), but with $\tau_{f,v} = \tau_f$. For the simulations shown in Fig. 3*B* and *F*, we used a smaller value of $k_{v,\text{hetero}} = 6.95 \times 10^{-6}$ $(\text{s/sp})^2/\text{h}$. We performed 250 runs of the perturbation simulations with the normal and smaller values of $k_{v,\text{hetero}}$, with each simulation run for 24 hours of simulation time. The histograms in Fig. 3*C* were constructed from the endpoints of the 250 simulations in panels *A* and *B*, and we plotted the time course of the variances of the corresponding distributions (at the time point immediately before a perturbation) in Fig. 3*D*. Similarly to Fig. 3*C*, the histograms in panel *G* were generated from the final value of v across the 250 simulations in panels *E* and *F*, and panel *H* shows the time courses of the variance of v calculated in the same manner as for panel *D*. For panel *H*, the variance of v for the smaller learning rate was calculated across all four consecutive days of training (magenta curve).

For the model with efference copy feedback, we simulated the same oculomotor experiment as for the feedforward model, with differences in parameters specified in Tables S2 and S3.

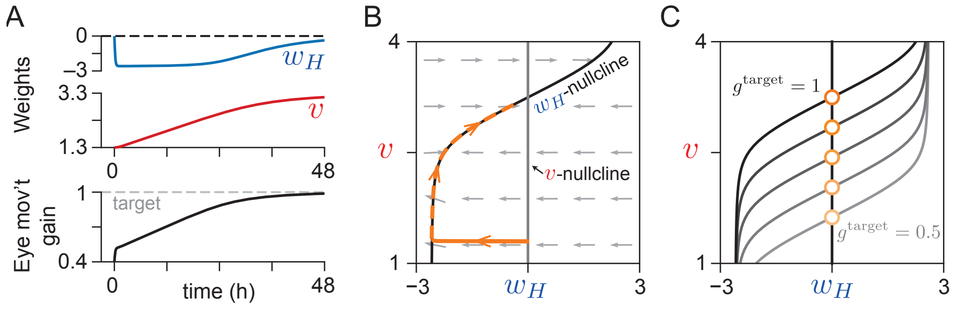

Fig. S1. Synaptic weight dynamics of the feedforward-architecture model during training. (*A*) Dynamics of weights (*top*) and the gain of the circuit (*bottom*) during 48 h of simulated training to increase the sensory input-to-motor output gain of the circuit to a target value of 1. (*B*) Trajectory (orange curve) shows the evolution of synaptic weights in *A*, which approaches the stable fixed point where the w_H- and v-nullclines (light and dark grey lines) cross. For clarity, the orange trajectory is shown as dashed where it overlaps the w_H-nullcline. (*C*) The nullclines (grey to black lines) and fixed points (orange circles) of the system for different choices of desired input-to-output gain value (shown by lightness). Any target gain value can be learned by the system, corresponding to a fixed point lying along the line $w_H = 0$. These points are thus also stable when information about behavioral errors is not present.

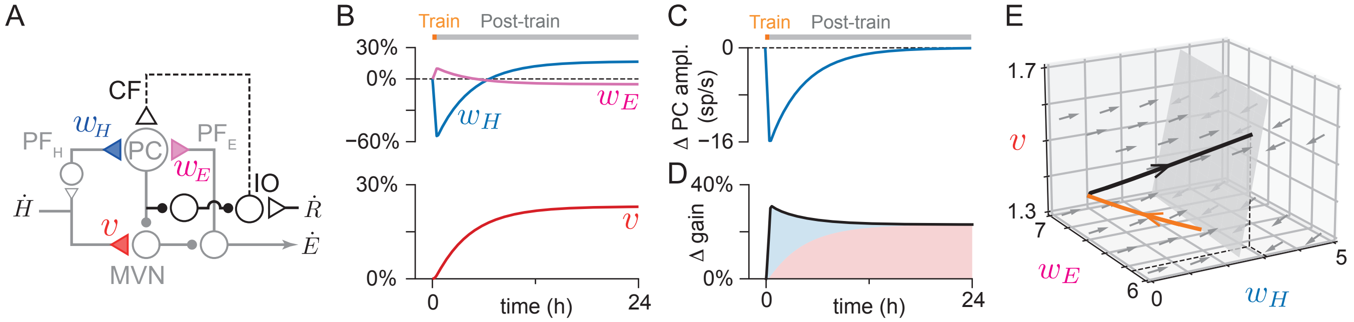

Fig. S2. Systems consolidation in a circuit with plastic internal feedback loop. (*A*) Architecture of circuit with three sites of plasticity, at the feedforward weight onto the early-learning area, w_H (blue), at the weight of the internal feedback pathway, w_E (pink), and at the direct pathway onto the late-learning area, v (red). As in Fig. 6*F*, the post-training reset was achieved by inhibition of the pathway carrying instructive signals to the early-learning area. (*B*) Change in weights during 0.5 h of simulated training to increase the gain (orange block) followed by 23.5 h post-training with no feedback about behavioral errors (grey block). (*C*) Early-learning area output (amplitude of Purkinje cell activity relative to moving baseline), which drives consolidation at the late-learning site. (*D*) Change in the gain of the eye movement response (black line). Blue and red shaded areas show the contributions of the early- and late-learning areas to the circuit transformation. (*E*) Trajectory of synaptic weights during the training (orange) and post-training (black) periods. Dashed black lines show the projection of the steady state reached at the end of training onto the w_H-w_E plane. Grey arrows show the approximate instantaneous direction in which a weight configuration at a given point in synaptic weight space will evolve during the post-training period, determined analytically. Trajectories tend toward a 2-D surface of marginally stable points (solid grey surface).

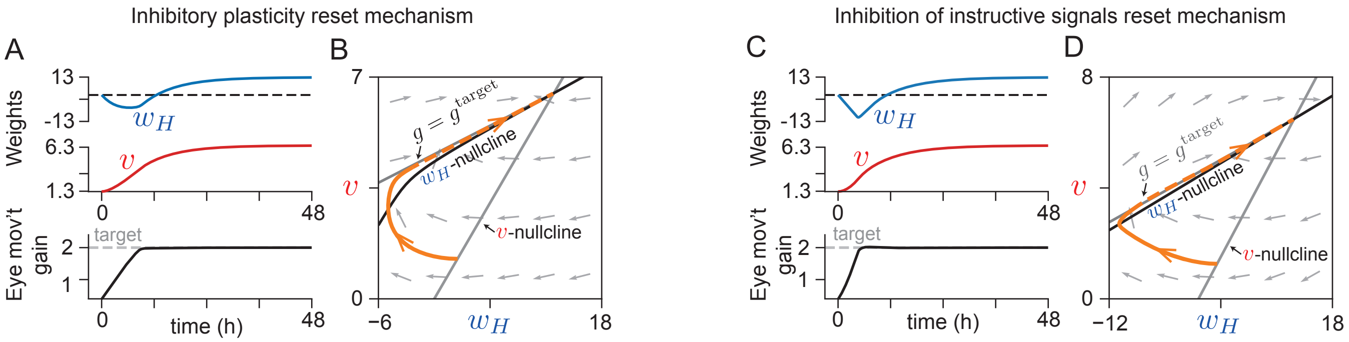

Fig. S3. Synaptic weight dynamics during training of the model with feedback architecture. (*A*) Dynamics of weights (*top*) and the gain of the circuit (*bottom*) during 48 h of simulated training to increase the sensory input-to-motor output gain of the circuit, using the inhibitory plasticity reset mechanism. (*B*) Trajectory (orange curve) shows the evolution of synaptic weights in *A*. All trajectories approach a stable fixed point where the w_H- and v-nullclines cross. The second grey line that the trajectory follows corresponds to the constant gain line for the target gain. (*C,D*) Same as *A,B*, but for the inhibition of instructive signals reset mechanism. In panels *B* and *D*, for clarity, the orange trajectory is shown as dashed where it overlaps the w_H-nullcline.

Table S1. Values and descriptions for parameters of feedforward circuit model.

Parameter	Value	Description	Source
MF_0	55 sp/s	Mossy fiber (MF) spontaneous baseline rate	Ref. 15
k_{MF}	0.14 (sp/s)/(deg/s)	MF sensitivity to vestibular input	
PF_0	14 sp/s	Parallel fiber (PF) spontaneous baseline rate	Based on ref. 16
k_{PF}	0.42 (sp/s)/(deg/s)	PF sensitivity to vestibular input	
PC_0	50 sp/s	Purkinje cell spontaneous baseline rate	Ref. 17
MVN_0	−12 sp/s	Correction factor to make medial vestibular nucleus (MVN) spontaneous baseline rate = 57 sp/s	Ref. 18
CF_0	1 sp/s	Climbing fiber (CF) spontaneous baseline rate	E.g., Refs. 19, 20
k_{CF}	1 sp/s	Maximum amplitude of CF response around baseline	
β_{light}	1 s/deg	Scale factor for saturation of CF response	
k_E	2.2 (deg/s)/(sp/s)	Eye velocity sensitivity to MVN firing	Based on ref. 18
k_{LTP}	1.005 s/sp	Contribution of parallel fiber firing to plasticity at early-learning site w_H	Based on amount of learning in refs. 13, 14
k_{LTD}	0.648 $(s/sp)^2$	Contribution of parallel fiber-climbing fiber coincidence to plasticity at w_H	
$\tau_{w,train}$	0.15 h	Time constant of plasticity at w_H during training	Ref. 14
$\tau_{w,post}$	5 h	Time constant of plasticity at w_H after training	Based on refs. 12, 21
$k_{v,hetero}$	2.75×10^{-5} $(s/sp)^2/h$	Plasticity rate at late-learning site v for heterosynaptic rule	
$k_{v,Hebb}$	8×10^{-3} $(s/sp)^2/h$	Plasticity rate at v for Hebbian rule	
τ_f	1 min	Timescale of fast average used to calculate eye movement output	
$\tau_{f,w}$	1 min	Timescale of average used in plasticity rule at w_H	
$\tau_{f,v}$	1 min (Fig. 2)<br>0.7 h (Fig. 3)	Timescale of average used in plasticity rule at v	
τ_s	0.0395 h	Timescale of sliding average θ in Hebbian rule for v	
w_H^-	5	Nonplastic molecular layer interneuron to Purkinje cell weight	Consistent with weight change in ref. 12
$w_{H,0}^+$	5	Initial value of early-learning, PF-Purkinje cell weight w_H	
w_{PC}	0.05	Purkinje cell to MVN synaptic strength	Based on ref. 22
v_0	1.3	Initial value of late-learning MF-MVN weight v	

Sources for values taken from the literature are provided in the right-hand column. Parameters that were calculated based on published values are indicated as "based on" a source.

Table S2. Values, descriptions and sources for parameters of circuit model with internal efference copy feedback, using the climbing fiber negative feedback reset mechanism.

Parameter	Value	Description	Source
$k_{\mathrm{PF},H}$	0.42 (sp/s)/(deg/s)	PF sensitivity to vestibular input	Based on ref. 16
$k_{\mathrm{PF},E}$	0.42 (sp/s)/(deg/s)	PF sensitivity to efference copy input	
PC_0	−75 sp/s	Correction factor to make Purkinje cell spontaneous baseline rate = 50 sp/s	Ref. 17
MVN_0	−9.9 sp/s	Correction factor to make MVN spontaneous baseline = 57 sp/s	Ref. 18
k_{LTD}	0.75 (s/sp)^2/h	Contribution of PF firing to plasticity at early-learning site w_H	
k_{LTP}	0.75 (s/sp)/h	Contribution of PF-CF coincidence to plasticity at w_H	
β_{light}	1 s/deg	Slope parameter for error-driven plasticity	
β_{reset}	0.0125 s/sp	Slope parameter for reset plasticity	
$k_{v,\mathrm{hetero}}$	3.6×10^{-3} (s/sp)^2/h	Plasticity rate at v	
$w^+_{H,0}$	2.5	Initial value of excitatory feedforward early-learning weight w^+_H	
w^-_H	5	Nonplastic molecular layer interneuron to Purkinje cell weight	Consistent with weight change in ref. 12
w^+_E	8.43	Nonplastic excitatory weight of efference copy input to Purkinje cell	Net weight based on ref. 17
w^-_E	2	Nonplastic inhibitory weight of efference copy input to Purkinje cell	
v_0	1.26	Initial value of late-learning weight	

All other parameters same as in Table S1.

Table S3. Values and descriptions of parameters of circuit model with internal efference copy feedback, using the inhibitory plasticity reset mechanism.

Parameter	Value	Description
k_{inh}	0.01 $(\text{s/sp})^2/\text{h}$	Maximum rate of reset plasticity
$k_{v,\text{hetero}}$	3.9×10^{-3} $(\text{s/sp})^2/\text{h}$	Plasticity rate at v
τ_{inh}	1 min	Timescale of sliding average of PC activity

All other parameters same as in Tables S1 and S2.

References

1. KD Miller, DJC MacKay, The role of constraints in Hebbian learning. *Neural Comput.* **6**, 100–126 (1994).
2. E Oja, Simplified neuron model as a principal component analyzer. *J. Math. Biol.* **15**, 267–273 (1982).
3. P Yger, M Gilson, Models of metaplasticity: A review of concepts. *Front. Comput. Neurosci.* **9**, 138 (2015).
4. EL Bienenstock, LN Cooper, PW Munro, Theory for the development of neuron selectivity: Orientation specificity and binocular interaction in visual cortex. *J. Neurosci.* **2**, 32–48 (1982).
5. F Zenke, G Hennequin, W Gerstner, Synaptic plasticity in neural networks needs homeostasis with a fast rate detector. *PLoS Comput. Biol.* **9**, e1003330 (2013).
6. F Zenke, W Gerstner, S Ganguli, The temporal paradox of Hebbian learning and homeostatic plasticity. *Curr. Opin. Neurobiol.* **43**, 166–176 (2017).
7. LF Abbott, SB Nelson, Synaptic plasticity: Taming the beast. *Nat. Neurosci.* **3 Suppl**, 1178–1183 (2000).
8. C von der Malsburg, Self-organization of orientation sensitive cells in the striate cortex. *Kybernetik* **14**, 85–100 (1973).
9. GG Turrigiano, The self-tuning neuron: Synaptic scaling of excitatory synapses. *Cell* **135**, 422–435 (2008).
10. C Tetzlaff, C Kolodziejski, M Timme, F Wörgötter, Synaptic scaling in combination with many generic plasticity mechanisms stabilizes circuit connectivity. *Front. Comput. Neurosci.* **5** (2011).
11. TP Vogels, H Sprekeler, F Zenke, C Clopath, W Gerstner, Inhibitory plasticity balances excitation and inhibition in sensory pathways and memory networks. *Science* **334**, 1569–1573 (2011).
12. DC Jang, HG Shim, SJ Kim, Intrinsic plasticity of cerebellar Purkinje cells contributes to motor memory consolidation. *The J. Neurosci.* **40**, 4145–4157 (2020).
13. RR Kimpo, JM Rinaldi, CK Kim, HL Payne, JL Raymond, Gating of neural error signals during motor learning. *eLife* **3**, e02076 (2014).
14. ES Boyden, JL Raymond, Active reversal of motor memories reveals rules governing memory encoding. *Neuron* **39**, 1031–1042 (2003).
15. DM Lasker, GC Han, HJ Park, LB Minor, Rotational responses of vestibular–nerve afferents innervating the semicircular canals in the C57BL/6 mouse. *J. Assoc. for Res. Otolaryngol.* **9**, 334–348 (2008).
16. A Arenz, RA Silver, AT Schaefer, TW Margrie, The contribution of single synapses to sensory representation in vivo. *Science* **321**, 977–980 (2008).
17. A Katoh, SL Shin, RR Kimpo, JM Rinaldi, JL Raymond, Purkinje cell responses during visually and vestibularly driven smooth eye movements in mice. *Brain Behav.* **5**, e00310 (2015).
18. M Beraneck, KE Cullen, Activity of vestibular nuclei neurons during vestibular and optokinetic stimulation in the alert mouse. *J. Neurophysiol.* **98**, 1549–1565 (2007).
19. HHLM Goossens, et al., Simple spike and complex spike activity of floccular Purkinje cells during the optokinetic reflex in mice lacking cerebellar long-term depression. *Eur. J. Neurosci.* **19**, 687–697 (2004).
20. J Maruta, RA Hensbroek, JI Simpson, Intraburst and interburst signaling by climbing fibers. *J. Neurosci.* **27**, 11263–11270 (2007).
21. SF Cooke, PJE Attwell, CH Yeo, Temporal properties of cerebellar-dependent memory consolidation. *J. Neurosci.* **24**, 2934–2941 (2004).
22. HL Payne, et al., Cerebellar Purkinje cells control eye movements with a rapid rate code that is invariant to spike irregularity. *eLife* **8**, e37102 (2019).
